# Supplementary material for: An endoribonuclease-based feedforward controller for decoupling resource-limited genetic modules in mammalian cells
Source: Nat Commun. 2020 Nov 10;11:5690. doi: 10.1038/s41467-020-19126-9 (PMC7656454; doi:10.1038/s41467-020-19126-9)
Supplement: Supplementary file 1 — Reporting Summary [file 41467_2020_19126_MOESM1_ESM.pdf]

nature research

Corresponding author(s): Domitilla Del Vecchio

Last updated by author(s): Sep 15, 2020

Reporting Summary

Nature Research wishes to improve the reproducibility of the work that we publish. This form provides structure for consistency and transparency in reporting. For further information on Nature Research policies, see Authors & Referees and the Editorial Policy Checklist .

Statistics

For all statistical analyses, confirm that the following items are present in the figure legend, table legend, main text, or Methods section.

n/a	Confirmed	
☐	☒	The exact sample size (*n*) for each experimental group/condition, given as a discrete number and unit of measurement
☐	☒	A statement on whether measurements were taken from distinct samples or whether the same sample was measured repeatedly
☒	☐	The statistical test(s) used AND whether they are one- or two-sided<br>*Only common tests should be described solely by name; describe more complex techniques in the Methods section.*
☒	☐	A description of all covariates tested
☐	☒	A description of any assumptions or corrections, such as tests of normality and adjustment for multiple comparisons
☐	☒	A full description of the statistical parameters including central tendency (e.g. means) or other basic estimates (e.g. regression coefficient) AND variation (e.g. standard deviation) or associated estimates of uncertainty (e.g. confidence intervals)
☐	☒	For null hypothesis testing, the test statistic (e.g. *F*, *t*, *r*) with confidence intervals, effect sizes, degrees of freedom and *P* value noted<br>*Give P values as exact values whenever suitable.*
☒	☐	For Bayesian analysis, information on the choice of priors and Markov chain Monte Carlo settings
☒	☐	For hierarchical and complex designs, identification of the appropriate level for tests and full reporting of outcomes
☐	☒	Estimates of effect sizes (e.g. Cohen's *d*, Pearson's *r*), indicating how they were calculated

Our web collection on statistics for biologists contains articles on many of the points above.

Software and code

Policy information about availability of computer code

Data collection	FACSDiva version 8.0.1 software was used to collect flow cytometry data.
Data analysis	Excel 2016, Geneious Prime 2020.0.2, CpG Islands v1.1 (Geneious Plugin), MATLAB 2018a. Custom MATLAB code (https://github.com/Weiss-Lab/MATLAB_Flow_Analysis, version v0.3-beta, compatible with MATLAB 2018a+) was used in the analysis of flow cytometry data and more generally to create most plots.

For manuscripts utilizing custom algorithms or software that are central to the research but not yet described in published literature, software must be made available to editors/reviewers. We strongly encourage code deposition in a community repository (e.g. GitHub). See the Nature Research guidelines for submitting code & software for further information.

Data

Policy information about availability of data

All manuscripts must include a data availability statement. This statement should provide the following information, where applicable:

- Accession codes, unique identifiers, or web links for publicly available datasets
- A list of figures that have associated raw data
- A description of any restrictions on data availability

Data availability: Source Data accompanying this manuscrip includes Genbank sequences for all plasmids used in this study, primer sequences, cloning details, measured median expression levels, fit parameter values, simulation parameter values, qPCR analysis calculations, the number of cells per distribution plot, and details about plasmid dosages in each transfection. New plasmids used in this study are available for distribution from Addgene (http://www.addgene.org/Ron_Weiss/). Raw .fcs files are available from the corresponding authors upon reasonable request.

Code availability: General MATLAB code for use in .fcs file processing and analysis are available under an open-source license in our GitHub repository at https://github.com/Weiss-Lab/MATLAB_Flow_Analysis. Specific .m scripts for each experiment are available from the corresponding authors upon reasonable request.

Field-specific reporting

Please select the one below that is the best fit for your research. If you are not sure, read the appropriate sections before making your selection.

☒ Life sciences ☐ Behavioural & social sciences ☐ Ecological, evolutionary & environmental sciences

For a reference copy of the document with all sections, see nature.com/documents/nr-reporting-summary-flat.pdf

Life sciences study design

All studies must disclose on these points even when the disclosure is negative.

Sample size	A sample size of n = 3 (three experimental repeats) was chosen because it is standard practice in the field.
Data exclusions	Two data exclusions were performed: (1) in Supp. Figure 4, we excluded a data point (shown in red) from a qPCR experiment because it was several standard deviations away from the mean of the remaining points, clearly indicating that is is an outlier/experimental aritfact. (2) In Supp. Figure 12, we excluded samples with the TK promoter in HeLa cells from the clustering analysis (as shown by the blank boxes) because the nominal expression level of the TK promoter is undetectable in these cells, which we show in Supp. Figure 43 causes the knockdown by resource loading to not be measurable. Neither of these criteria were pre-established.
Replication	All attempts at replication were successful. Experimental repeats were performed at least one week apart.
Randomization	N/A: Experiments were performed on immortalized cell lines which can reasonably be assumed to be identical when split into multiple wells for trasnfection/infection.
Blinding	N/A: Success metrics for our controller design vs the unregulated system were pre-defined (fold-changes and robustness scores).

Reporting for specific materials, systems and methods

We require information from authors about some types of materials, experimental systems and methods used in many studies. Here, indicate whether each material, system or method listed is relevant to your study. If you are not sure if a list item applies to your research, read the appropriate section before selecting a response.

Materials & experimental systems

n/a	Involved in the study	
☐	☒	Antibodies
☐	☒	Eukaryotic cell lines
☒	☐	Palaeontology
☒	☐	Animals and other organisms
☒	☐	Human research participants
☒	☐	Clinical data

Methods

n/a	Involved in the study	
☒	☐	ChIP-seq
☐	☒	Flow cytometry
☒	☐	MRI-based neuroimaging

Antibodies

Antibodies used	PE/Dazzle™ 594 anti-human Ki-67: BioLegend #350533, clone Ki-67.<br>PE/Dazzle™ 594 IgG1 kappa Isotype Control, BioLegend #400177, clone MOPC-21.<br>Alexa Fluor® 594 anti-HA.11 Epitope Tag: BioLegend #901511, clone 16B12.
Validation	PE/Dazzle™ 594 anti-human Ki-67: statement by BioLegend: "Flow cytometric analysis of intracellularly-stained cells (ICFC) - Quality tested".<br>Alexa Fluor® 594 anti-HA.11 Epitope Tag: statement by BioLegend: "This antibody is effective for use in immunofluorescence (IF)".

Eukaryotic cell lines

Policy information about cell lines

Cell line source(s)	HEK-293 cells: ATCC, HEK-293FT cells: Thermo Fisher, HeLa cells: ATCC, Vero 2.2 cells: Massachusetts General Hospital, CHO-K1 cells: ATCC, U2OS cells: ATCC
Authentication	None of the cell lines were authenticated.
Mycoplasma contamination	All cell lines tested negative for mycoplasma.

Commonly misidentified lines (See ICLAC register)	No commonly misidentified cells were used in this study.

Flow Cytometry

Plots

Confirm that:

- [x] The axis labels state the marker and fluorochrome used (e.g. CD4-FITC).
- [x] The axis scales are clearly visible. Include numbers along axes only for bottom left plot of group (a 'group' is an analysis of identical markers).
- [x] All plots are contour plots with outliers or pseudocolor plots.
- [x] A numerical value for number of cells or percentage (with statistics) is provided.

Methodology

Sample preparation	Cells were prepared for flow cytometry by trypsinization followed by re-suspension in FACS buffer (PBS supplemented with 1% BSA, 5 mM EDTA, and 0.1% sodium azide).
Instrument	For the data shown in Figure 1 and SI Figures 3, 4, 6, & 8, samples were collected on a BD LSR II cytometer.<br>For all other data, samples were collected on a BD LSR Fortessa.
Software	FACSDiva version 8.0.1 software was used to collect flow cytometry data. Custom MATLAB code (https://github.com/Weiss-Lab/MATLAB_Flow_Analysis) was used in the analysis.
Cell population abundance	Sorting was not performed. All cells were grown in monocultures and were thus assumed to be pure populations. Typically, 50-80% of cells passed morphological gating depending on the sample and cell line. If an abnormally low percent of cells passed morphological gating, the experiment (or a subset of the samples if <10% of total # of samples) was re-run.
Gating strategy	Cells were separated from debris by gating on forward scatter (FSC) vs side scatter (SSC) area measurements. Further gates on FSC and SSC height vs width measurements isolated single cells. Gates for specific channels were manually set such that <1% of cells not expressing the given marker/reporter pass the gate.

- [x] Tick this box to confirm that a figure exemplifying the gating strategy is provided in the Supplementary Information.
