# Supplementary material for: An endoribonuclease-based feedforward controller for decoupling resource-limited genetic modules in mammalian cells
Source: Nat Commun. 2020 Nov 10;11:5690. doi: 10.1038/s41467-020-19126-9 (PMC7656454; doi:10.1038/s41467-020-19126-9)
Supplement: Supplementary file 2 — Supplementary Information [file 41467_2020_19126_MOESM2_ESM.pdf]

Supplementary Information: An endoribonuclease-based feedforward controller for decoupling resource-limited genetic modules in mammalian cells

Ross D. Jones[1, 2], Yili Qian[2, 3], Velia Siciliano[1, 2 †], Breanna DiAndreth[1, 2], Jin Huh[1, 2], Ron Weiss[1, 2, 4 *], and Domitilla Del Vecchio[2, 3 *]

[1]Department of Biological Engineering, Massachusetts Institute of Technology, Cambridge, MA, 02139, USA.

[2]Synthetic Biology Center, Massachusetts Institute of Technology, Cambridge, MA, 02139, USA.

[3]Department of Mechanical Engineering, Massachusetts Institute of Technology, Cambridge, MA, 02139, USA.

[4]Department of Electrical Engineering and Computer Science, Massachusetts Institute of Technology, Cambridge, MA, 02139, USA.

[†]Current address: Instituto Italiano di Tecnologia, Napoli, NA, 80125, Italy.

[*]Correspondence should be addressed to R.W. (rweiss@mit.edu) or D.D.V. (ddv@mit.edu).

Contents

1 Supplementary Note 1: Making accurate measurements of transient transfections in light of resource sharing 3

1.1 Comparison of flow cytometry gating strategies to measure effects of resource loading 3

1.2 Sensitivity of measuring weaker promoters by flow cytometry 5

2 Supplementary Note 2: A mathematical model of resource sharing in mammalian cells 6

2.1 Mathematical model for a module with TA input 6

2.2 Mathematical model for a module with constitutive expression 10

2.3 Mathematical model for a network with resource limitations 11

2.4 Effects of TA toxicity on gene expression levels 13

3 Supplementary Note 3: Experimental validation of the resource sharing model 15

3.1 Identification of the source of resource loading 15

3.2 Application of the model to a simple 2-module system 16

3.3 Prediction of CMV/UAS expression across DNA dosages using the model 18

3.4 Increases in constitutive promoter expression caused by Gal4-activators 19

3.5 A model-driven definition for TA ‘strength’ 20

4 Supplementary Note 4: Response of lentiviral-integrated promoters to resource loading 22

4.1 Non-target TRE promoter 22

4.2 On-target UAS promoter 24

5 Supplementary Note 5: Model of the endoRNase-based iFFL 26

5.1 Model formulation 26

5.2 Detailed comparison of miRNA- and endoRNase-based iFFL models 30

5.3 Dynamics of the iFFL in the presence of DNA plasmid dilution 35

5.4 Effect of growth inhibition by TAs on iFFL performance metrics 36

5.5 Alleviation of growth inhibition with a less toxic transfection reagent 38

6 Supplementary Figures 39

7 Supplementary Tables 95

1 Supplementary Note 1: Making accurate measurements of transient transfections in light of resource sharing

A fundamental issue for measurement in the context of resource sharing is that transfection markers (TX Markers) are frequently knocked down[1]. The majority of combinations of constitutive promoters and transcriptional activators (TAs) that we have evaluated yielded knockdown of the promoter (Figure 2 & Supplementary Figure 18). Thus, the effects of resource sharing make it important to consider how transfection data is measured and normalized.

1.1 Comparison of flow cytometry gating strategies to measure effects of resource loading

Analysis of transfection data follows a few standard pipelines:

- Bulk measurements (*e.g.* qPCR, luminescence)
  1. Measure a constitutively-expressed signal and an output signal (for qPCR: different probes, for luminescences: different luminescent proteins)
  2. Normalize by dividing the output signal by the constitutive signal
- Single-cell measurements (*e.g.* flow cytometry, imaging)
  1. Computationally isolate live single cells from debris, dead cells, and doublets
  2. Gate cells positive for one or more fluorescent reporters
  3. Compute statistics on the gated cells
  4. Normalize statistical measurements by a constitutive TX Marker
  5. Advanced analysis
     (a) Bin cells based on TX Marker levels
     (b) Compute statistics in each bin
     (c) Fit models to binned data (or the full distribution) assuming the TX Marker is exactly proportional to DNA dosage

As shown above, constitutively-expressed TX Markers are frequently used to normalize signals among samples via dividing the output measurement by the TX Marker measurement. However, when resources are significantly loaded, the TX Marker is no longer a reliable reporter of DNA dosage; changes in TX Marker levels may result from resource loading or other context-specific behavior, rather than from differences in transfection efficiency or DNA uptake. We thus evaluated different methods of gating and normalizing data to more robustly measure expression levels. We focus on analysis of single-cell data because bulk measurements have limited data processing capabilities.

Following are a list of single-cell gating strategies and considerations for their use:

- **Use all cells (whether transfected or not)**
  - This does not account for transfection efficiency differences among samples.
- **Gate on a constitutive TX Marker**
  - The TX Marker itself is affected by resource loading, so some cells may have their expression reduced below or raised above the limit of detection. This causes over- or under-estimation of the output expression level, respectively.
  - Outputs that are brighter than the TX Marker will be over-estimated due to cells with undetectable TX Marker but positive for the output not passing the gate.
- **Gate on a constitutive TX Marker OR the output reporter of interest**
  - This helps to normalize for both differences in transfection efficiency and for different relative expression levels of the output among samples.
  - This still suffers from resource loading affecting TX Marker and output reporter levels relative to the gate boundaries.
- **Gate on any fluorescent reporter**
  - In samples with differential expression of one or more reporters that are neither the output of interest nor the TX Marker (which is presumably transfected into each cell at the same dosage), the percent of cell passing the gate will vary.
  - In particular, in samples with relatively high expression of a reporter we may under-estimate the measurements of the output and TX Marker due to cells that would otherwise be considered untransfected due to undetectable TX Marker or output levels passing the gate.
- **Gate on a fixed percentile of the TX Marker**
  - This solves the problem of resource loading shifting reporter outputs relative to the gate boundary, but does not account for differences in transfection efficiency that may occur due to pipetting error or differential growth/death of transfected cells across samples.

A comparison of different gating strategies using the data in Figure 2 is shown in Supplementary Figure 42a. The results show that in general, transfection with Gal4 TAs reduces the number of cells passing gates for morphology (forward/side scatter) and constitutive reporters. The resulting fold-change measurements for the first four options

listed above are shown in Supplementary Figure 42b. Gating only by morphology severely under-estimates the effect of resource loading for weaker promoters and cell lines with lower transfection efficiencies (*e.g.* CHO-K1 and Vero 2.2 cells). Gating on just TX Marker or on either TX Marker or {P}:Output$_1$ yielded similar fold-changes. While gating on {P}:Output$_1$ reduces consistency in the percent of cells passing the gate between samples with different consitutive promoters (because of the difference in their strengths), it increases the consistency between samples with the same constitutive promoters but different Gal4 TAs (especially for strong constitutive promoters, see Supplementary Figure 42a). Gating on all reporters caused decreased the values of the $\log_2$ fold-changes in the level of {P}:Output$_1$ in response to Gal4 TAs. This decrease is likely because gating on UAS:Output$_2$ causing cells with low but nonzero amounts of UAS:Output$_2$ and undetectable levels of CMV:Output$_1$ to pass the gates, whereas in the Gal4-None sample the same subpopulation of cells is not captured by the gates. Based on these results, we reported data for cells gated positive for either the TX Marker or {P}:Output$_1$ in Figure 2. Note that Figure 2 also incorporates an additional autofluorescence background subtraction described below in Section 1.2.

Overall, these results show that the choice of gating method can significantly affect measurements. TX Markers cannot be blindly trusted and should be tested in combination with genetic devices used in a given experiment to determine if the devices significantly affect the TX Marker's expression level. If a TX Marker's promoter is affected by transcriptional resource sharing in a circuit, our data in Figure 2d can be used to optimally select promoters that are less affected by specific TAs.

1.2 Sensitivity of measuring weaker promoters by flow cytometry

For the data in Figure 2, our measurements of the effect of resource loading by Gal4 TAs on weaker promoters was limited due to the nominal expression levels of the promoters being nearly undetectable. This problem is illustrated in Supplementary Figure 43. In samples with weaker promoters (*e.g.* TK and RSV), we frequently saw that less than 30% of the cells that were positive for the TX Marker were also positive for {P}:Output$_1$ (Supplementary Figure 43a). To understand how close the expression level of these promoters was to the detection limit, we compared their levels to cellular autofluorescence, which we defined as the median of the untransfected cells. As with the measurement of the medians of the reporters, the median autofluorescence level was also only computed for cells with positive fluorescent values, causing it to be non-zero (autofluorescence subtraction in the data processing pipeline makes the median level of untransfected cells zero, but only if the negative values are included). We compared the relative magnitude of autofluorescence to the median of {P}:Output$_1$ in each sample, finding that for weak promoters, the autofluorescence was often between 25-75% of {P}:Output$_1$ (Supplementary Figure 43b). In particular, we identified that the expression of the TK promoter in HeLa cells with our experimental conditions was undetectable (Supplementary Figure 43c). For this reason, the samples with TK promoters in HeLa cells were

excluded from the analyses in Figure 2d & Supplementary Figure 12b-c.

The fold-difference between the autofluorescent background and the nominal level of {P}:Output_1 sets a limit on the maximal fold-change that can be detected, biasing the measurement of knockdown of weak promoters by resource loading. To reduce this bias, we subtracted the autofluorescence level (computed as described above) from the initial measurement of the median {P}:Output_1, then re-computed fold-changes, yielding Figure 2c. The fold-changes without autofluorescence subtraction are shown in Supplementary Figure 42b (panel M-YR). The differences in the log_2 fold-changes with and without autofluorescence subtraction are shown in Supplementary Figure 44a. Compared to the samples without autofluorescence subtraction, there was less correlation between the strength of a promoter and its fold-change (Supplementary Figure 44b), suggesting that the autofluorescence subtraction was able to reduce the measurement bias.

2 Supplementary Note 2: A mathematical model of resource sharing in mammalian cells

Here we establish an ordinary differential equation (ODE)-based gene expression model that captures limitations of transcriptional and translational resources in a general gene network in mammalian cells. The network consists of a set of N modules. Each module contains a series of biomolecular reactions that express a gene and produce a protein as output. We divide these modules into two complementary sets, depending on whether gene expression in the module is constitutive or transcriptionally regulated.

(I) The set $\mathcal{A}$ contains modules where the presence of a transcriptional activator (TA) is needed for the gene's expression.

(II) The set $\mathcal{B}$ contains modules in which the gene is constitutively expressed.

In the following subsections, we first develop mathematical models for expression of a single module from each of the two sets. This allows us to quantitatively characterize (a) the output from each module as a function of resource availability and (b) the resource demand from each module. Based on these results, we then derive a model for a general gene network composed of modules from both sets, taking into account that they share a common pool of available resources.

2.1 Mathematical model for a module with TA input

A module $i \in \mathcal{A}$ takes a TA (u_i) as input, which binds with the enhancer region upstream of the promoter (D_i). The activator recruits transcription coactivator proteins (CoAs, R_{TX}), chiefly the mediator complex, to the DNA to

assemble the pre-initiation complex (PIC, C_i), in the presence of other general transcriptional factors and RNA polymerase II (RNAP). Since previous experiments have shown that neither the addition of general transcription factors nor RNAP relieves squelching[2], we do not explicitly model these species in the formation of PIC and only consider recruitment of the CoA R_{TX} as the rate-limiting step.

There is strong experimental evidence suggesting that the TA and CoA (specifically, the mediator) can bind both on and off the promoter[3,4]. However, there is no experimental evidence suggesting that the CoA can bind directly to DNA without a pre-bound TA. We therefore model the formation of PIC through the following chemical reactions:

$$n \cdot u_i + D_i \underset{k_{i1}^-}{\overset{k_{i1}^+}{\rightleftharpoons}} c_{i1}, \qquad u_i + R_{TX} \underset{k_{i2}^-}{\overset{k_{i2}^+}{\rightleftharpoons}} c_{i2}, \qquad c_{i1} + R_{TX} \underset{k_{i2}^-}{\overset{k_{i2}^+}{\rightleftharpoons}} C_i, \tag{1}$$

where c_{i1} is the complex formed by the TA's DNA-binding domain (DBD) binding with its target promoter, c_{i2} is the complex formed by the TA's activation domain (AD) binding with CoA, and n represents the cooperativity of TA-promoter interaction. We assume that the association and dissociation constants of TA u_i with CoA R_{TX} (*i.e.*, $k_{i2}^{\pm}$) are independent of whether the TA is pre-bound to the DNA (*i.e.*, c_{i1}) or free in solution (*i.e.*, u_i). The PIC can then open the DNA, allowing RNAP to transcribe the mRNA m_i, which we model as a one-step enzymatic reaction:

$$C_i \xrightarrow{\varphi_i} C_i + m_i. \tag{2}$$

Once the mRNA is processed (*e.g.*, capping, cleavage, splicing and transportation to the cytoplasm), a set of eukaryotic initiation factors (eIFs) promotes the formation of ribosomal initiation complex near the start codon, allowing ribosomes to translate the mRNA[5]. While it is generally believed that translational initiation, rather than elongation, is the rate-limiting step in mammalian translation[6], the exact limiting resources have not been consolidated, with eIF-4F complex being the primary suspect in certain physiological conditions[7,8]. Instead of modeling the binding of all eIFs and the ribosome with mRNA, we assume that a single translational resource R_{TL} is limited and it binds with mRNA to form a translation initiation complex (TIC) M_i. While this assumption increases tractability of the model and allows it to capture competition for different types of limiting resources arising from different physiological conditions, the resultant model may not be able to capture simultaneous competition for multiple eIFs and/or ribosomal subunits, which is a subject of future research. Translation of mRNA m_i produces protein x_i, which we consider as the output of module i and is subject to decay. We model initiation and elongation of mRNA a using the following reactions:

$$m_i + R_{TL} \underset{\kappa_i^-}{\overset{\kappa_i^+}{\rightleftharpoons}} M_i, \qquad M_i \xrightarrow{\beta_i} M_i + x_i, \qquad m_i \xrightarrow{\delta_i} \varnothing, \qquad M_i \xrightarrow{\omega_i} R_{TL}, \qquad x_i \xrightarrow{\gamma_i} \varnothing. \tag{3}$$

Based on mass-action kinetics, the reactions in (1)-(3) give rise to the following ODEs:

$$\frac{\mathrm{d}}{\mathrm{d}t}c_{i1} = k_{i1}^{+}(u_i)^n D_i - k_{i1}^{-}c_{i1} - k_{i2}^{+}c_{i1}R_{\mathrm{TX}} + k_{i2}^{-}C_i, \tag{4}$$

$$\frac{\mathrm{d}}{\mathrm{d}t}c_{i2} = k_{i2}^{+}u_i R_{\mathrm{TX}} - k_{i2}^{-}c_{i2}, \tag{5}$$

$$\frac{\mathrm{d}}{\mathrm{d}t}C_i = k_{i2}^{+}c_{i1}R_{\mathrm{TX}} - k_{i2}^{-}C_i, \tag{6}$$

$$\frac{\mathrm{d}}{\mathrm{d}t}m_i = \varphi_i C_i - \kappa_i^{+}m_i R_{\mathrm{TL}} + \kappa_i^{-}M_i - \delta_i m_i, \tag{7}$$

$$\frac{\mathrm{d}}{\mathrm{d}t}M_i = \kappa_i^{+}m_i R_{\mathrm{TL}} - \kappa_i^{-}M_i - \omega_i M_i, \tag{8}$$

$$\frac{\mathrm{d}}{\mathrm{d}t}x_i = \beta_i M_i - \gamma_i x_i. \tag{9}$$

We assume that PIC formation is on a faster timescale than the mRNA and protein dynamics and therefore set the dynamics of $\mathrm{c_{i1}}, \mathrm{c_{i2}}$ and $\mathrm{C_i}$ to quasi-steady state (QSS). To compute the QSS concentrations of these complexes, we define the following lumped parameters:

$$k_{i1} := \left(\frac{k_{i1}^{-}}{k_{i1}^{+}}\right)^{1/n}, \qquad k_{i2} := \frac{k_{i2}^{-}}{k_{i2}^{+}}. \tag{10}$$

By setting the time derivatives in equations (4)-(6) to 0, we obtain at QSS:

$$c_{i1} = D_i\left(\frac{u_i}{k_{i1}}\right)^n, \qquad c_{i2} = \frac{u_i R_{\mathrm{TX}}}{k_{i2}}, \qquad C_i = \frac{c_{i1}R_{\mathrm{TX}}}{k_{i2}}. \tag{11}$$

To compute the free amount of DNA D_i, we use the fact that the total amount of DNA of gene i, D_i^t, is conserved:

$$D_i^t = D_i + c_{i1} + C_i,$$

and substituting in the results in (11) to obtain:

$$D_i = \frac{D_i^t}{1 + \left(\frac{u_i}{k_{i1}}\right)^n\left(1 + \frac{R_{\mathrm{TX}}}{k_{i2}}\right)}, \tag{12}$$

Substituting (12) back into (11), we obtain the QSS of PIC concentration as

$$C_i = \frac{D_i^t \frac{R_{\mathrm{TX}}}{k_{i2}} \cdot \left(\frac{u_i}{k_{i1}}\right)^n}{1 + \left(\frac{u_i}{k_{i1}}\right)^n\left(1 + \frac{R_{\mathrm{TX}}}{k_{i2}}\right)} \tag{13}$$

Note that from (13), the maximum amount of PIC, $R_{\mathrm{TX}}D_i^t/k_{i2}$, is determined by (i) the free amount of transcriptional resources (R_{TX}), (ii) the DNA copy number of the gene in module i (D_i^t), and (iii) the ability of the activator to bind

with transcriptional resources, which is quantified by the dissociation constant k_{i2}.

Similar to bacterial systems, in mammalian cells, the half-life of an mRNA is typically much shorter than that of a protein, with typical values of ~10 hrs for the former and ~45 hrs for the latter[9]. Therefore, we set the dynamics of mRNA and the TIC in equations (7)-(8) to QSS to obtain

$$m_i = \frac{\varphi_i C_i}{\delta_i + \omega_i \frac{R_{\mathrm{TL}}}{\kappa_i}}, \qquad M_i = \frac{\varphi_i C_i R_{\mathrm{TL}}}{\delta_i \kappa_i + \omega_i R_{\mathrm{TL}}}, \tag{14}$$

where we have defined:

$$\kappa_i := \frac{\kappa_i^- + \omega_i}{\kappa_i^+}$$

as a dissociation constant quantifying the binding strength between mRNA m_i and translational resource R_{TL}. Assuming that

$$\omega_i R_{\mathrm{TL}} \ll \delta_i \kappa_i, \tag{15}$$

the amount of mRNA and TIC in (14) can be approximated as:

$$m_i \approx \frac{\varphi_i C_i}{\delta_i}, \qquad M_i \approx \frac{\varphi_i C_i R_{\mathrm{TL}}}{\delta_i \kappa_i} = \frac{\varphi_i D_i^t R_{\mathrm{TL}} \frac{R_{\mathrm{TX}}}{k_{i2}} \cdot \left(\frac{u_i}{k_{i1}}\right)^n}{\delta_i \kappa_i \left[1 + \left(\frac{u_i}{k_{i1}}\right)^n \left(1 + \frac{R_{\mathrm{TX}}}{k_{i2}}\right)\right]}, \tag{16}$$

where we have used the result in (13). Substituting equation (16) into protein dynamics (9), we obtain the simplified module dynamics as

$$\frac{\mathrm{d}}{\mathrm{d}t} x_i = \frac{\varphi_i \beta_i D_i^t R_{\mathrm{TL}} R_{\mathrm{TX}} \cdot \left(\frac{u_i}{k_{i1}}\right)^n}{\delta_i \kappa_i k_{i2} \left[1 + \left(\frac{u_i}{k_{i1}}\right)^n \left(1 + \frac{R_{\mathrm{TX}}}{k_{i2}}\right)\right]} - \gamma_i x_i. \tag{17}$$

Note that in (17), the production rate of protein x_i is proportional to (i) the transcription rate constant φ_i, (ii) the translation rate constant β_i, (iii) the free amount of transcriptional resource (most likely the mediator) R_{TX}, (iv) the free amount of translational resource (most likely eIFs), (v) DNA copy number D_i^t, (vi) the mRNA decay rate constant δ_i, (vii) the dissociation constant between TA input u_i and transcriptional resource k_{i2}, (viii) the dissociation constant between mRNA m_i and translational resource κ_i, and (ix) transcription activation, which is a function $f_i(u_i, R_{\mathrm{TX}}) \in [0, 1]$ of both u_i and R_{TX}:

$$f_i(u_i, R_{\mathrm{TX}}) := \frac{\left(\frac{u_i}{k_{i1}}\right)^n}{1 + \left(\frac{u_i}{k_{i1}}\right)^n \left(1 + \frac{R_{\mathrm{TX}}}{k_{i2}}\right)}.$$

In a network context, the free amount of resources R_{TX} and R_{TL} will depend on resource demands from all modules in the network. Here we quantify first the concentration of transcriptional and translational resources demanded at module i. These quantities will subsequently be used in Section 2.3 to compute the free amount of resources in the network.

The total concentration of transcriptional resources demanded at module i, which we denote as R^i_{TX}, includes the amount of all complexes formed with $\mathrm{R_{TX}}$ in module i:

$$R^i_{\mathrm{TX}} = c_{i2} + C_i = \frac{R_{\mathrm{TX}}}{k_{i2}} \cdot \left(\frac{D^t_i \left(\frac{u_i}{k_{i1}}\right)^n}{1 + \left(\frac{u_i}{k_{i1}}\right)^n \cdot \left(1 + \frac{R_{\mathrm{TX}}}{k_{i2}}\right)} + u_i \right) = \frac{R_{\mathrm{TX}}}{k_{i2}} \cdot (D^t_i f_i(u_i, R_{\mathrm{TX}}) + u_i), \tag{18}$$

where we have used the results in equations (11)-(13). The total concentration of translational resources demanded at module i, which we denote as R^i_{TL}, equals the amount of TIC:

$$R^i_{\mathrm{TL}} = M_i \approx \frac{\varphi_i R_{\mathrm{TX}} R_{\mathrm{TL}} D^t_i}{\delta_i k_{i2} \kappa_i} f_i(u_i, R_{\mathrm{TX}}). \tag{19}$$

2.2 Mathematical model for a module with constitutive expression

For a module $i \in \mathcal{B}$, where transcription initiation does not require the presence of a TA, we assume that the transcriptional resource $\mathrm{R_{TX}}$ can bind directly with the promoter to form the PIC $\mathrm{C_i}$, which initiates transcription and produce mRNA $\mathrm{m_i}$:

$$\mathrm{R_{TX}} + \mathrm{D_i} \underset{\mathrm{k_i^-}}{\overset{\mathrm{k_i^+}}{\rightleftharpoons}} \mathrm{C_i}, \qquad\qquad \mathrm{C_i} \xrightarrow{\varphi_\mathrm{i}} \mathrm{C_i} + \mathrm{m_i}. \tag{20}$$

This is possible due to processes such as native TAs binding to the promoter. The mRNA then demands translational resources to form TIC, which initiates translation to produce the protein $\mathrm{x_i}$ as output. The corresponding chemical reactions are identical to those in (3). Based on mass-action kinetics and the reactions in (20) and (3), ODEs describing the module dynamics are:

$$\frac{\mathrm{d}}{\mathrm{d}t} C_i = k_i^+ D_i R_{\mathrm{TX}} - k_i^- C_i, \tag{21}$$

$$\frac{\mathrm{d}}{\mathrm{d}t} m_i = \varphi_i C_i - \kappa_i^+ m_i R_{\mathrm{TL}} + \kappa_i^- M_i - \delta_i m_i, \tag{22}$$

$$\frac{\mathrm{d}}{\mathrm{d}t} M_i = \kappa_i^+ m_i R_{\mathrm{TL}} - \kappa_i^- M_i - \omega_i M_i, \tag{23}$$

$$\frac{\mathrm{d}}{\mathrm{d}t} x_i = \beta_i M_i - \gamma_i x_i. \tag{24}$$

Assuming that the dynamics of all complexes and mRNA are much slower than that of the protein, as we did in Section 2.1, we obtain their QSS concentrations as:

$$C_i = \frac{R_{\mathrm{TX}} D_i}{k_i}, \qquad m_i = \frac{\varphi_i C_i}{\delta_i + \omega_i \frac{R_{\mathrm{TL}}}{\kappa_i}}, \qquad M_i = \frac{\varphi_i C_i R_{\mathrm{TL}}}{\delta_i \kappa_i + \omega_i R_{\mathrm{TL}}}, \tag{25}$$

where $k_{i2} = k_i^- / k_i^+$. Using conservation of DNA: $D_i^t = D_i + c_i$, we have

$$D_i = \frac{D_i^t}{1 + R_{\mathrm{TX}}/k_{i2}}, \qquad C_i = \frac{D_i^t R_{\mathrm{TX}}/k_{i2}}{1 + R_{\mathrm{TX}}/k_{i2}}. \tag{26}$$

We further assume, as in the case of modules with inputs, that (15) is satisfied, which states that the free amount of translational resources is limited and that ribosome binding shields mRNAs from degradation. Under this assumption, we have

$$m_i \approx \frac{\varphi_i C_i}{\delta_i}, \qquad M_i \approx \frac{\varphi_i C_i R_{\mathrm{TL}}}{\delta_i \kappa_i}. \tag{27}$$

Substituting (27) into (24), the dynamics of module $i \in \mathcal{B}$ can be simplified as

$$\frac{\mathrm{d}}{\mathrm{d}t} x_i \approx \varphi_i \beta_i \frac{R_{\mathrm{TL}} D_i^t}{\delta_i \kappa_i} \cdot \frac{R_{\mathrm{TX}}/k_{i2}}{1 + R_{\mathrm{TX}}/k_{i2}} - \gamma_i x_i. \tag{28}$$

By (26) and (27), the transcriptional and translational resource demands in module i, R^i_{TX} and R^i_{TL}, can be computed as:

$$R^i_{\mathrm{TX}} = C_i = D_i^t \frac{R_{\mathrm{TX}}/k_{i2}}{1 + R_{\mathrm{TX}}/k_{i2}}, \qquad R^i_{\mathrm{TL}} = M_i \approx \frac{\varphi_i C_i R_{\mathrm{TL}}}{\delta_i \kappa_i} = \varphi_i \frac{R_{\mathrm{TL}} D_i^t}{\delta_i \kappa_i} \cdot \frac{R_{\mathrm{TX}}/k_{i2}}{1 + R_{\mathrm{TX}}/k_{i2}}. \tag{29}$$

2.3 Mathematical model for a network with resource limitations

We now consider a genetic network with N modules in a mammalian cell. The network possibly contains modules from both sets $\mathcal{A}$ (*i.e.* with TA input) and $\mathcal{B}$ (*i.e.* constitutive). In this section, we compute the free amount of transcriptional and translational resources in this network setting. We assume that no TA is produced without a target (*i.e.* if $\mathrm{x_i}$ is a TA then $\mathrm{x_i} = \mathrm{u_j}$ for some $j \in \mathcal{A} \cup \mathcal{B}$). In addition, we assume that the total concentrations of transcriptional and translational resources, R^t_{TX} and R^t_{TL}, respectively, are both conserved:

$$\begin{aligned} R^t_{\mathrm{TX}} &= R_{\mathrm{TX}} + \sum_{i=1}^{N} R^i_{\mathrm{TX}} = R_{\mathrm{TX}} \cdot \left[1 + \sum_{i \in \mathcal{A}} \frac{1}{k_{i2}} \Big(u_i + D_i^t f_i(u_i, R_{\mathrm{TX}}) \Big) + \sum_{i \in \mathcal{B}} D_i^t \frac{R_{\mathrm{TX}}/k_{i2}}{1 + R_{\mathrm{TX}}/k_{i2}} \right], \\ R^t_{\mathrm{TL}} &= R_{\mathrm{TL}} + \sum_{i=1}^{N} R^i_{\mathrm{TL}} = R_{\mathrm{TL}} \cdot \left[1 + \sum_{i \in \mathcal{A}} \frac{\varphi_i D_i^t}{\kappa_i \delta_i} \cdot f_i(u_i, R_{\mathrm{TX}}) + \sum_{i \in \mathcal{B}} \frac{\varphi_i D_i^t}{\delta_i \kappa_i k_i} \cdot \frac{R_{\mathrm{TX}}/k_{i2}}{1 + R_{\mathrm{TX}}/K_{i2}} \right], \end{aligned} \tag{30}$$

where we have used the resource demands in each module computed previously in (18), (19) and (29) for the two types of modules. The dynamics of the entire network can therefore be described as follows:

$$\frac{\mathrm{d}}{\mathrm{d}t}x_i = \hat{T}_i R_{\mathrm{TL}} R_{\mathrm{TX}} F_i(u_i, R_{\mathrm{TX}}) - \gamma_i x_i, \tag{31}$$

where

$$\hat{T}_i := \frac{\varphi_i \beta_i D_i^t}{\delta_i \kappa_i k_{i2}}, \qquad F_i(u_i, R_{\mathrm{TX}}) := \begin{cases} f_i(u_i, R_{\mathrm{TX}}) = \dfrac{\left(\frac{u_i}{k_{i1}}\right)^n}{1+\left(\frac{u_i}{k_{i1}}\right)^n \cdot \left(1+\frac{R_{\mathrm{TX}}}{k_{i2}}\right)}, & \forall i \in \mathcal{A}, \\ \dfrac{1}{1+R_{\mathrm{TX}}/k_{i2}}, & \forall i \in \mathcal{B} \end{cases},$$

and R_{TX} and R_{TL} are the solutions to the algebraic equations in (30). To explicitly find the solutions to (30), we consider the following physically relevant parameter regime: (a) for $i \in \mathcal{A}$, $D_i^t \ll u_i$, (b) for $i \in \mathcal{B}$, $D_i^t \ll k_{i2}$, and (c) $\varphi_i D_i^t R_{\mathrm{TX}}^t \ll \kappa_i \delta_i k_{i2}$. Based on assumptions (a)-(b), we have that

$$R_{\mathrm{TX}}^t \approx R_{\mathrm{TX}} \left(1 + \sum_{i \in \mathcal{A}} u_i/k_{i2}\right), \qquad \Rightarrow \qquad R_{\mathrm{TX}} \approx \frac{R_{\mathrm{TX}}^t}{1 + \sum_{i \in \mathcal{A}} u_i/k_{i2}}. \tag{32}$$

These assumptions imply that the major mode of transcriptional resource sequestration is through AD:CoA binding in solution, instead of binding of CoAs on the promoter. This is consistent with our experimental observations in Supplementary Figure 4. From assumption (c), we have $R_{\mathrm{TL}}^t \approx R_{\mathrm{TL}}$, implying that translational resource sharing is negligible, which is also compatible with our experimental observations (see main text). These assumptions allow us to simplify (31) into the following:

$$\frac{\mathrm{d}}{\mathrm{d}t}x_i = T_i R_{\mathrm{TX}} F_i(u_i, R_{\mathrm{TX}}) - \gamma_i x_i, \tag{33}$$

where $T_i := \hat{T}_i R_{\mathrm{TL}}^t$ and $R_{\mathrm{TX}} = R_{\mathrm{TX}}^t/(1 + \sum_{i \in \mathcal{A}}[u_i/k_{i2}])$. At steady state, we have from (33)

$$x_i = \alpha_i \frac{R_{\mathrm{TX}}}{k_{i2}} F_i(u_i, R_{\mathrm{TX}}), \tag{34}$$

where

$$\alpha_i = \frac{\varphi_i \beta_i D_i^t}{\delta_i \kappa_i \gamma_i}, \qquad R_{\mathrm{TX}} = \frac{R_{\mathrm{TX}}^t}{1 + \sum_{i \in \mathcal{A}} u_i/k_{i2}}. \tag{35}$$

2.4 Effects of TA toxicity on gene expression levels

Previous studies have demonstrated that over-expression of strong TAs can cause toxicity in addition to squelching[10–13]. Similarly, we saw a ~2-fold decrease in the concentration of cells measured by flow cytometry when transfected with hEF1a:Gal4-VPR compared to samples transfected with a control plasmid that did not express a protein (Supplementary Figure 4e). This decrease in cell concentration could in part be caused by a reduction in cell growth rate; indeed, we found that Gal4-TAs can reduce expression of Ki-67, a marker for cell division (Supplementary Figure 14). Assuming that initial cell concentrations are identical regardless of Gal4 TA amount and a typical division time of HEK-293FT cells of ~22 hours, a ~2-fold decrease in the concentration of cells in a 48- our 72-hour experiment corresponds to a ~45% or ~30% decrease in growth rate, respectively. Since the dilution rate of all biomolecules are dictated by growth rate, this could potentially lead to an increase in output levels to counteract the negative effect of resource loading described in the previous sections. This section discusses the combined effects of resource loading and loading-induced growth inhibition on gene expression.

To account for the effect of potential growth inhibition due to loading, we first used the experimental data in Supplementary Figure 30b to obtain a phenomenological model that maps Gal4 TA DNA dosage (D_{u}^{t}) to cell growth rate. In this experiment, we had high transfection efficiency, titration of a Gal4 TA (Gal4-VPR), and a record of flow rates during data collection, all of which were needed to obtain reasonably accurate measurements of the change in cell density caused by the TA. Assuming that the initial cell concentration for all Gal4-VPR amounts are identical and, for simplicity, that the change in cell density can be completely attributed to changes in cell growth rate, we map changes in cell density ($\Delta\mathrm{CD} :=$ Fold-Δ cells $\cdot\mu\mathrm{L}^{-}1$) to growth rate (k_{dil}), using the relationship derived from exponential cell growth:

$$k_{\mathrm{dil}}(D_{\mathrm{u}}^{t}) = k_{\mathrm{dil}}(D_{\mathrm{u}}^{t} = 0) + \ln(\Delta\mathrm{CD}(D_{\mathrm{u}}^{t})) \cdot \frac{1}{T}, \tag{36}$$

where $T = 72$ hr is the time we took the measurement. The specific growth rate without Gal4-VPR is set to be 0.0315 hr^{-1}, which corresponds to a doubling time of ~ 22 hrs, matching our experimental observations for HEK-293FT cells. We then fit the estimated growth rate data using a model of the form:

$$k_{\mathrm{dil}}(D_{\mathrm{u}}^{t}) = \frac{a}{1 + D_{\mathrm{u}}^{t}/b} \tag{37}$$

This model is chosen because it captures the fact that (i) ΔCD is a decreasing function of Gal4-VPR amount and (ii) at worst, an extremely high amount of Gal4-VPR would cause the cell to not grow. The parameters a and b are then obtained using least square fitting (Supplementary Figure 15a).

We next combined this phenomenological growth rate model with the resource competition model derived in the

Sections above. Specifically, the protein decay rates γ_i in (33), are now a function of D^t_u:

$$\gamma_i(D^t_\mathrm{u}) = k_{\mathrm{deg},i} + k_\mathrm{dil}(D^t_\mathrm{u}), \tag{38}$$

where k_dil is the dilution rate constant dictated by cell growth and $k_{\mathrm{deg},i}$ is the degradation rate constant of protein i. For a circuit consisting of Gal4 TA (u) and a constitutive output protein (x), the combined model takes the form:

$$\frac{\mathrm{d}}{\mathrm{d}t}u = \tilde{\alpha}_\mathrm{u} D^t_\mathrm{u} \frac{R_\mathrm{TX}/k_\mathrm{u}}{1 + R_\mathrm{TX}/k_\mathrm{u}} - (k_\mathrm{deg,u} + k_\mathrm{dil}(D^t_\mathrm{u}))u, \qquad \frac{\mathrm{d}}{\mathrm{d}t}x = \tilde{\alpha}_\mathrm{x} D^t_\mathrm{x} \frac{R_\mathrm{TX}/k_\mathrm{x}}{1 + R_\mathrm{TX}/k_\mathrm{u}} - (k_\mathrm{deg,x} + k_\mathrm{dil}(D^t_\mathrm{u}))x, \tag{39}$$

where

$$\tilde{\alpha}_i = \frac{\varphi_i \beta_i}{\delta_i \kappa_i}, \qquad R_\mathrm{TX} = \frac{R^t_\mathrm{TX}}{1 + u/k_\mathrm{u2}}, \qquad \text{for } i = \mathrm{u,x},$$

k_u2 is the dissociation constant of Gal4 TA with transcriptional resources, and k_u and k_x are the dissociation constants between transcriptional resources and the promoters of Gal4 TA (u) or the output protein (x), respectively. Based on (39), when the DNA dosage of Gal4 TA (D^t_u) is increased, the amount of Gal4 TA (u) increases and the decay rate constants of both species decreases as a result of (37). The former effect leads to a reduction in free transcriptional resources, reducing the concentration of x, while the latter effect increases the concentration of both u and x in each cell through reduced decay. Hence, qualitatively, after taking loading-induced growth inhibition into account, depending on parameters, the output level x may either increase or decrease as the Gal4 TA DNA amount is increased. In contrast, when the dilution rate does not change with Gal4 TA DNA amount, as in the models in the previous Sections, the output of a constitutive gene can only decrease as Gal4 DNA increases. This qualitative analysis is supported by simulation results showing that increasing the sensitivity of growth rate changes to the dosage of a TA causes an increase in non-target (Output) expression levels at intermediate TA dosage (Supplementary Figure 15b).

We next evaluated other parameters that are important for determining the change in gene expression levels in response to both resource loading and growth rate changes by TAs. In Figure 2, we found that different non-target constitutive promoters are differentially affected by Gal4 TAs. This may reflect differences in the affinity of each promoter for shared transcriptional resources. Simulation results for non-target promoters driving output expression with variable resource affinity (k_x) show that promoters with higher affinity for resources are more positively affected by the growth rate decrease (Supplementary Figure 15c).

The effect of growth inhibition also depends on the degradation rate constants of proteins. This is because the overall decay rate of a protein protein is determined by the sum of its dilution and degradation rates. As shown in equation (38), the relative contributions from these two factors dictates the effect of growth inhibition on output level. Simulations with our model show that the increase in output protein level is most significant when the output protein

is very stable and the TA is very unstable (Supplementary Figure 15d-f). In this parameter regime, output decay is mainly determined by dilution, and hence a decrease in cell growth rate due in response to TAs will lead to a notable increase in output protein level. On the other hand, if the output protein is very unstable and the TA is very stable, then a decrease in growth rate can in fact amplify the knockdown of Output caused by the TA (Supplementary Figure 15f). In our system, it is most likely the case that the Output protein (a fluorescent reporter) is relatively stable[14] whereas the Gal4 TAs, like most transcription factors, are relatively unstable[9,15]. Thus, we expect the loading-induced growth inhibition to cause a relative increase in output expression compared to the absence of growth inhibition.

Overall, our modeling and simulation results suggest that growth inhibition due to toxicity of Gal4 TAs may explain some of the observed increase in Output protein levels in the presence of Gal4 TA, which we further discuss in Section 3.4. For Gal4 TAs that substantially affect growth, the observed decrease in Output levels in Figure 2 is likely smaller than the actual decrease in transcriptional activity due to squelching.

3 Supplementary Note 3: Experimental validation of the resource sharing model

3.1 Identification of the source of resource loading

We first investigated whether the observed resource loading effects in our experiments (*e.g.* Figure 1) could be attributed to transcriptional resources, translational resources, or both. Resource sequestration due to expression of Gal4 TAs can occur at different stages of gene expression: (a) production of Gal4 mRNA/protein molecules requires both transcriptional and translational resources, (b) Gal4-driven activation of target gene(s) causes additional sequestration of both types of resources, and (c) Gal4 directly binds to and sequesters transcriptional resources in solution and/or at off-target DNA loci[16]. We validated that the Gal4 TA variants repress CMV transcription via RT-qPCR measurement of CMV-driven mRNA levels (Supplementary Figure 4a-b). Indeed, CMV-driven mRNA levels were knocked down ~2-fold by Gal4-VP16 and ~16-fold by Gal4-VPR (Supplementary Figure 4b). In the same samples, a fraction of the cells were collected for measurement of protein expression levels via flow cytometry. The magnitude of knockdown of protein levels were similar to that of the mRNA levels (Supplementary Figure 4c), suggesting that most of the knockdown occurred at the transcriptional level. Additional experiments show that CMV expression is knocked down by VPR alone and Gal4-VPR , but not the Gal4 DBD nor the luminescent protein Fluc2 (Supplementary Figure 4d). Because the Gal4 DBD and Fluc2 were expressed by the same promoter (hEF1a) as Gal4-VPR and VPR and thus placed similar demands on gene expression resources, we concluded that of factors (a)-(c) described above, (a) is negligible compared to (b) and (c). Furthermore, knockdown of CMV expression by Gal4-VPR was similar regardless of whether the Gal4-driven promoter was present, indicating that in this system, (b)

is small compared to (c). However, resource sequestration by strong TA-driven promoters has been observed elsewhere[17], indicating that (b) cannot always be ignored. Thus, the AD, whether or not fused to the TA (Gal4), sequesters transcriptional resources from the CMV promoter and is the major player in the observed knockdown of CMV expression.

3.2 Application of the model to a simple 2-module system

To validate that our resource sharing model could recapitulate experimental data, we utilized the 2-module system shown in Figure 1e and described in the main text. In short, the system was comprised of two modules: (i) a constitutive CMV-driven reporter (CMV:Output_1) and (ii) one of the five Gal4 TAs and a Gal4-driven reporter (UAS:Output_2). Each Gal4 TA was titrated to measure the dose-response of both CMV:Output_1 and UAS:Output_2. To estimate Gal4 TA levels in each sample, we utilized a fluorescent reporter (Gal4 Marker) whose plasmid DNA was co-titrated with each Gal4 TA plasmid prior to transfection. In addition to the improved accuracy of estimating Gal4 levels (see Methods and Supplementary Figure 3), the Gal4 Marker is also useful for comparing dose-responses and fits between different analyses of the same data and among experiments.

From the mechanistic model (34), we predicted that the differences between the Gal4 TA variants in terms of their activation, self-squelching, and non-target squelching could be entirely explained by one parameter: k_{i2} (the dissociation constant between CoA resources and either ADs or promoters, see Section 2.1). We thus simultaneously fit the following steady-state equations for CMV:Output_1 and UAS:Output_2 to all Gal4 TA dose-response curves, forcing all parameters except k_{22} (the dissociation constant between each Gal4 TA's AD and CoA resources) to be the same. The equations below are the application of equation (34) to the CMV/UAS 2-module system.

$$\text{Output}_1 = \alpha_1 \cdot \frac{\frac{R_{\text{TX}}}{k_{12}}}{1 + \frac{R_{\text{TX}}}{k_{12}}}, \tag{40}$$

$$\text{Output}_2 = \alpha_2 \cdot \frac{\frac{R_{\text{TX}}}{k_{22}} \cdot \left(\frac{u_2}{k_{21}}\right)^2}{1 + \left(\frac{u_2}{k_{21}}\right)^2 \cdot \left(1 + \frac{R_{\text{TX}}}{k_{22}}\right)}, \tag{41}$$

where $R_{\text{TX}} = R^t_{\text{TX}}/(1 + u_2/k_{22})$. Because Gal4 forms a dimer[18], the cooperativity of Gal4 (u_2)-DNA binding (n) is set to 2.

To evaluate the performance of the model, we looked in particular at the relative shapes of the CMV:Output_1 and UAS:Output_2 dose-response curves, as well as the CMV vs UAS curves (Supplementary Figure 6a-c). From this initial round of fitting, we observed that the fits were able to qualitatively recapitulate the CMV data but not the UAS data. We thus tested several other conditions with different assumptions regarding model reduction and for which parameters are equivalent across all Gal4 TAs. First, we allowed each Gal4 TA to also have a unique α_2 parameter, which would be the case if, for example, the structure of the different fusion proteins or any specific CoAs bound by

the AD of each TA affects the maximum transcription rate of the promoter. This additional degree of freedom substantially improves the model fits, and are the fits shown in Figure 1 and Supplementary Figure 2. Interestingly, activators with smaller fit values of k_{22} also tended to have smaller fit values of α_2, which would suggest that TAs with stronger CoA binding achieve lower maximal transcription initiation rates. Future experimental work will explore the exact relationship between the model parameters and protein/DNA architectures, including this possible connection between k_{22} and α_2. We additionally performed and compared fitting under the following conditions:

(i) Assuming that the AD affects DBD-DNA binding and thus that k_{21} is different across Gal4 TA;

(ii) Assuming that each AD binds to a different specific CoA (or set thereof) that is most-limiting, rather than all to the same global CoA like mediator, and thus that R^t_{TX} is different across Gal4 TA

(iii) Assuming that $k_{i2} \ll R_{\mathrm{TX}}$, such that the R_{TX}/k_{22} terms in the denominator of equations (40)-(41) vanish;

(iv) Assuming both (i) and (iii) above;

(v) Assuming that the degradation rate of each Gal4 TA protein is not identical, and thus that the Gal4 Marker values are scaled by a factor γ_{u} for each Gal4 TA;

(vi) Assuming both (iii) and (v) above.

According to the fitting results in Supplementary Figure 6a-c, the only other set of modeling assumptions that we tested that qualitatively matched the data was (v), for which the fits are highlighted in Supplementary Figure 6d. This assumption is important to consider both in this dataset and in others that rely on TX Markers to quantify relative DNA dosages. The TX Marker is at best only proportional to the amount of DNA delivered, and is not a direct reporter of any protein's level unless they are directly fused. In the case of TAs such as the Gal4 variants used here, it has been shown that stronger activation can induce faster degradation of the TA[15]. In addition, fusions of large disordered regions such as activation domains can lead to instability without improving transcriptional activation[19]. Indeed, immunostaining of HA-tagged Gal4 TAs shows that the relative median expression level of Gal4 TAs varies approximately 4-fold (Supplementary Figure 7a-b. We inverted the γ_{u} values fit in Supplementary Figure 6d and compared their relative values to the relative median expression level of each Gal4 TAs, finding good agreement for each Gal4 TA except Gal4-Rta (Supplementary Figure 7c. However, since the sample size was not very large and Rta was a substantial outlier, we chose not to move forward with this variant of the fitting. An equally likely explanation for the data could simply be that Gal4-p65 has an exceptionally high expression level due to a slow degradation rate.

3.3 Prediction of CMV/UAS expression across DNA dosages using the model

To further validate the model, we took the parameters fit on the median CMV:Output$_1$ and UAS:Output$_2$ levels and used them to predict the entire distribution of CMV:Output$_1$ and UAS:Output$_2$ levels across all cells. In the case of the medians, we used the fluorescent reporter Gal4 Marker to indicate the amount of Gal4 TA delivered to each sample. However, because some samples were transfected with diluted amounts of Gal4-{AD} and Gal4 Marker plasmids, not every transfected cell expressed Gal4 Marker. We thus instead turned to the TX Marker, which is proportional to the copy number of each promoter delivered to each cell, including the output reporters and Gal4 TAs. We adjusted the models for both CMV:Output$_1$ and UAS:Output$_2$ to account for this proportionality by multiplying them by the value of the TX Marker:

$$\text{Output}_1 = (\text{TX Marker}) \cdot \alpha_1 \cdot \frac{\frac{R_{\text{TX}}}{k_{12}}}{1 + \frac{R_{\text{TX}}}{k_{12}}},$$

$$\text{Output}_2 = (\text{TX Marker}) \cdot \alpha_2 \cdot \frac{\frac{R_{\text{TX}}}{k_{22}} \cdot \left(\frac{u_2}{k_{21}}\right)^n}{1 + \left(\frac{u_2}{k_{21}}\right)^n (1 + \frac{R_{\text{TX}}}{k_{22}})},$$

$$R_{\text{TX}} = \frac{R^t_{\text{TX}}}{1 + \frac{u_2}{k_{22}}}.$$

Since the x-values of the fits are in units of ‘Gal4 Marker’ and the x-values of the TX Marker vs Output curves are in the units of ‘TX Marker’, it was then necessary to convert from Gal4 Marker to TX Marker. To do the conversion, we computed the ratio TX Marker:Gal4 Marker in each sample by fitting TX Marker vs Gal4 Marker along the entire distribution of cells to the function (Gal4 Marker) = $u_2 = m \cdot$ (TX Marker), then used the slope (m) of the fit to convert TX Marker values to Gal4 Marker values. We then had these final equations for generating the predicted curves for the full distribution:

$$\text{Output}_1 = (\text{TX Marker}) \cdot \alpha_1 \cdot \frac{\frac{R_{\text{TX}}}{k_{12}}}{1 + \frac{R_{\text{TX}}}{k_{12}}}, \tag{42}$$

$$\text{Output}_2 = (\text{TX Marker}) \cdot \alpha_2 \cdot \frac{\frac{R_{\text{TX}}}{k_{22}} \cdot \left(\frac{m \cdot (\text{TX Marker})}{k_{21}}\right)^2}{1 + \left(\frac{m \cdot (\text{TX Marker})}{k_{21}}\right)^2 \cdot \left(1 + \frac{R_{\text{TX}}}{k_{22}}\right)}, \tag{43}$$

$$R_{\text{TX}} = \frac{R^t_{\text{TX}}}{1 + \frac{m \cdot (\text{TX Marker})}{k_{22}}}. \tag{44}$$

For the predictions, we used the same parameters shown in Figure 1 and Supplementary Figure 2, in which all parameters are equivalent for all Gal4 TAs except α_2 and k_{22}, which were unique for each Gal4 TA. A comparison of the predicted curves and the data are shown in Supplementary Figure 8. The plots demonstrate that the model, which was only fit on the median expression levels of each sample, can effectively capture the salient features of the full

distribution.

3.4 Increases in constitutive promoter expression caused by Gal4-activators

In Figure 2, we noticed that the level of output driven by the hUBC, hMDM2c, and hMDM2 promoters increased in combination with some Gal4 TAs. Here we discuss in detail several possible reasons for these observed increases.

As discussed in Section 2.4, toxicity associated with TAs can reduce cell growth rates, which thereby reduces the dilution rate of all species and can increase protein concentrations. Depending on the relative effect of a Gal4 TA on the dilution and production rates of a gene's output, we predicted that the changes in Output levels and cell density would be either positively correlated (effect on the production rate is stronger) or negatively correlated (effect on the dilution rate is stronger); see Supplementary Figure 16a. While for a given TA, the effect on growth rate is expected to be similar across samples in a given cell line, the effect of resource loading on a given promoter depends on the specific transcriptional resources both loaded by the TA and utilized by the promoter. Thus, the effect of a TA on Output production, but not decay, likely varies per promoter. We therefore evaluated the correlations between changes in cell density (Fold-Δ Cells $\cdot\mu L^{-1}$) and changes in Output level (Fold-Δ {P}:Output_1) in the HEK cell lines (Supplementary Figure 16b-f). We chose to only focus on HEK cells because the other cell lines have relatively low transfection efficiencies, which limits the sensitivity of measuring changes in cell density. Overall, we found that the changes in Output level and cell density were positively correlated for most promoters in both cell lines, with mostly consistent Pearson's correlation coefficients (Supplementary Figure 16e) but variable slopes for fit lines (Supplementary Figure 16f). Interestingly, only one promoter, hUBCs, showed no correlation at all (supplementary Figure 16d-f), suggesting that the effect of the Gal4 TAs on its transcription and the growth rate may perfectly offset. Of the three promoters that increased in response to Gal4 TAs (hUBC, hMDM2c, hMDM2), both hUBC and hMDM2c, but not hMDM2, showed an increase in expression that was clearly negatively correlated with the change in cell density (Supplementary Figure 16d-f). Thus, the data for these two promoters is consistent with a model in which outputs driven by both promoters are minimally affected by resource loading by Gal4 TAs, and increase due to a reduction in cell growth rate due to Gal4 TA toxicity.

However, there are other possible explanations for both the differences in response to resource loading itself and the increase in expression levels. For example, increased output may result from off-target binding and activation by Gal4, offsetting any negative effects of resource loading. Both hUBC and the hMDM2 variants (along with some of the other human promoters we tested) have a high GC content and contain many CGG triplets, which form a critical part of the Gal4 binding sequence (CGG-$N_1$1-CCG)[18]. In the case of hMDM2c and hUBC, we observed increased output levels in the presence of TAs with different DBDs fused to VPR (Supplementary Figure 17), suggesting that non-specific binding is likely not the dominant reason for the increase in their expression levels. Conversely, the

full-length hMDM2 promoter only showed increased output levels in combination with Gal4-VPR and not TAs with different DBDs (Supplementary Figure 17), suggesting that it may in fact be bound and activated by Gal4 TAs. Examining the sequence of hMDM2 (see Source Data for annotated sequence files for all plasmids used in this study), we found two consensus Gal4 binding sites. While hMDM2c also contains one of these binding sites, hMDM2 also contains a second p53-activated promoter not present in hMDM2c that may facilitate efficient transcriptional activation by Gal4 TAs.

Finally, it is possible that the TAs take part in regulatory interactions that are currently unaccounted for in our model. In Vero 2.2 cells, we did not see an increase in hMDM2 output when Gal4 TAs were added, suggesting that if Gal4 TAs can indeed bind and activate the promoter, the cellular context may affect the degree of activation. For example, Gal4 TAs may cooperate with or otherwise affect p53 activation through an unknown mechanism, thereby affecting hMDM2 promoter transcription. In addition, the increases in expression levels could be caused by a reduction in expression of specific transcriptional repressor(s) due to resource loading, thereby relieving repression of these promoters. Further work will be needed to further investigate all the hypotheses presented above.

3.5 A model-driven definition for TA 'strength'

Typically, it is assumed that TAs with 'stronger' ADs will drive higher maximal output expression from on-target genes (see *e.g.* Supplementary Figure 1 and studies comparing dCas9 activators[20,21]). However, in HEK-293FT cells, we found that the maximal output expression levels driven by different Gal4 TAs were highly similar (within ~10% for all except Gal4-Rta, which gave ~2-fold lower max output – see Supplementary Figures 2 & 3). Similarly, recent work where the same AD was fused to different ZFP DBDs showed a similar effect, with the maximum ZFP-driven expression levels being within a 2-fold range[17]. In addition, either tuning the affinity of a ZFP's DBD-DNA interaction or tuning the number of ZFP binding sites in the output promoter significantly affected the maximum expression driven by the ZFP when fused to the weaker ADs VP16 and VP16, but not when fused to VPR[17]. However, as the ZFP vs output curves were still increasing at the measured dosages, it is possible that at high enough dosages of the ZFPs, the actual maximum expression would be similar among ZFP-VP16/VP64 mutants. These results thus suggest the need for better quantification of the 'strength' of TAs, as current methods may miss important behavior.

In our model, ignoring the contribution of resource loading (*i.e.* assuming $R_{\mathrm{TX}} = R^t_{\mathrm{TX}}$), we can see that when the promoter is saturated with u_i and R_{TX}, the steady state of Output_1 reduces to α_i:

$$\mathrm{Output}_i = \alpha_i \cdot \frac{\frac{R_{\mathrm{TX}}}{k_{i2}} \cdot \left(\frac{u_i}{k_{i1}}\right)^n}{1 + \left(\frac{u_i^n}{k_{i1}}\right)^n (1 + \frac{R_{\mathrm{TX}}}{k_{i2}})}, \qquad \left(\frac{u_i}{k_{i1}}\right)^n, \frac{R_{\mathrm{TX}}}{k_{i2}} \gg 1 \qquad \Rightarrow \qquad \mathrm{Output}_i \approx \alpha_i.$$

Thus, the rate of transcription from a promoter is not just dependent on the affinity of the TA's AD for resources (k_{i2}), but also on the maximal transcription rate of the promoter (α_i). To provide a useful definition of TA 'strength', we considered the sensitivity of the transcription rate to small amounts of TA. In particular, we re-write (34) as

$$\text{Output}_i = \alpha_i \cdot \frac{R_{\text{TX}}}{k_{i2}} \cdot \frac{\left(\frac{u_i}{k_{i1}}\right)^n}{1 + \left(\frac{u_i}{K_{\text{eff}}}\right)^n},$$

where

$$K_{\text{eff}} = \frac{k_{i1}}{\left(1 + \frac{R_{\text{TX}}}{k_{i2}}\right)^{1/n}}.$$

Here, K_{eff} approximates the amount of u_i needed for half of the promoters of Output_i to be bound by R_{TX}. This quantity is affected by both the strength of u_i's DBD-DNA binding (k_{i1}) and its AD-R_{TX} binding (k_{i2}). Thus, the strength of a TA depends both on its DNA binding strength as well as its affinity for transcriptional resources. For TAs with high affinity for R_{TX} (*i.e.* $k_{i2} \ll R_{\text{TX}}$), the above equations further reduce to:

$$K_{\text{eff}} = k_{i1}\left(\frac{k_{i2}}{R_{\text{TX}}}\right)^{1/n} \qquad \text{Output}_i = \alpha_i \cdot \frac{\left(\frac{u_i}{K_{\text{eff}}}\right)^n}{1 + \left(\frac{u_i}{K_{\text{eff}}}\right)^n}, \tag{45}$$

which resembles the familiar Hill function for TAs typically used to model their behavior. However, this model is only applicable at low values of u_i, where u_i does not significantly load the free amount of R_{TX}. In this regime, we can further reduce the model by assuming that $u_i \ll K_{\text{eff}}$:

$$\text{Output}_i = \alpha_i \cdot \left(\frac{u_i}{K_{\text{eff}}}\right)^n \tag{46}$$

Thus, at low inputs of u_i, Output_i expression is inversely proportional to the n-th power of K_{eff} of u_i. We can thus use K_{eff} as a measure for the sensitivity of Output_i transcription to low levels of u_i, which we believe to be a good proxy for the strength of a TA. For TAs that bind with no cooperativity (n = 1), $1/K_{\text{eff}}$ is the slope of the linear regime of the u_i vs Output_i curve.

4 Supplementary Note 4: Response of lentiviral-integrated promoters to resource loading

Previously, Natesan *et al.* demonstrated that genomically-integrated UAS promoters did not experience self-squelching by high dosages of either Gal4-VP16 or Gal4-p65[22]. However, more recent results have suggested that genomic promoters can indeed be affected by depletion of CoA resources[23,24]. Thus, we sought to determine whether the gene knockdown seen in our transfection experiments and our model of resource sharing also hold for promoters in the genomic context.

4.1 Non-target TRE promoter

We first measured the effect of Gal4 TA resource sequestration on a non-target gene integrated into the genome. A challenge of measuring such an effect for constitutive promoters (*e.g.* CMV) is that if the method of delivering the resource competitor is transient (*e.g.* via transfection), then the competitor may be diluted out of the cell before a significant change to a constitutively expressed gene can be observed. Alternatively, stable transfection or integration adds weeks to the experiment and may cause additional undesirable effects (such as toxicity) resulting from stable expression of a strong resource competitor. To circumvent this issue, we instead tested the effect of Gal4 TAs on the Tet-On system (Supplementary Figure 9a). First, a lentiviral construct containing two transcription units, hEF1a:rtTA and TRE:Output, was integrated into HEK-293FT cells, yielding a cell line we call HEK-293FT-TetOn. After validating the infection and allowing the cells to grow for two weeks, we co-transfected the cells with different Gal4 TAs and a TX Marker. TX Marker indicates the dosage of Gal4 TA received by each cell. 24 hours after transfection of the GAl4 TAs, we induced rtTA activation by addition of 1 μg $\cdot$ mL^{-1} Dox; 48 hours after adding Dox, data was collected by flow cytometry. The delay in Dox addition allowed for Gal4 levels to build up before expression of the TRE:Output reporter, increasing the sensitivity of the measurement to any effect by the Gal4 TAs on TRE:Output expression.

We first performed a simple analysis of gated populations of cells. Samples transfected with Gal4 TAs had a smaller percentage of cells that were TRE:Output$^+$/TX Marker$^+$ than the sample transfected with the non-resource-loading plasmid Gal4-None (Supplementary Figure 9b). While we postulated that the decrease in the double-positive population likely results from reduction in TRE:Output expression to below the detectable limit, it is also possible that cells with high levels of both Gal4 TA and rtTA experience cytotoxicity, yielding fewer cells to be measured in the double-positive gate.

To measure TRE:Output as a function of Gal4 TA levels, we binned the transfected HEK-293-TetOn cells for different amounts of TX Marker, then computed the median level of TRE:Output in each bin. To exclude from the

analysis cells that either silenced their TetOn integration or were not transduced, we took two approaches in parallel: (i) gate all cells expressing TRE:Output above the threshold (as drawn in Supplementary Figure 9b) or (ii) estimate the percentage (N) of cells expressing the lenti construct and gate the top N% of TRE:Output-expressing cells. For (ii), we measured N as ~25% based on the percent of cells expressing TRE:Output above the threshold in untransfected cells. In either case, TRE:Output decreases as a function of Gal4 TA levels (Supplementary Figure 9c), with Gal4 TAs that strongly knocked down CMV expression also strongly knocking down TRE:Output (see Figure 1 for reference). The difference in the gating methods manifests at the higher dosages of the Gal4 TAs. The concentration-based threshold likely under-estimates the degree of knockdown by Gal4 TAs as some cells may become undetectable and thus no longer pass the gate. Conversely, the percentile-based binning method likely over-estimates the degree of knockdown since some cells that disappear from the double-positive population may in fact be lost due to toxicity rather than knockdown below the threshold. Note that lentiviral transgenes typically integrate into transcriptionally-active loci, potentially enhancing or reducing their susceptibility to resource loading compared to a transgene that is either integrated at a uniformly random position or into a specific loci.

We then examined whether the model fit from the transient transfection CMV knockdown data (Figure 1e-f) could predict the knockdown of genomically-integrated TRE:Output. Because the CMV and TRE promoters naturally have different transcription rates and different DNA copy numbers in the transfection and lentiviral contexts, the α_1 parameter was scaled based on the ratio of CMV:Output$_1$ in HEK-293FT cells transfected with 0 ng Gal4-{AD} to TRE:Output in untransfected, Dox$^+$ HEK-293FT-TetOn cells. In the transient data, Gal4 Marker is used to approximate the amount of Gal4 TA delivered; in the integrated data, TX Marker serves the same purpose. However, because the relative amounts of the Gal4 Marker and TX Marker plasmids co-transfected with the Gal4 TAs were not the same between experiments, we normalized the values of Gal4 Marker to TX Marker. In the transient data, the Gal4 Marker was at a 2:5 ratio (in terms of DNA mass) to the Gal4 TAs; in the integrated data, the TX Marker was at a 1:1 ratio. Both marker plasmids were nearly identical in size, making DNA mass a good approximation for the number of plasmids. Thus, the x-values (Gal4 Marker for the transient data, TX Marker for the integrated data) input to the model were scaled by the ratio $\frac{2}{5}$ to account for the difference. Besides the α_i parameters and the x-values, all other parameters and aspects of the model were kept the same. Overall, the model was able to accurately predict the knockdown of TRE:Output as a function of the Gal4 DNA dosage (Supplementary Figure 9c). When comparing to the concentration-thresholded TRE:Output medians, the model predicts a slightly higher sensitivity to Gal4 levels than was observed. This discrepancy may result from stronger resource binding affinity (*i.e.* smaller k_{i2} parameter) for the genomically integrated TRE promoter compared to the episomal CMV promoter, or from under-estimation of knockdown by the concentration threshold method as described above. Comparing to the percentile-gated TRE:Output medians, the model more accurately predicts the sensitivity of TRE:Output to Gal4 TAs, but under-estimates the sharpness of the decrease in TRE:Output expression. Overall, the effects of resource loading on

episomal CMV expression predicts with reasonable accuracy the response of the lenti-integrated Tet-On system, indicating that the model and fit parameters apply to multiple contexts for promoter localization in the cell.

4.2 On-target UAS promoter

We next tested on-target activation of a genomically-integrated gene by Gal4 TAs to determine if self-squelching occurs for integrated genes (Supplementary Figure 10a). First, we infected HEK-293FT cells with a lentivirus containing the transgene UAS:Output, yielding a cell line we call HEK-293FT-UAS. After validating the infection and allowing the cells to grow for two weeks, we co-transfected the cells with different Gal4 TAs and a TX Marker) to mark the dosage of Gal4 TA received by each cell. In this transfection experiment, we replicated the conditions from the Gal4 dose-response experiment shown in Figure 1 & Supplementary Figures 2-3 in order to compare the transient and integrated UAS responses to Gal4 TAs directly; the only major difference was that the UAS promoter was integrated into the genome rather than co-transfected with the other plasmids and the CMV promoter was not included. As with the transient experiment, we also co-titrated a Gal4 Marker plasmid with each Gal4-{AD} prior to transfection. Cell fluorescence was measured by flow cytometry at 72 hours post-transfection.

In each sample, we measured the dose-response curves as a function of Gal4 levels using the same process as with the integrated TetOn system: cells were binned based on TX Marker levels and the median level of UAS:Output was calculated for the transduced cells in each bin. Based on the maximum percent of cells that were UAS:Output$^+$ across all bins for all Gal4 TA variants (85-95%), we estimated the percent of transduced cells at 90%. Using this information, we gated and computed the median of the top 90% of cells by UAS:Output in each bin. Gating based just on UAS:Output$^+$ cells (analogous to the threshold-gating strategy with the integrated TetOn system) strongly over-estimates UAS:Output at low Gal4 levels, and thus was avoided. From the measured bin medians and distribution of fluorescence per cell in the samples, we can clearly see self-squelching of UAS:Output at higher dosages of Gal4 TAs (Supplementary Figure 10b).

We next evaluated whether the model fits to the transient UAS Gal4 dose-response data predict the response of integrated UAS promoters to the same Gal4 TAs. Like before with the transient data, we used the ratio of Gal4 Marker:TX Marker to more precisely estimate the relative amount of the Gal4 TAs in samples transfected with varying amounts of the Gal4 TA plasmids (see Section 3.2). Overall, the fits from the episomal median UAS expression data well-predicted the response of the integrated UAS:Output, though with a relatively high degree of noise as measured by the normalized root-mean-square error, CV(RMSE) (Supplementary Figure 10b). The high noise may result from variance in the copy number of the integrated UAS promoter per cell, which is uncorrelated with that of the Gal4 TA because of their different mechanisms and times of delivery to the cells. Though in most samples the predicted values trace out the observed distribution quite well, we can notice several instances where the

measured maximum expression level was higher than predicted. A likely cause for this discrepancy is the difference in the copy number of UAS promoters integrated vs transiently transfected into the cells. In addition, it is possible that expression of the UAS promoter is enhanced or suppressed when located in the genome instead of an episomal plasmid. The copy number of the UAS promoter affects the α_i parameter in the reduced resource sharing model, which for saturating TA inputs scales the output expression level. At high dosages of transfected Gal4 TAs, the integrated UAS:Output appears to decrease more strongly than predicted in response to increasing Gal4 TA levels. This increased sensitivity could result from high concentrations of the Gal4 TAs also suppressing expression of the TX Marker, such that the DNA dosage vs TX Marker relationship becomes non-linear. Alternatively, like in the case of percentile-binning with the integrated Tet-On system, the 90th %ile thresholding of UAS:Output expression per bin could underestimate the median if UAS:Output^{+}/TX Marker^{+} cells experience higher toxicity and thus are eliminated from the population. Finally, the model well-predicted the shift in the dose-response curves when decreasing the dosage of Gal4 TA plasmids for each variant except Gal4-p65. The reduced predictability of Gal4-p65 may result from the relatively high expression level of the Gal4-p65 protein compared to other Gal4 TAs (Supplementary Figure 7).

The lack of self-squelching observed by Natesan *et al.* may be explained by their use of a bulk reporter (SEAP) to measure Gal4-driven expression. Since the effects of resource loading depend on the concentration of resource competitors, bulk assays could miss results that are clear at the single-cell level. From our dose-response curves (Figure 1) and model of resource sharing (Supplementary Note 2), we can reason that increasing the relative amount of TA plasmid in a transfection mixture causes the optimal dosage of TA plasmid for maximal activation to be delivered to cells with lower overall DNA dosages. Since co-transfected plasmids are correlated in their delivery to cells[25], this will result in the optimal concentration of the TA being delivered to cells with fewer reporter plasmids. Thus, above a critical amount of TA, increasing TA dosage reduces expression of the output regardless of whether it is measured in bulk or at the single-cell level. Conversely, if the reporter and activator amounts are independent (*e.g.* when the reporter has been integrated into the genome prior to transfection of the TA as done by Natesan *et al.*[22] and replicated by us in Supplementary Figure 10), then titrating the TA shifts which transfected cells produce high amounts of output, but the overall output in bulk is unchanged until very high activator dosages are delivered. Indeed, when we directly compare measurements of the entire transfected distribution, the integrated UAS promoter appears to decrease less at high Gal4 TA levels than the transiently-transfected UAS promoter (Supplementary Figure 10c). Thus, even though with the single-cell data we can clearly see a strong self-squelching effect by the Gal4 TAs, this effect is not as visible with bulk measurements of the entire distribution of transfected cells.

5 Supplementary Note 5: Model of the endoRNase-based iFFL

5.1 Model formulation

Here we develop a mathematical model for the endoRNase-based incoherent feedforward loop (iFFL) described in Figure 4a of the main text. The circuit in Figure 4a consists of two modules: (i) an iFFL-regulated module (Module 1) that includes expression of an endoRNase and the output whose mRNA contains a targeting site for the endoRNase and (ii) a disturbance generating module (Module 2) that produces a Gal4 TA. Module 2 is called a disturbance generating module because it demands resources through (1) transcription and translation of the Gal4 TA and (2) the Gal4 TA sequestering transcriptional resources in solution. These reactions affect the availability of both transcriptional and translational resources available to Module 1, which contains the output protein whose concentration needs to be regulated. More specifically, the larger the amount of Module 2 transfected, the smaller the amount of free resources available to Module 1.

In the following, we describe a mechanistic mathematical model for the regulated module. In particular, the mRNA of the output protein m_y and the endoRNase m_x are both transcribed from basal promoters (D_y and D_x, respectively) to form PICs (c_y and c_x, respectively) with CoAs R_{TX}. The mRNAs bind with translational resource R_{TL} to form TICs (M_y and M_x, respectively), which then allow translation of the mRNAs to produce the output protein y and the endoRNase x. These reactions are similar to ones we derived for an unregulated gene in (3) and (20):

$$R_{TX} + D_i \underset{k_i^-}{\overset{k_i^+}{\rightleftharpoons}} C_i, \qquad C_i \xrightarrow{\varphi_i} C_i + m_i, \qquad m_i + R_{TL} \underset{\kappa_i^-}{\overset{\kappa_i^+}{\rightleftharpoons}} M_i, \qquad M_i \xrightarrow{\beta_i} M_i + i, \qquad m_i \xrightarrow{\delta_i} \varnothing, \qquad i \xrightarrow{\gamma_i} \varnothing, \tag{47}$$

where $i \in \{x, y\}$. The endoRNase can bind with the mRNA of the output (m_y) to form a complex B. The endoRNase then enzymatically cleavages m_y before it dissociates. We assume that once the endoRNase binds with the mRNA m_y, translational resource can no longer bind with it. These reactions are modeled as follows:

$$x + m_y \underset{d}{\overset{a}{\rightleftharpoons}} B, \qquad\qquad B \xrightarrow{\theta} x. \tag{48}$$

By mass-action kinetics, the reactions in (47)-(48) can be modeled by the following ODEs:

$$\frac{\mathrm{d}}{\mathrm{d}t}C_\mathrm{y} = k_\mathrm{y}^+ R_\mathrm{TX} D_\mathrm{y} - k_\mathrm{y}^- C_\mathrm{y}, \tag{49}$$

$$\frac{\mathrm{d}}{\mathrm{d}t}C_\mathrm{x} = k_\mathrm{x}^+ R_\mathrm{TX} D_\mathrm{x} - k_\mathrm{x}^- C_\mathrm{x}, \tag{50}$$

$$\frac{\mathrm{d}}{\mathrm{d}t}m_\mathrm{y} = \varphi_\mathrm{y} C_\mathrm{y} - \kappa_\mathrm{y}^+ m_\mathrm{y} R_\mathrm{TL} + \kappa_\mathrm{y}^- M_\mathrm{y} - \delta_\mathrm{y} m_\mathrm{y} - axm_\mathrm{y} + dB, \tag{51}$$

$$\frac{\mathrm{d}}{\mathrm{d}t}m_\mathrm{x} = \varphi_\mathrm{x} C_\mathrm{x} - \kappa_\mathrm{x}^+ m_\mathrm{x} R_\mathrm{TL} + \kappa_\mathrm{x}^- M_\mathrm{x} - \delta_\mathrm{x} m_\mathrm{x}, \tag{52}$$

$$\frac{\mathrm{d}}{\mathrm{d}t}M_\mathrm{y} = \kappa_\mathrm{y}^+ m_\mathrm{y} R_\mathrm{TL} - \kappa_\mathrm{y}^- M_\mathrm{y}, \tag{53}$$

$$\frac{\mathrm{d}}{\mathrm{d}t}M_\mathrm{x} = \kappa_\mathrm{x}^+ m_\mathrm{x} R_\mathrm{TL} - \kappa_\mathrm{x}^- M_\mathrm{x}, \tag{54}$$

$$\frac{\mathrm{d}}{\mathrm{d}t}y = \beta_\mathrm{y} M_\mathrm{y} - \gamma_\mathrm{y} y, \tag{55}$$

$$\frac{\mathrm{d}}{\mathrm{d}t}x = \beta_\mathrm{x} M_\mathrm{x} - \gamma_\mathrm{x} x - axm_\mathrm{y} + dB + \theta B, \tag{56}$$

$$\frac{\mathrm{d}}{\mathrm{d}t}B = axm_\mathrm{y} - dB - \theta B. \tag{57}$$

The steady state solution of (49)-(57) can be computed as follows:

$$C_\mathrm{x} = \frac{R_\mathrm{TX} D_\mathrm{x}}{k_\mathrm{x}}, \qquad C_\mathrm{y} = \frac{R_\mathrm{TX} D_\mathrm{y}}{k_\mathrm{y}}, \qquad B = \frac{xm_\mathrm{y}}{K_\mathrm{M}}, \tag{58}$$

$$M_\mathrm{y} = \frac{R_\mathrm{TL} m_\mathrm{y}}{\kappa_\mathrm{y}}, \qquad M_\mathrm{x} = \frac{R_\mathrm{TL} m_\mathrm{x}}{\kappa_\mathrm{x}}, \qquad m_\mathrm{y} = \frac{\varphi_\mathrm{y} C_\mathrm{y}}{\delta_\mathrm{y} + \theta x / K_\mathrm{M}}, \tag{59}$$

$$m_\mathrm{x} = \frac{\varphi_\mathrm{x} C_\mathrm{x}}{\delta_\mathrm{x}}, \qquad y = \frac{\beta_\mathrm{y} M_\mathrm{y}}{\gamma_\mathrm{y}}, \qquad x = \frac{\beta_\mathrm{x} M_\mathrm{x}}{\gamma_\mathrm{x}}, \tag{60}$$

where we have defined the following lumped parameters:

$$k_i = \frac{k_i^-}{k_i^+}, \qquad \kappa_i = \frac{\kappa_i^-}{\kappa_i^+}, \qquad K_\mathrm{M} = \frac{d+\theta}{a}, \qquad i \in \{\mathrm{x,y}\}. \tag{61}$$

Parameter k_i represents the dissociation constant between the constitutive promoter of the output/endoRNase with CoAs, parameter κ_i is the dissociation constant between the mRNA of the output/endoRNase with translational resources, and K_M is the Michaelis-Menten constant quantifying the enzymatic capacity of the endoRNase, with a smaller K_M indicating stronger affinity of the endoRNase binding with its mRNA target and/or slower cleavage. From (58)-(60), we can compute the steady state concentration of the output protein as:

$$y = \frac{\varphi_\mathrm{y}\beta_\mathrm{y} R_\mathrm{TX} R_\mathrm{TL} D_\mathrm{y}}{\gamma_\mathrm{y} k_\mathrm{y} \kappa_\mathrm{y} \delta_\mathrm{y}} \left(1 + \frac{\theta x}{\delta_\mathrm{y} K_\mathrm{M}}\right)^{-1} = \frac{\varphi_\mathrm{y}\beta_\mathrm{y} R_\mathrm{TX} R_\mathrm{TL} D_\mathrm{y}}{\gamma_\mathrm{y} k_\mathrm{y} \kappa_\mathrm{y} \delta_\mathrm{y}} \left(1 + \frac{\varphi_\mathrm{x}\beta_\mathrm{x}\theta R_\mathrm{TX} R_\mathrm{TL} D_\mathrm{x}}{\gamma_\mathrm{x} k_\mathrm{x} \kappa_\mathrm{x} \delta_\mathrm{x} \delta_\mathrm{y} K_\mathrm{M}}\right)^{-1}. \tag{62}$$

In our iFFL module, the output protein and the endoRNase are transcribed from the same DNA plasmid with equal

promoters (see Figure 4a). We therefore set $k := k_{\mathrm{x}} = k_{\mathrm{y}}$ and write conservation of DNA concentration as

$$D^t = D_{\mathrm{y}} + C_{\mathrm{y}} = D_{\mathrm{x}} + C_{\mathrm{x}}, \tag{63}$$

where D^t is the total concentration of the DNA plasmid, which we assume to be a time-invariant parameter. In practice, in a transient transfection experiment, the concentration of DNA plasmid in a single cell decreases slowly over time as cell volume grows and as the cell divides. Since these dynamics happen at a much slower timescale than those described in (49)-(57), we can still use the analytical results obtained here to guide the design of the iFFL module. Nevertheless, in Section 5.3, we perform numerical simulations of the model (49)-(57) taking slow dilution of DNA plasmid into account. From these simulations, we found that the qualitative results obtained assuming no DNA dilution are still largely valid, especially when comparing the output from iFFL modules with different parameters at the same time point. Therefore, we compute here the free concentrations of DNAs D_{x} and D_{y} as follows:

$$D := D_{\mathrm{y}} = \frac{D^t}{1 + R_{\mathrm{TX}}/k} = D_{\mathrm{x}} = \frac{D^t}{1 + R_{\mathrm{TX}}/k}. \tag{64}$$

By defining $R := R_{\mathrm{TX}} \cdot R_{\mathrm{TL}}$, we can re-write (62) as

$$y = \frac{\varphi_{\mathrm{y}}\beta_{\mathrm{y}}RD}{\gamma_{\mathrm{y}}k\kappa_{\mathrm{y}}\delta_{\mathrm{y}}}\left(1 + \theta \cdot \frac{\varphi_{\mathrm{x}}\beta_{\mathrm{x}}RD}{\gamma_{\mathrm{x}}k\kappa_{\mathrm{x}}\delta_{\mathrm{x}}\delta_{\mathrm{y}}K_{\mathrm{M}}}\right)^{-1}. \tag{65}$$

By introducing the lumped parameters:

$$V_{\mathrm{y}} := \frac{\varphi_{\mathrm{y}}\beta_{\mathrm{y}}}{\gamma_{\mathrm{y}}k\kappa_{\mathrm{y}}\delta_{\mathrm{y}}}, \qquad \text{and} \qquad \epsilon := \frac{\gamma_{\mathrm{x}}k\delta_{\mathrm{x}}\delta_{\mathrm{y}}K_{\mathrm{M}}}{\varphi_{\mathrm{x}}\beta_{\mathrm{x}}\theta} \cdot \kappa_{\mathrm{x}}, \tag{66}$$

(equivalent to equation (103)) we conclude the derivation of equation (102) in the main text:

$$y = V_{\mathrm{y}} \cdot \frac{D \cdot R}{1 + D \cdot R/\epsilon}. \tag{67}$$

To experimentally quantify the iFFL module's robustness, we use the fluorescence output from a co-transfected TX Marker (z) to indirectly measure the availability of resources and the number of DNA plasmids transfected into a given cell ($D \cdot R$). In our experimental setup, we include a TX Marker that is driven by the identical promoter that drives expression of x and y. Thus, it uses the same pool of transcriptional resources $\mathrm{R_{TX}}$ for transcription. We

therefore model its expression through the following chemical reactions:

$$\mathrm{R_{TX}} + \mathrm{D_z} \underset{\mathrm{k_z^-}}{\overset{\mathrm{k_z^+}}{\rightleftharpoons}} \mathrm{C_z}, \quad \mathrm{C_z} \xrightarrow{\varphi_z} \mathrm{C_z} + \mathrm{m_z}, \quad \mathrm{m_z} + \mathrm{R_{TL}} \underset{\kappa_z^-}{\overset{\kappa_z^+}{\rightleftharpoons}} \mathrm{M_z}, \quad \mathrm{M_z} \xrightarrow{\beta_z} \mathrm{M_z} + \mathrm{z}, \quad \mathrm{m_z} \xrightarrow{\delta_z} \varnothing, \quad \mathrm{z} \xrightarrow{\gamma_z} \varnothing, \tag{68}$$

which give rise to the following ODE model based on mass-action kinetics:

$$\frac{\mathrm{d}}{\mathrm{d}t} C_z = k_z^+ R_{TX} D_z - k_z^- C_z, \tag{69}$$

$$\frac{\mathrm{d}}{\mathrm{d}t} m_z = \varphi_z C_z - \kappa_z^+ m_z R_{TL} + \kappa_z^- M_z - \delta_z m_z, \tag{70}$$

$$\frac{\mathrm{d}}{\mathrm{d}t} M_z = \kappa_z^+ m_z R_{TL} - \kappa_z^- M_z, \tag{71}$$

$$\frac{\mathrm{d}}{\mathrm{d}t} z = \beta_z M_z - \gamma_z z. \tag{72}$$

Setting (69)-(72) to steady state, we obtain that

$$C_z = \frac{R_{TX} D_z}{k_z}, \qquad m_z = \frac{\varphi_z C_z}{\delta_z} = \frac{\varphi_z R_{TX} D_z}{k_z \delta_z}, \qquad M_z = \frac{\varphi_z R_{TX} R_{TL} D_z}{\kappa_z k_z \delta_z}, \qquad z = \frac{\beta_z M_z}{\gamma_z} = \frac{\varphi_z \beta_z R_{TX} R_{TL} D_z}{\kappa_z k_z \delta_z \gamma_z}. \tag{73}$$

Since the DNA plasmid encoding the TX Marker is co-transfected with the one that encodes the iFFL module, we assume that $D_z^t = \rho D^t$, where ρ is a positive constant. Applying conservation of DNA concentration: $D_z^t = D_z + C_z$, we have

$$D_z = \frac{D_z^t}{1 + R_{TX}/k_z} = \frac{D_z^t}{1 + R_{TX}/k} = \frac{\rho D^t}{1 + R_{TX}/k} = \rho D, \tag{74}$$

where the second equality arises from the fact that x, y, and z are driven by equal promoters. Hence, the steady state concentration of the TX Marker can be written as:

$$z = \frac{\rho \varphi_z \beta_z R D}{\kappa_z k \delta_z \gamma_z},$$

where we have substituted into (73) the result in (74) and utilized the fact that $k_z = k$. By defining

$$V_z := \frac{\rho \varphi_z \beta_z}{\kappa_z k \delta_z \gamma_z}, \tag{75}$$

we have that $z = V_z \cdot D \cdot R$. When combined with equation (102) in the main text, this allows us to derive equation (104) in the main text:

$$y = V_y \cdot \epsilon \cdot \frac{z/(V_z \cdot \epsilon)}{1 + z/(V_z \cdot \epsilon)}.$$

Based on the above model, two experimentally-measureable performance metrics of an iFFL module can be quantified as follows:

$$Y_{\max} = V_{\mathrm{y}} \cdot \epsilon, \qquad \text{and} \qquad Z_{50} = V_{\mathrm{z}} \cdot \epsilon, \tag{76}$$

where $Y_{\max}$ is the maximum output from the iFFL module with abundant resources and high DNA plasmid copy number, and Z_{50} is the TX Marker's fluorescence level at which the iFFL module's output is half of $Y_{\max}$. Z_{50} also serves as an inverse measure of the iFFL's robustness

5.2 Detailed comparison of miRNA- and endoRNase-based iFFL models

In this section, using mathematical models, we compare the endoRNase-based iFFL proposed in this work and the miRNA-based iFFL, which has been previously described in[26,27]. To this end, we first establish a mathematical model for a miRNA-based iFFL-regulated module and quantify how model parameters affect its robustness performance.

miRNA-based iFFL model

In a miRNA-based iFFL-regulated module, the mRNA of the output protein $\mathrm{m_y}$ and the miRNA $\mathrm{m_x}$ are both transcribed from basal promoters ($\mathrm{D_y}$ and $\mathrm{D_x}$, respectively) to form PICs ($\mathrm{c_y}$ and $\mathrm{c_x}$, respectively) with CoAs $\mathrm{R_{TX}}$. The mRNA bind with translational resource $\mathrm{R_{TL}}$ to form TICs ($\mathrm{M_y}$), which then allow translation of the mRNA to produce the output protein y. These reactions are similar to ones we derived for an endoRNase-regulated module in (47):

$$\mathrm{R_{TX}} + \mathrm{D_i} \underset{\mathrm{k_i^-}}{\overset{\mathrm{k_i^+}}{\rightleftharpoons}} \mathrm{C_i}, \quad \mathrm{C_i} \xrightarrow{\varphi_{\mathrm{i}}} \mathrm{C_i} + \mathrm{m_i}, \quad \mathrm{m_y} + \mathrm{R_{TL}} \underset{\kappa_{\mathrm{y}}^-}{\overset{\kappa_{\mathrm{y}}^+}{\rightleftharpoons}} \mathrm{M_y}, \quad \mathrm{M_y} \xrightarrow{\beta_{\mathrm{y}}} \mathrm{M_y} + \mathrm{y}, \quad \mathrm{m_i} \xrightarrow{\delta_{\mathrm{i}}} \varnothing, \quad \mathrm{y} \xrightarrow{\gamma_{\mathrm{y}}} \varnothing, \tag{77}$$

where $\mathrm{i} \in \{\mathrm{x}, \mathrm{y}\}$. The miRNA can bind with the RNA-induced silencing complex (RISC), U, to form a miRNA-RISC complex Q. The complex Q can then bind with mRNA $\mathrm{m_y}$ to form a miRNA-RISC-mRNA complex B to cleavage the mRNA at a rate constant θ. We assume that once the miRNA-RISC complex R binds with the mRNA $\mathrm{m_y}$, translational resource can no longer bind with it. These reactions are modeled as follows:

$$\mathrm{m_x} + \mathrm{U} \underset{\mathrm{d_1}}{\overset{\mathrm{a_1}}{\rightleftharpoons}} \mathrm{Q}, \qquad \mathrm{Q} + \mathrm{m_y} \underset{\mathrm{d_2}}{\overset{\mathrm{a_2}}{\rightleftharpoons}} \mathrm{B}, \qquad \mathrm{B} \xrightarrow{\theta} \mathrm{Q}. \tag{78}$$

By mass-action kinetics, the reactions in (77)-(78) can be modeled by the following ODEs:

$$\frac{\mathrm{d}}{\mathrm{d}t}C_\mathrm{y} = k_\mathrm{y}^+ R_\mathrm{TX} D_\mathrm{y} - k_\mathrm{y}^- C_\mathrm{y}, \tag{79}$$

$$\frac{\mathrm{d}}{\mathrm{d}t}C_\mathrm{x} = k_\mathrm{x}^+ R_\mathrm{TX} D_\mathrm{x} - k_\mathrm{x}^- C_\mathrm{x}, \tag{80}$$

$$\frac{\mathrm{d}}{\mathrm{d}t}m_\mathrm{y} = \varphi_\mathrm{y} C_\mathrm{y} - \kappa_\mathrm{y}^+ m_\mathrm{y} R_\mathrm{TL} + \kappa_\mathrm{y}^- M_\mathrm{y} - \delta_\mathrm{y} m_\mathrm{y} - a_2 Q m_\mathrm{y} + d_2 B, \tag{81}$$

$$\frac{\mathrm{d}}{\mathrm{d}t}m_\mathrm{x} = \varphi_\mathrm{x} C_\mathrm{x} - \kappa_\mathrm{x}^+ m_\mathrm{x} R_\mathrm{TL} + \kappa_\mathrm{x}^- M_\mathrm{x} - \delta_\mathrm{x} m_\mathrm{x} - a_1 R m_\mathrm{x} + d_1 Q, \tag{82}$$

$$\frac{\mathrm{d}}{\mathrm{d}t}M_\mathrm{y} = \kappa_\mathrm{y}^+ m_\mathrm{y} R_\mathrm{TL} - \kappa_\mathrm{y}^- M_\mathrm{y}, \tag{83}$$

$$\frac{\mathrm{d}}{\mathrm{d}t}y = \beta_\mathrm{y} M_\mathrm{y} - \gamma_\mathrm{y} y, \tag{84}$$

$$\frac{\mathrm{d}}{\mathrm{d}t}Q = a_1 m_\mathrm{x} U - d_1 Q - a_2 Q m_\mathrm{y} + d_2 B + \theta B, \tag{85}$$

$$\frac{\mathrm{d}}{\mathrm{d}t}B = a_2 Q m_\mathrm{y} - d_2 B - \theta B. \tag{86}$$

The steady state solution of (79)-(86) can be computed as follows:

$$C_\mathrm{x} = \frac{R_\mathrm{TX} D_\mathrm{x}}{k_\mathrm{x}}, \qquad C_\mathrm{y} = \frac{R_\mathrm{TX} D_\mathrm{y}}{k_\mathrm{y}}, \qquad B = \frac{Q m_\mathrm{y}}{K_\mathrm{M}}, \tag{87}$$

$$M_\mathrm{y} = \frac{R_\mathrm{TL} m_\mathrm{y}}{\kappa_\mathrm{y}}, \qquad m_\mathrm{y} = \frac{\varphi_\mathrm{y} C_\mathrm{y}}{\delta_\mathrm{y} + \theta Q / K_1}, \qquad Q = \frac{m_\mathrm{x} U}{K_2}, \tag{88}$$

$$m_\mathrm{x} = \frac{\varphi_\mathrm{x} C_\mathrm{x}}{\delta_\mathrm{x}}, \qquad y = \frac{\beta_\mathrm{y} M_\mathrm{y}}{\gamma_\mathrm{y}}, \tag{89}$$

where we have defined the following lumped parameters:

$$k_i = \frac{k_i^-}{k_i^+}, \qquad \kappa_i = \frac{\kappa_i^-}{\kappa_i^+}, \qquad K_1 = \frac{d}{a} \qquad K_2 = \frac{d+\theta}{a}, \qquad i \in \{\mathrm{x,y}\}. \tag{90}$$

The physical interpretations of k_i and κ_i are identical to those defined for the endoRNase-based iFFL in (61). Parameter K_1 is dissociation constant describe the binding of miRNA with RISC complex, and K_2 is the Michaelis-Menten constant quantifying the miRNA-RISC complex's enzymatic capability, with a small K_2 indicating stronger affinity of the miRNA-RISC binding with its mRNA target and/or slower cleavage. From (87)-(89), we can compute the steady state concentration of the output protein as:

$$y = \frac{\varphi_\mathrm{y} \beta_\mathrm{y} R_\mathrm{TX} R_\mathrm{TL} D_\mathrm{y}}{\gamma_\mathrm{y} k_\mathrm{y} \kappa_\mathrm{y} \delta_\mathrm{y}} \left(1 + \frac{\theta Q}{\delta_\mathrm{y} K_1}\right)^{-1} = \frac{\varphi_\mathrm{y} \beta_\mathrm{y} R_\mathrm{TX} R_\mathrm{TL} D_\mathrm{y}}{\gamma_\mathrm{y} k_\mathrm{y} \kappa_\mathrm{y} \delta_\mathrm{y}} \left(1 + \frac{\varphi_\mathrm{x} \theta R_\mathrm{TX} U D_\mathrm{x}}{k_\mathrm{x} \delta_\mathrm{x} \delta_\mathrm{y} K_1 K_2}\right)^{-1}. \tag{91}$$

The miRNA and the output mRNA are transcribed from the same DNA plasmid with equal promoter. This promoter is also used for transcription in the endoRNase-based iFFL. Hence, we set $k_\mathrm{x} = k_\mathrm{y} = k$, $D_\mathrm{x} = D_\mathrm{y} = D$ according to

(64), and re-write (91) as

$$y = \frac{\varphi_y \beta_y R_{TX} R_{TL} D_y}{\gamma_y k \kappa_y \delta_y} \left(1 + \frac{\varphi_x \theta R_{TX} U D_x}{k \delta_x \delta_y K_1 K_2}\right)^{-1} = V_y \cdot \frac{D R_{TX} R_{TL}}{1 + D R_{TX}/\epsilon_{miR}}, \tag{92}$$

where V_y is as defined in (66) and

$$\epsilon_{miR} := \frac{k \delta_x \delta_y K_1 K_2}{\varphi_x U \theta}. \tag{93}$$

Similar to the endoRNase-based iFFL, from (92), we can compute:

$$y_{max} = V_y \cdot R_{TL} \cdot \epsilon_{miR}, \qquad \text{and} \qquad Z_{50} = V_z \cdot R_{TL} \cdot \epsilon_{miR}. \tag{94}$$

As we discussed in the main text, these two performance metrics each characterize the output level and the robustness of the miRNA-based iFFL module. Next, we compare these performance metrics of the miR-based iFFL module against those of the endoRNase-based iFFL module.

Comparison between endoRNase- and miRNA-based iFFLs

There are similarities and differences between endoRNase- and miRNA-based iFFL designs. In particular, while the model predicts that both can adapt to changes in transcriptional resources, the same cannot be guaranteed for translational resources for the miRNA-based iFFL. This is because the adapted output level Y_{max} is a function of translational resource R_{TL} for the miRNA-based iFFL (see equation (94)), while it is independent of R_{TL} for the endoRNase-based iFFL (see equation (76)).

From an engineering perspective, tuning the performance of the endoRNase-based iFFL by changing ϵ_{ERN} is more convenient than changing that of the miRNA-based iFFL via ϵ_{miR}. For miRNA, the structure of miRNA flanking regions, hairpins, and sequences can be altered to tune processing efficiencies[28] and cleavage efficiencies[29,30], but these sequence-based tuning mechanisms are not always predictive of function. With proteins, we can easily and predictably tune their translation rates with upstream open reading frames (uORFs)[31] as we have shown in this study, and degradation rates with inducible degradation domains[32–34].

Additionally, our order-of-magnitude parameter estimates suggest that the endoRNase design may be more robust than the miRNA design with respect to perturbations in R_{TX} and D. Recall that we define the robustness metric Z_{50}, which is a measure of an iFFL's robustness to $(R_{TX} R_{TL} D)$. By comparing Z_{50} in (76) and (94), and based on our order-of-magnitude estimates for the involved biochemical parameters, we find that Z_{50}^{ERN} is likely to be smaller than

Z_{50}^{miR}. Specifically, writing these robustness metrics in terms of biochemical parameters, we have:

$$Z_{50}^{\mathrm{ERN}} = V_{\mathrm{z}}^{\mathrm{ERN}} \frac{\gamma_{\mathrm{x}}^{\mathrm{ERN}} k^{\mathrm{ERN}} \delta_{\mathrm{x}}^{\mathrm{ERN}} \delta_{\mathrm{y}}^{\mathrm{ERN}} K_{\mathrm{M}} \kappa_{\mathrm{x}}^{\mathrm{ERN}}}{\varphi_{\mathrm{x}}^{\mathrm{ERN}} \beta_{\mathrm{x}}^{\mathrm{ERN}} \theta_{\mathrm{ERN}}} = \left(\frac{V_{\mathrm{z}}^{\mathrm{ERN}} k^{\mathrm{ERN}} \delta_{\mathrm{x}}^{\mathrm{ERN}} \delta_{\mathrm{y}}^{\mathrm{ERN}}}{\varphi_{\mathrm{x}}^{\mathrm{ERN}}} \right) \cdot \Theta_{\mathrm{ERN}}, \tag{95}$$

$$Z_{50}^{\mathrm{miR}} = V_{\mathrm{z}}^{\mathrm{miR}} R_{\mathrm{TL}} \frac{k^{\mathrm{miR}} \delta_{\mathrm{x}}^{\mathrm{miR}} \delta_{\mathrm{y}}^{\mathrm{miR}} K_1 K_2}{\varphi_{\mathrm{x}}^{\mathrm{miR}} U \theta_{\mathrm{miR}}} = \left(\frac{V_{\mathrm{z}}^{\mathrm{miR}} k^{\mathrm{miR}} \delta_{\mathrm{x}}^{\mathrm{miR}} \delta_{\mathrm{y}}^{\mathrm{miR}}}{\varphi_{\mathrm{x}}^{\mathrm{miR}}} \right) \cdot \Theta_{\mathrm{miR}}, \tag{96}$$

where

$$\Theta_{\mathrm{ERN}} := \frac{\gamma_{\mathrm{x}}^{\mathrm{ERN}} K_{\mathrm{M}} \kappa_{\mathrm{x}}^{\mathrm{ERN}}}{\beta_{\mathrm{x}}^{\mathrm{ERN}} \theta_{\mathrm{ERN}}}, \qquad \Theta_{\mathrm{miR}} := \frac{K_1 K_2 R_{\mathrm{TL}}}{U \theta_{\mathrm{miR}}}.$$

Comparing Z_{50}'s for the two designs is equivalent to comparing the magnitudes of the two lumped parameters Θ_{ERN} and Θ_{miR}. This is because output protein expression does not change with the controller design (*i.e.* endoRNase- or miRNA-based). The promoters driving output expression, and hence the expressions of either the endoRNase or miRNA, are identical in our physical realizations (hEF1a). Thus, we assume that $\varphi_{\mathrm{x}}^{\mathrm{ERN}} = \varphi_{\mathrm{x}}^{\mathrm{miR}}$ and that promoter strength $k^{\mathrm{ERN}} = k^{\mathrm{miR}}$. By the same reasoning, the decay rate constants of the output mRNA are also identical: $\delta_{\mathrm{y}}^{\mathrm{ERN}} = \delta_{\mathrm{y}}^{\mathrm{miR}}$. The half-lives of miRNAs are between 5-20 hours[35] and those of mRNAs are between 5-30 hours[9]. Hence, it is reasonable to assume that $\delta_{\mathrm{x}}^{\mathrm{ERN}} \approx \delta_{\mathrm{x}}^{\mathrm{miR}}$. If we further choose the same TX marker to measure Z_{50} in the two designs, then $V_{\mathrm{z}}^{\mathrm{ERN}} = V_{\mathrm{z}}^{\mathrm{miR}}$. In summary, with reference to (95), the magnitude of $\left(\frac{V_{\mathrm{z}} k \delta_{\mathrm{x}} \delta_{\mathrm{y}}}{\varphi_{\mathrm{x}}} \right)$ is largely independent of the controller design. On the other hand, the difference between Θ_{ERN} and Θ_{miR} is more likely to induce a difference in Z_{50}^{ERN} and Z_{50}^{miR}. Specifically, the design with the smaller Θ will have a smaller Z_{50} and therefore be more robust to changes in $(R_{\mathrm{TX}} D)$. To facilitate this comparison, we define:

$$\Theta_{\mathrm{rel}} := \frac{\Theta_{\mathrm{ERN}}}{\Theta_{\mathrm{miR}}} = \underbrace{\left(\frac{\theta_{\mathrm{miR}}}{\theta_{\mathrm{ERN}}} \right)}_{\Theta_1} \cdot \underbrace{\left(\frac{\gamma_{\mathrm{x}}^{\mathrm{ERN}} \kappa_{\mathrm{x}}^{\mathrm{ERN}}}{R_{\mathrm{TL}} \beta_{\mathrm{x}}^{\mathrm{ERN}}} \right)}_{\Theta_2} \cdot \underbrace{\left(\frac{U K_{\mathrm{M}}}{K_1 K_2} \right)}_{\Theta_3}, \tag{97}$$

where Θ_i are all dimensionless constants. To provide an order-of-magnitude estimate of Θ_{rel}, we determine order-of-magnitude estimates for all Θ_i's.

1. Parameter Θ_1 quantifies the ratio of catalytic rate constants of the two enzymes. The catalytic rate constant θ_{miR} of the let-7 family of miRNAs is ~0.007 s^{-1} (see Haley *et al.*[36]) and that of Cse3, a close analog to CasE, is ~0.08 s^{-1} (see Sashital *et al.*[37]). Hence, we estimate $\Theta_1 \sim 0.1$.

2. Parameter Θ_2 is the ratio of endoRNase mRNAs per protein. This is because at steady state, we have

$$\beta_{\mathrm{x}}^{\mathrm{ERN}} \frac{m_{\mathrm{x}} R_{\mathrm{TL}}}{\kappa_{\mathrm{x}}^{\mathrm{ERN}}} - \gamma_{\mathrm{x}}^{\mathrm{ERN}} x = 0, \qquad \Rightarrow \qquad \Theta_2 = \frac{\gamma_{\mathrm{x}}^{\mathrm{ERN}} \kappa_{\mathrm{x}}^{\mathrm{ERN}}}{R_{\mathrm{TL}} \beta_{\mathrm{x}}^{\mathrm{ERN}}} = \frac{m_{\mathrm{x}}}{x}.$$

In mammalian cells, on average, there are ~3000 proteins per mRNA transcript[9]. However, the transcript per protein ratio varies for different proteins depending on their decay rate constants γ_x. While the decay rate constant for CasE has not been directly measured, based on Section 5.3, we estimate that CasE decays relatively fast; we thus estimate its half-life in HEK-293FT cells to be between 1-5 hours. In comparison, the half-life of a stable protein is similar to the doubling time of the cell, which is around 24 hours. Therefore, we estimate that the protein decay rate constant of CasE can be as many as 20 times larger than that of a stable protein. Hence, the transcript per protein ratio may be as many as 20 times larger than the average number reported in the literature. We thus estimate that $\Theta_2 \sim 20/3000 \approx 0.007$.

3. Parameter Θ_3 is the ratio between the Michaelis-Menten constants of the two enzymes. Prior experiments have measured the Michaelis-Menten constant for the let-7 family miRNAs to be ~8 nM[36]. This measurement lumps the binding reaction of miRNA with RISC and the enzymatic reaction of miRNA-RISC complex binding and cleaving mRNA. Hence, we take $K_{\mathrm{M}}^{\mathrm{miR,eff}} := K_1 K_2 / U \sim 8$ nM. The K_{M} for CasE has not been experimentally measured. However, the dissociation constant $K_D = d/a$ of binding between Cse3 and mRNA was measured to be ~3 nM *in vitro*[37]. Assuming that the association rate constant a of Cse3-mRNA binding is similar to other RNA binding proteins such as L7Ae and MS2[38] ($\sim 10^5$ M^{-1}s^{-1}), the dissociation rate constant (d) for Cse3-mRNA binding is ~1 hr^{-1}. We thus estimate

$$K_{\mathrm{M}} = \frac{d + \theta_{\mathrm{ERN}}}{a} \approx \frac{1/3600\mathrm{s}^{-1} + 0.08s^{-1}}{10^{-4}\mathrm{nM}^{-1}\mathrm{s}^{-1}} = 800\mathrm{nM}. \tag{98}$$

Therefore, we have $\Theta_3 = K_{\mathrm{M}}/K_{\mathrm{M}}^{\mathrm{miR,eff}} \sim 100$.

The above parametric analysis indicates that $\Theta_{\mathrm{rel}} = 0.1 \times 0.007 \times 100 \sim 0.1$ and hence $\Theta_{\mathrm{miR}} \sim 0.1\Theta_{\mathrm{ERN}}$. Although several order-of-magnitude estimates above for binding constants are taken from *in vitro* measurements, this analysis implies that it is likely that $Z_{50}^{\mathrm{ERN}} < Z_{50}^{\mathrm{miR}}$, indicating that the endoRNase-based iFFL is more robust (*i.e.* it can adapt to a larger change in R_{TX} and D). Our experiments with endoRNAse- and miRNA-based iFFLs provides additional support for the above parametric analysis. Specifically, the measured Z_{50} values for endoRNase-based iFFLs (with 0-2 uORFs) are ~10x smaller than that of miRNA-based iFFL (Supplementary Figure 40c). The robustness (Z_{50}) of the miRNA-based iFFL is comparable to that of the endoRNase-based iFFL with 4x uORFs in the 5' untranslated region (UTR) of CasE; 4x uORFs reduce the translation rate of CasE by ~27-fold (Figure 3, supporting our order-of-magnitude estimate for the relative values of Θ_{miR} and Θ_{ERN}.

Finally, as discussed by Bleris *et al.*[26], enzymatic reactions of miRNAs depend on RISC. In addition, miRNA-specific biosynthetic pathways which may be limiting[39,40], such that the magnitude of U in (97) can be reduced when the mRNA concentration is high, leading to an even smaller Θ_{rel}. In contrast, Cas6-family endoRNases

are produced like a normal protein and are independent of secondary factors for cleavage of target RNAs.

5.3 Dynamics of the iFFL in the presence of DNA plasmid dilution

In the Sections above, we derived a mathematical model of the iFFL module assuming that the total concentrations of DNA species $D^t = D^t_{\mathrm{x}} = D^t_{\mathrm{y}}$ and D^t_{z} are constants (*i.e.*, time-invariant). In transiently transfections, however, DNA plasmids are diluted as cell volume grows and as cells divide. Here we take dilution of DNA plasmid into account by including the following DNA plasmid dilution dynamics into our model:

$$\frac{\mathrm{d}}{\mathrm{d}t}D^t = -\gamma D^t, \qquad \frac{\mathrm{d}}{\mathrm{d}t}D^t_{\mathrm{z}} = -\gamma D^t_{\mathrm{z}}, \tag{99}$$

where γ is the dilution rate constant that is proportional to the specific growth rate of the cell. Equation (99) implies that the DNA concentrations follow the temporal dynamics: $D^t(t) = D^t(0)\exp(-\gamma t)$ and $D^t_{\mathrm{z}}(t) = D^t_{\mathrm{z}}(0)\exp(-\gamma t)$. We then simulate (49)-(57) with the added terms for plasmid dilution in (99). The simulation results are shown in Supplementary Figure 40a. From the simulations, we found that increasing the degradation rate of the endoRNase (γ_{x}) reduces Δh, defined as the absolute difference in iFFL output expression from the highest to lowest point in the simulated time-courses, normalized by the output at the final time point. This is intuitive, as a relatively fast degradation rate of the repressive species in an iFFL allows it to reach a quasi-steady-state much faster than the output species, such that there is no delay in the build-up of the repressor. Since increasing γ_{x} decreases the concentration of the protein, it must be offset by an increased production rate (φ_{x}) in order to maintain a given iFFL output level. Our simulations show that combining fast degradation with strong production minimizes Δh (Supplementary Figure 39b). This can be explained by the following iFFL mechanism, which attenuates slowly time-varying DNA concentration as a disturbance input.

Within a given cell, a transient transfection is a step-increase in plasmid DNA followed by a slow decay of plasmid concentration. Thus, there is an initial burst of expression following the uptake of DNA before plasmid dilution reduces RNA and protein production. However, since dilution is much slower than the other reactions involved in the iFFL, we can treat total DNA copy number as if it were fixed at a given time. As long as $z \gg Z_{50}$, the output level will still be sufficiently close to $Y_{\max}$. After many hours of DNA concentration dilution and once z becomes smaller than Z_{50}, then the output will be affected and there is no guarantee that the output will stay close to $Y_{\max}$. This explains the non-monotonic temporal responses of the iFFL regulated genes. For an iFFL with increasing CasE production, the magnitude of Z_{50} decreases, which allows the output level to stay close to $Y_{\max}$ for a longer period of time. In our experimental measurements of the CasE iFFL dynamics, we saw that (i) increasing ϵ by adding additional uORFs to the 5' UTR of CasE increased the accumulated change in iFFL output over time, (ii) the output of all CasE iFFL variants was more stable over time than the miR-FF4 iFFL (Figure 6e), and (iii) between 48-120

hours post-transfection, both Y_{max} and Z_{50} fit to time course data for the CasE iFFL remain largely unchanged while for the miR-FF4 iFFL they decrease 5-10-fold (Figure 40c). Directly comparing miR-FF4 and the 2x-uORFs-CasE iFFLs, we can see that even though the final output levels at 120 hours are equivalent, the maximum output level of the miR-FF4 iFFL is approximately twice as large. Result (i) indicates that the expected relationship between plasmid dilution, z and Z_{50} is experimentally validated. Results (ii) and (iii) together suggest that the degradation rate of the CasE is likely much faster than that of the miRNA. The median half-life of mammalian native miRNAs is ~20 hours[35], indicating that most miRNAs are relatively stable and have a similar degradation rate to the rate of cell division/plasmid DNA dilution. A fast degradation rate of CasE would allow its levels to rapidly reach a quasi-steady-state in reference to the amount of plasmid DNA, thereby reducing the time-delay in iFFL repression action.

Overall, these modeling and experimental results illustrate that our endoRNase-based iFFL enables accurate gene expression control even with slow time-varying input disturbances.

5.4 Effect of growth inhibition by TAs on iFFL performance metrics

In addition to the iFFL design which uses CMVi promoters (see Figures 4 & 5 in the main text), we also tested a variant with hEF1a promoters for robustness to resource loading (Supplementary Figures 25-29). While the hEF1a iFFL output was highly robust to resource loading by Gal4 TAs in HeLa, CHO-K1, Vero 2.2, and U2OS cells, its output modestly increased in response to resource loading in the HEK cell lines (Supplementary Figures 27-29). A similar slight increase in response to resource loading can be seen in the same cell lines in iFFL designs using the CMVi promoter (Figure 22-Supplementary Figure 24). Here we provide a model that explains these observed output increase using the growth inhibition model developed in Section 2.4.

Examining our Gal4-VPR dose-response experiments (Figure 4 & Supplementary Figure 25), we found that the cell concentration was reduced as a function of Gal4-VPR across all samples (Supplementary Figure 30a-b). Notably, the decrease in cell concentration was stronger for the hEF1a iFFL than for the CMVi iFFL. Combining this information with the result that the hEF1a iFFL output increased more in response to resource loading supports the hypothesis that, similar to the case with unregulated genes, toxicity may also be tied to the loading-induced increase in iFFL output expression. Indeed, while we observed strong positive correlations between changes in Output level and cell density for the unregulated (UR) devices and iFFL variants with large numbers of uORFs (and thus larger values of the key parameter ϵ), we saw negative correlations for the iFFL variants with few or no uORFs (Supplementary Figure 30c-d). In particular, the correlations between changes in cell density and increases in output were higher for the iFFL variants with hEF1a promoters rather than CMVi. Since hEF1a is generally less affected by resource loading than CMVi (Figure 2) and the changes in cell density were larger with the hEF1a iFFL, these data

suggest that changes in cell growth rate can also affect iFFL performance.

To evaluate if changes in cell growth rate as a result of toxicity by TAs (Supplementary Figure 14) can increase the output level of the iFFL devices, we examined the iFFL model. Indeed, the two key performance metrics of the iFFL, Y_{max} and Z_{50} can both change with cell growth rate. In particular, the analytical expressions of Y_{max} and Z_{50} are:

$$Z_{50} = \frac{\rho\delta_{\mathrm{y}}}{\kappa_{\mathrm{z}}} \cdot \frac{\varphi_{\mathrm{z}}\beta_{\mathrm{z}}}{\varphi_{\mathrm{x}}\beta_{\mathrm{x}}} \cdot \frac{\delta_{\mathrm{x}}\gamma_{\mathrm{x}}}{\delta_{\mathrm{z}}\gamma_{\mathrm{z}}} \cdot \frac{K_{\mathrm{M}}}{\theta}, \qquad Y_{\mathrm{max}} = \frac{1}{\kappa_{\mathrm{y}}} \cdot \frac{\varphi_{\mathrm{y}}\beta_{\mathrm{y}}}{\varphi_{\mathrm{x}}\beta_{\mathrm{x}}} \cdot \frac{\delta_{\mathrm{x}}\gamma_{\mathrm{x}}}{\gamma_{\mathrm{y}}} \cdot \frac{K_{\mathrm{M}}}{\theta}. \tag{100}$$

Some but not all of the parameters involved in (100) may change in value in response to Gal4 TAs. Since the endoRNase, output, and TX Marker all used the same promoter, 5' UTR, and Kozak sequence, we assume that $\varphi_{\mathrm{x}} = \varphi_{\mathrm{y}} = \varphi_{\mathrm{z}}$ and $\beta_{\mathrm{x}} = \beta_{\mathrm{y}} = \beta_{\mathrm{z}}$. The catalytic efficiency of the endoRNase ($\frac{\theta}{K_{\mathrm{M}}}$), the affinities of the reporter mRNAs for the ribosome (κ_{y} & κ_{z}), and the ratio of TX Marker plasmids to iFFL plasmids (ρ) are also unlikely to be affected by Gal4-VPR either via direct action or through its resource sequestration mechanism. mRNAs are typically relatively unstable compared to proteins[9], so we assume that growth inhibition by Gal4 has a negligible effect on the mRNA decay rate constants δ_{x} and δ_{y}. Based on the above assumptions, the iFFL performance metrics can be written as

$$Z_{50} \propto \frac{\gamma_{\mathrm{x}}(D_{\mathrm{u}}^{t})}{\gamma_{\mathrm{z}}(D_{\mathrm{u}}^{t})} = \frac{k_{\mathrm{deg,x}} + k_{\mathrm{dil}}(D_{\mathrm{u}}^{t})}{k_{\mathrm{deg,z}} + k_{\mathrm{dil}}(D_{\mathrm{u}}^{t})}, \qquad Y_{\mathrm{max}} \propto \frac{\gamma_{\mathrm{x}}(D_{\mathrm{u}}^{t})}{\gamma_{\mathrm{y}}(D_{\mathrm{u}}^{t})} = \frac{k_{\mathrm{deg,x}} + k_{\mathrm{dil}}(D_{\mathrm{u}}^{t})}{k_{\mathrm{deg,y}} + k_{\mathrm{dil}}(D_{\mathrm{u}}^{t})}, \tag{101}$$

where D_{u}^{t} is the dosage of a Gal4 TA that affects cell growth.

Both z and y are fluorescent proteins, which are typically stable and have half-lives over 24 hours[14]. Our measurements of the dynamics of the iFFL suggest that CasE is relatively unstable (see Section 5.3). If the degradation rate of a protein is fast ($k_{\mathrm{deg}} \gg k_{\mathrm{dil}}$), then $\gamma_{\mathrm{x}} \approx k_{\mathrm{deg,x}}$. Thus, a reduction in cell growth rate would reduce the decay rate of a stable protein but insignificantly affect that of an unstable protein. If we assume that CasE is unstable, then equation (101) can be approximated by:

$$Z_{50} \propto \frac{\gamma_{\mathrm{x}}}{\gamma_{\mathrm{z}}} \approx \frac{k_{\mathrm{deg,x}}}{k_{\mathrm{deg,z}} + k_{\mathrm{dil}}}, \qquad Y_{\mathrm{max}} \propto \frac{\gamma_{\mathrm{x}}}{\gamma_{\mathrm{y}}} \approx \frac{k_{\mathrm{deg,x}}}{k_{\mathrm{deg,y}} + k_{\mathrm{dil}}}.$$

Therefore, any decrease in k_{dil} would cause both Y_{max} and Z_{50} to increase, consistent with our experimental observations (Supplementary Figure 31).

These analytical results are also supported by simulations showing that the level of output from an iFFL device increases in response to resource loading and growth inhibition by TAs only when the degradation rate of the output is less than that of the endoRNase (Supplementary Figure 32a-c). In addition, if the degradation rates of both the endoRNase and output proteins are both sufficiently large, then there is no effect on either decay rate and subsequently no effect on the iFFL output level (Supplementary Figure 32c). Thus, the negative correlations between

iFFL output and cell density changes in response to Gal4-VPR in Supplementary Figure 30c-d can likely be attributed to a relatively slow degradation rate of the fluorescent reporter compared to CasE (Supplementary figure 32d). This analysis provides further evidence that CasE is relatively unstable, consistent with our analysis of the iFFL dynamics (see Section 5.3). The positive correlations observed for iFFL variants with more uORFs and thus smaller ϵ can be explained by the iFFL reverting to be more like a UR device such that the relative effect of TAs on growth rates and production rates matter for determining the sign of the correlation (Supplementary Figures 15-16). To prevent changes in growth rate from affecting the performance metrics of an iFFL, it is thus necessary to make all intermediate and output species have identical decay rates, or reduce the ability for resource loading to affect cell growth rate.

5.5 Alleviation of growth inhibition with a less toxic transfection reagent

The growth inhibition effect induced by loading may be alleviated by using a less toxic transfection reagent. In prior iFFL performance testing experiments, we had used either Lipofectamine 3000 or Lipofectamine LTX for transfection of HEK-293 and HEK-293FT cells. These reagents can themselves be toxic, and thus we speculated that the combination of TA overexpression and a toxic reagent could cause excessive toxicity in our samples, ultimately leading to growth inhibition and subsequently the observed increase in iFFL output. To test this hypothesis, we again measured the dose-response of Gal4-VPR on hEF1a iFFL and UR device outputs, this time transfecting cells with Viafect, a less toxic transfection reagent than Lipofectamine 3000 and Lipofectamine LTX. Our results show an elimination of both the decrease in cell concentration and increase in iFFL output as a function of Gal4-VPR (Supplementary Figure 33). Note that the effect of resource loading on the expression level of the UR systems is similar between this experiment and our previous one with Lipofectamine 3000 (Supplementary Figure 25), indicating that the difference in toxicity primarily was a factor for the iFFL rather than the UR device. These data thus support our proposed model for how toxicity by Gal4 TAs induces growth rate changes that then increase iFFL output expression.

6 Supplementary Figures

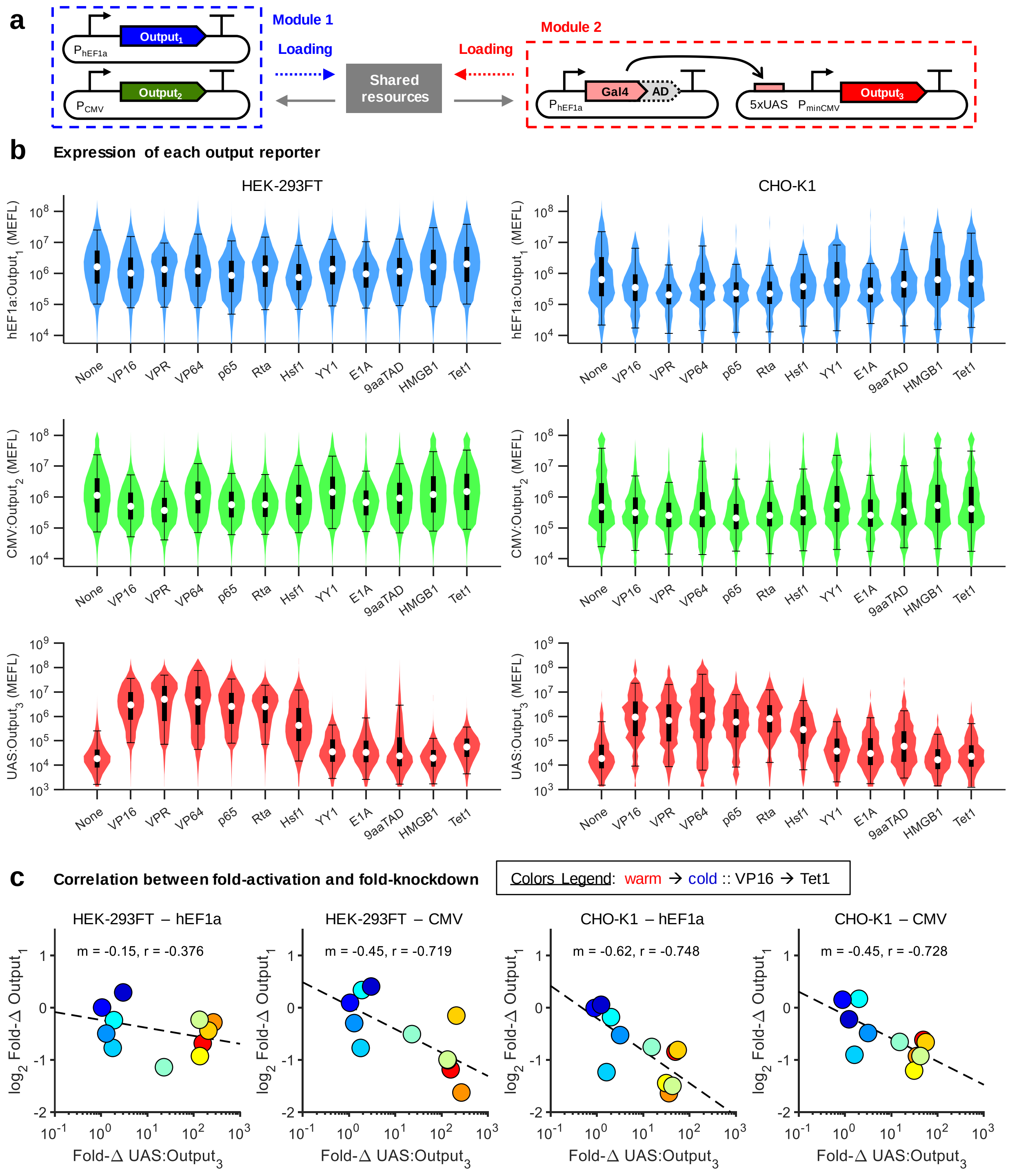

Supplementary Figure 1: Comparison of Gal4 transcriptional activators.

Supplementary Figure 1: (Previous page.) **(a)** Experimental model system to test the on-target activation and non-target resource competition effects of various Gal4 transcriptional activators (TAs). The Gal4 DNA binding domain (DBD) was fused to one of several activation domains (ADs). Gal4-None indicates Gal4 DBD alone, not fused to any AD. The Gal4 TAs and reporters ($Output_1$: mNeonGreen, $Output_2$: mKO2, $Output_3$: TagBFP) were transfected into both HEK-293FT and CHO-K1 cells. **(b)** Violin plots showing the distribution of each reporter in transfected cells. The inset box plots show the median (white dot), 25th to 75th percentiles (thick box), and 5th to 95th percentiles (errorbars). Transfected cells were determined as such for each reporter: hEF1a:$Output_1$: positive for either $Output_1$ or $Output_2$. CMV:$Output_2$: positive for either $Output_1$ or $Output_2$. UAS:$Output_3$: positive for any Output. VPR is a strong AD comprised of VP64, NF-κB p65, and Epstein-Barr Virus Rta[20]. VP16 is from HSV-1 and VP64 is a 4x repeat of the minimal activation domain of VP16. Hsf1, YY1, HMGB1, and Tet1 are the human proteins. E1A is from human adenovirus 5. 9aaTAD is a novel synthetic AD comprised of several tandem 9 amino acid trans-activation domains (9aaTADs[41,42]). **(c)** Correlation between fold-changes (Fold-Δs) of UAS:$Output_3$ and either hEF1a:$Output_1$ or CMV:$Output_2$. The color of each dot ranges from red (warm) to dark blue (cold), tracking the Gal4 TAs from left-to-right as shown in Panel (b), starting with VP16. The Fold-Δs are computed by dividing the median $Output_i$ level in a sample with Gal4-{AD} by that in the sample with Gal4-None. Fold-Δs were computed independently for HEK and CHO cells. m: slope, r: Pearson's correlation. Data was collected 48 hours after transfection. Median values and the number of cells plotted per violin are provided in Source Data.

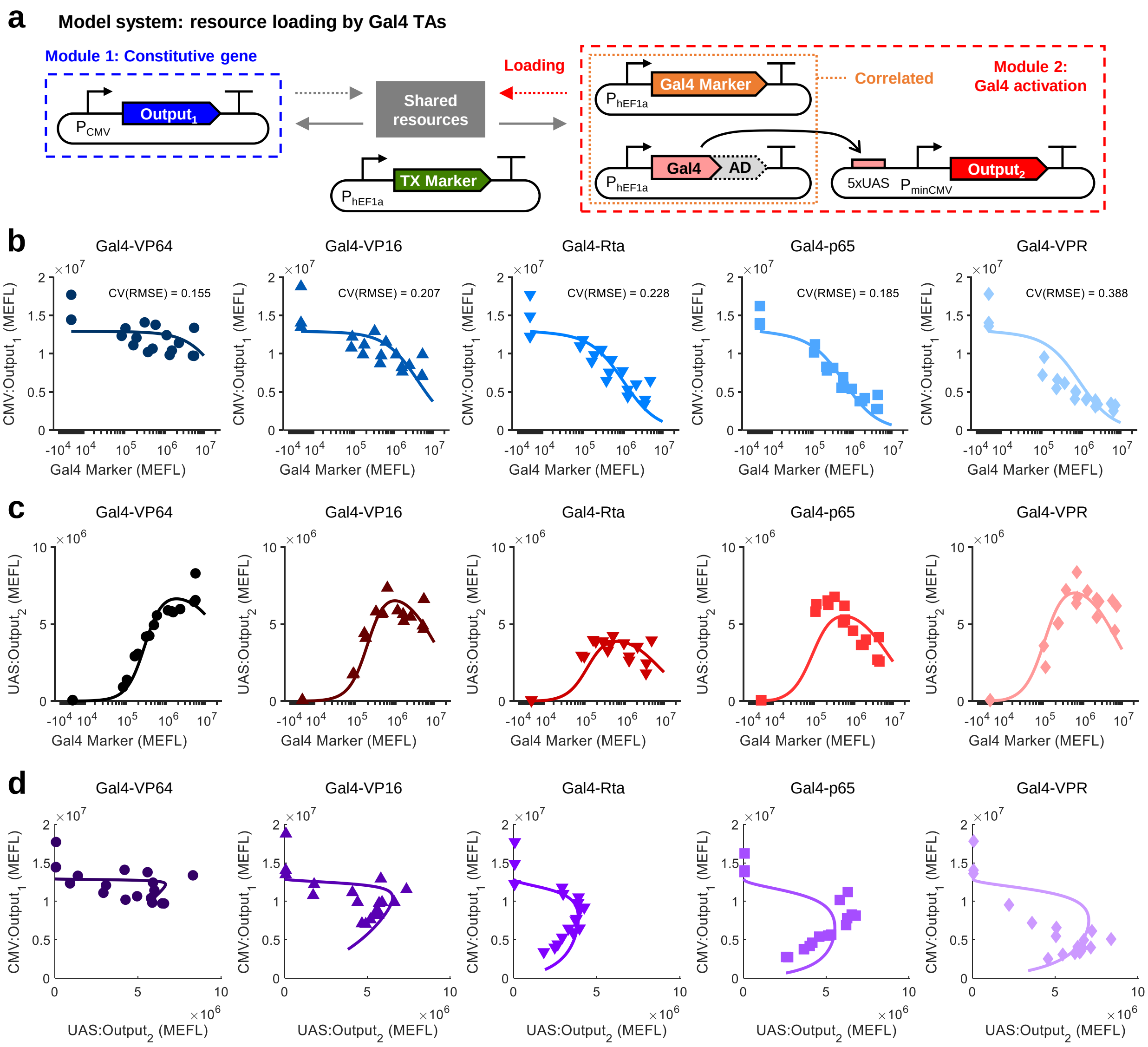

Supplementary Figure 2: Comparison of Gal4 dose-response curves for activation of the UAS promoter. **(a)** Reprinted genetic diagram for the experimental model system shown in Figure 1e. **(b)** Dose-response of CMV:Output$_1$ to each Gal4 TA (reproduced from Figure 1f). **(c)** Dose-response of UAS:Output$_2$ to each Gal4 TA. The markers indicate median expression levels from three experimental repeats. The lines represent fits of our steady-state resource competition model (equation (41)). The CV(RMSE) is the root-mean-square error between the model and data, normalized by the mean of the data. **(d)** Representation of the trade-off between on-target UAS:Output$_2$ activation and non-target CMV:Output$_1$ knockdown by each Gal4 TA, with the model fit overlaid. All data were measured by flow cytometry at 48 hours post-transfection in HEK-293FT cells. Measurements of Output$_i$ were made on cells gated positive for the transfection marker (TX Marker) or Output$_i$, and are shown separately for each experimental repeat. Median values and fit parameters are provided in Source Data.

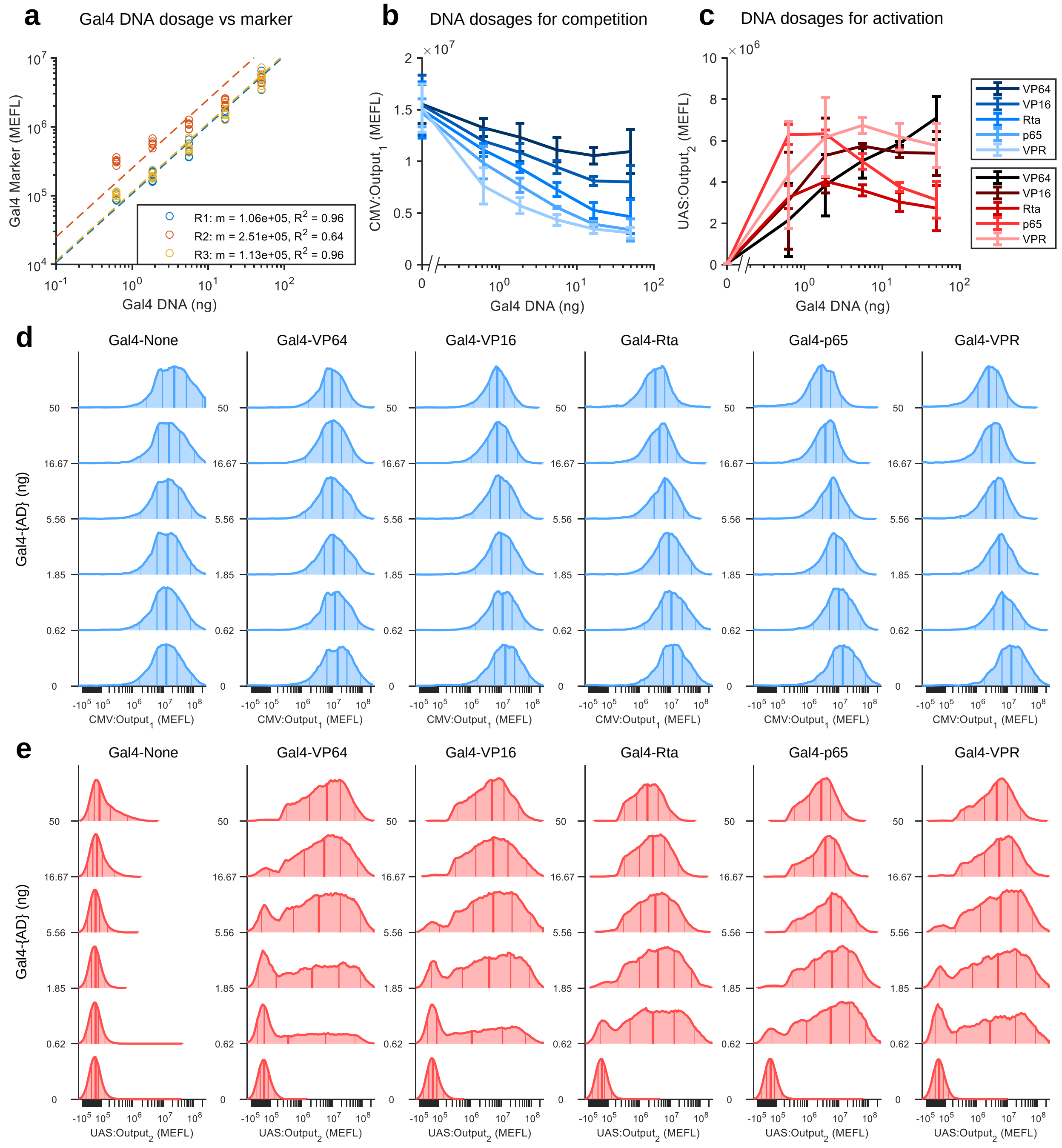

Supplementary Figure 3: Effect of Gal4 transcriptional activators (per DNA dosage). **(a)** Ratios between DNA dosage (ng of DNA of Gal4 TA plasmids) to fluorescent measurements of the co-titrated Gal4 Marker for the experiment shown in Figure 1e. The overall ratios (m) were computed for each experimental repeat (Ri) separately by averaging the ratio of Gal4 Marker:Gal4 DNA per sample. R^2 values are computed using residuals from the ratio line. **(b)** Dose-response of CMV:$Output_1$ to each Gal4 TA. The lines and errorbars represent the mean ± standard deviation of median expression levels from three experimental repeats. **(c)** Dose-response of UAS:$Output_2$ to each Gal4 TA. The lines and errorbars represent the mean ± standard deviation of median expression levels from three experimental repeats. **(d)** Representative histograms from the first experimental repeat for CMV:$Output_1$ expression at each DNA dosage of each Gal4 TA. The lines on the histograms denote the 5th, 25th, 50th, 75th, and 95th percentiles. **(e)** Representative histograms from the first experimental repeat for UAS:$Output_2$ expression at each DNA dosage of each Gal4 TA. The number of cells plotted per histogram in Panels (d-e) are provided in Source Data.

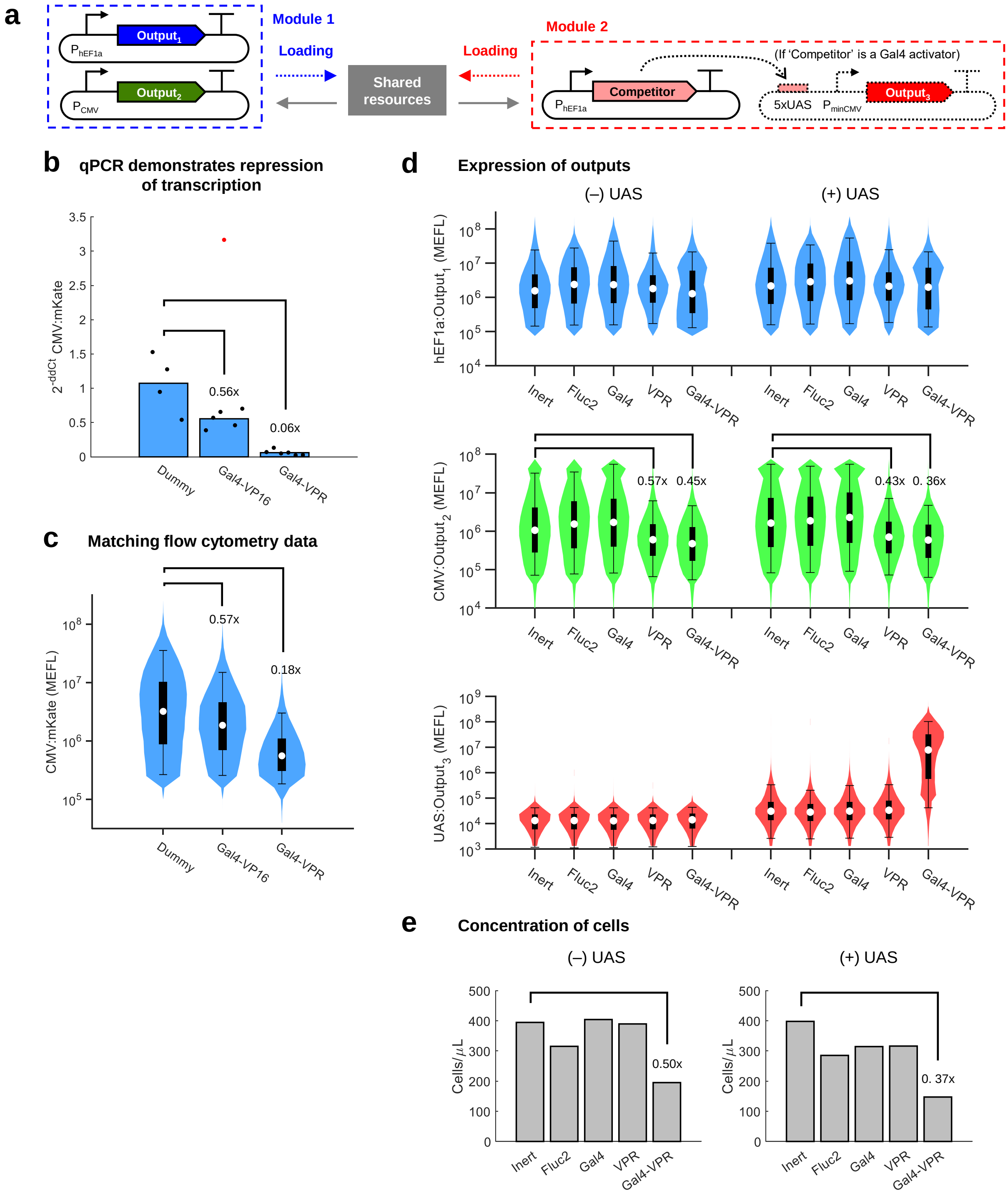

Supplementary Figure 4: Validation of transcriptional resource competition via squelching.

Supplementary Figure 4: (Previous page.) **(a)** Experimental model system for measuring the effects of different putative resource competitors. **(b)** RT-qPCR measurements of CMV:Output$_2$ (mKate2 in this experiment) when co-transfected with a dummy plasmid (cloning vector) vs two Gal4 TAs. Fold-changes in measured Output$_2$ mRNA levels from the sample with dummy plasmid to the samples with Gal4 TAs are shown on the plot. The dots represent two qPCR technical replicates per well of transfected cells. The red dot indicates an outlier which was excluded from the 2^{-ddCt} calculation due to being several standard deviations away from the other points. **(c)** Matching flow cytometry data from the same samples in Panel (b), split right before measurement of mRNA by qPCR and protein levels by flow. Fold-changes in measured Output$_2$ protein levels from the sample with dummy plasmid to the samples with Gal4 TAs are shown on the plot. Violin plots show the distribution of mKate2 expression in transfected cells. The inset box plots show the median (white dot), 25th to 75th percentiles (thick box), and 5th to 95th percentiles (errorbars). Transfected cells were defined as positive for mKate. **(d)** Comparison of different putative resource competitors or controls on expression of hEF1a, CMV, and UAS promoters. Violin plots show the distribution of each reporter in transfected cells. The inset box plots show the median (white dot), 25th to 75th percentiles (thick box), and 5th to 95th percentiles (errorbars). Transfected cells were determined as such for each reporter: hEF1a:Output$_1$ (mNeonGreen): positive for Output$_1$. CMV:Output$_2$ (mKO2): positive for either Output$_1$ or Output$_2$. UAS:Output$_3$ (TagBFP): positive for either Output$_1$ or Output$_3$. The x-labels indicate the competitor in Module 2. Inert indicates a control plasmid with no promoter upstream of the Fluc2 coding sequence. Half of the samples replaced the UAS:Output$_3$ plasmid with Inert as filler DNA, as indicated above all the plots. Fold-changes in median CMV:Output$_2$ from samples co-transfected with the Inert plasmid to that of the samples co-transfected with hEF1a:VPR or hEF1a:Gal4-VPR are shown on the plots. **(e)** Concentration of cells measured by flow cytometry. Fold-changes in concentration from the samples co-transfected with the Inert plasmid to that of the samples co-transfected with hEF1a:Gal4-VPR are shown on the plots. All data were measured at 48 hours post-transfection in HEK-293FT cells. qPCR calculations, median values, and the number of cells plotted per violin are provided in Source Data.

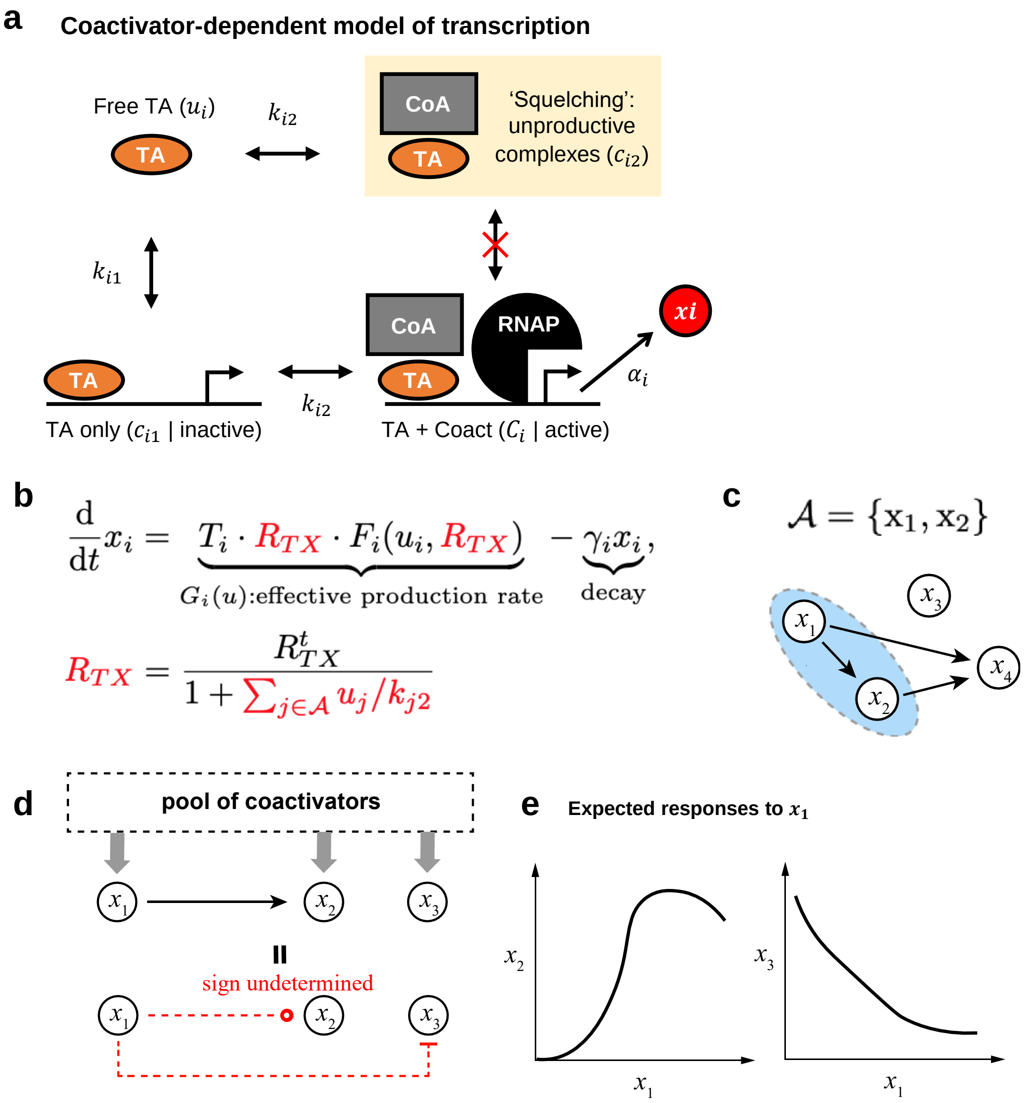

Supplementary Figure 5: Resource competition model. **(a)** Schematic illustrating the species considered in our model of transcriptional activation. TAs bind to coactivators (CoAs) either at the target promoter (C_i) or in solution/at off-target DNA loci (unproductive complexes, C_{i2}). Key dissociation constants for TA-CoA-promoter binding are indicated on the schematic. c_{i2} is assumed to be inaccessible to free promoters or to c_{i1}. **(b)** Reduced ODE model of transcription of mRNA x_i by TAs (u_i), derived from equation (33). $G_i(u)$ indicates the effective production rate of x_i, which depends not just on u_i, but each regulator u_i of every gene in $\mathcal{A}$. **(c)** Illustration of $\mathcal{A}$: the set of genes in a system whose transcription depends on TAs (and not just basal transcription). **(d)** Schematic of hidden interactions among genes due to resource competition. Sequestration of CoAs by a TA (x_1) causes negative effects on non-target genes (x_3). Because this sequestration can occur in solution and at off-target DNA loci (and thus not just at on-target promoters), the transcription rate of the on-target gene is not a monotonically increasing function of x_1, and may in fact decrease as x_1 is increased. **(e)** Expected qualitative dose-responses of x_2 and x_3 to x_1 based on the model of resource competition.

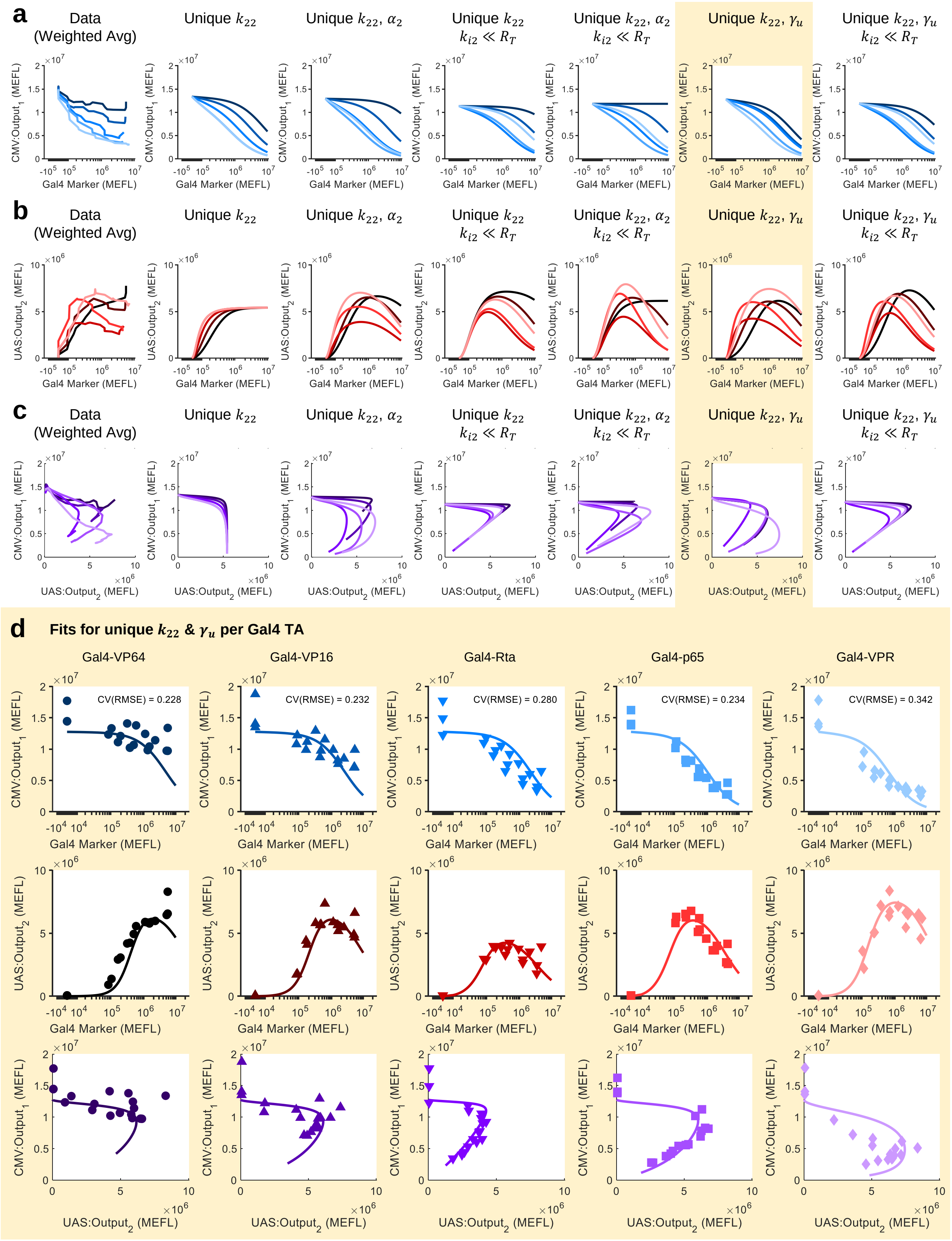

Supplementary Figure 6: Comparison of model fitting schemes.

Supplementary Figure 6: (Previous page.) **(a-c)** The left-most plots show the weighted average expression levels of (a) CMV:Output$_1$, (b) UAS:Output$_2$, and (c) the trade-off between the two for the data shown in Figure 1f and Supplementary Figure 2b-c (with corresponding colors of lines). The other plots show lines of the model when fit under different assumptions about which parameters are different for each Gal4 TA (*i.e.* unique), rather than forced to be equivalent. The third column (*i.e.* unique k_{22}, α_i) corresponds to the fits shown in Figure 1f and Supplementary Figure 2b-c. **(d)** Comparison of fits to data for individual Gal4 TAs under the assumption that only the k_{22} and γ_u parameters are different between Gal4 TAs. The CV(RMSE) is the root-mean-square error between the model and data, normalized by the mean of the data. Fit parameters for each scheme are provided in Source Data.

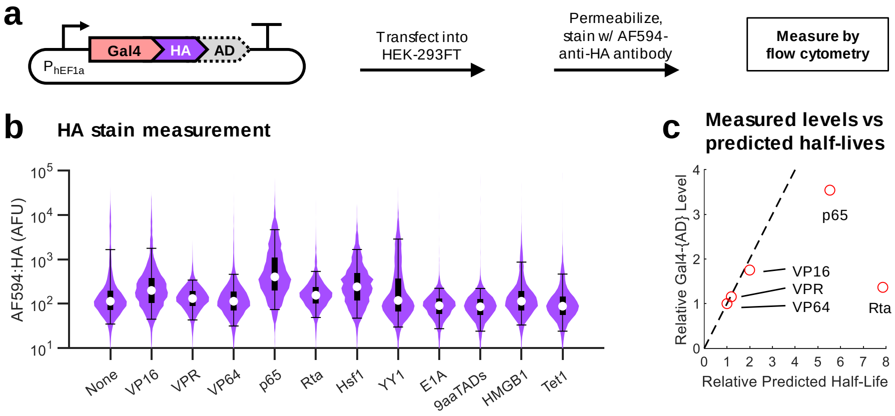

Supplementary Figure 7: Relative expression level of Gal4 TAs. **(a)** Experimental scheme: HA-tagged Gal4 TAs were transfected into HEK-293FT cells along with a TX Marker. After 48 hours, the cells were permeabilized and stained with an antibody against the HA tag (Alexa Fluor 594-anti-HA). **(b)** Violin plots showing the distribution of AF594-HA in transfected cells. The inset box plots show the median (white dot), 25th to 75th percentiles (thick box), and 5th to 95th percentiles (errorbars). Transfected cells were defined as positive for either AF594-HA or a hEF1a-driven TX Marker. **(c)** Comparison of measured Gal4 TA levels to the expected half-lives based on fit degradation rates in Supplementary Figure 6d. Both values are normalized to the lowest value in order to facilitate easier relative comparison. The number of cells plotted per violin are provided in Source Data.

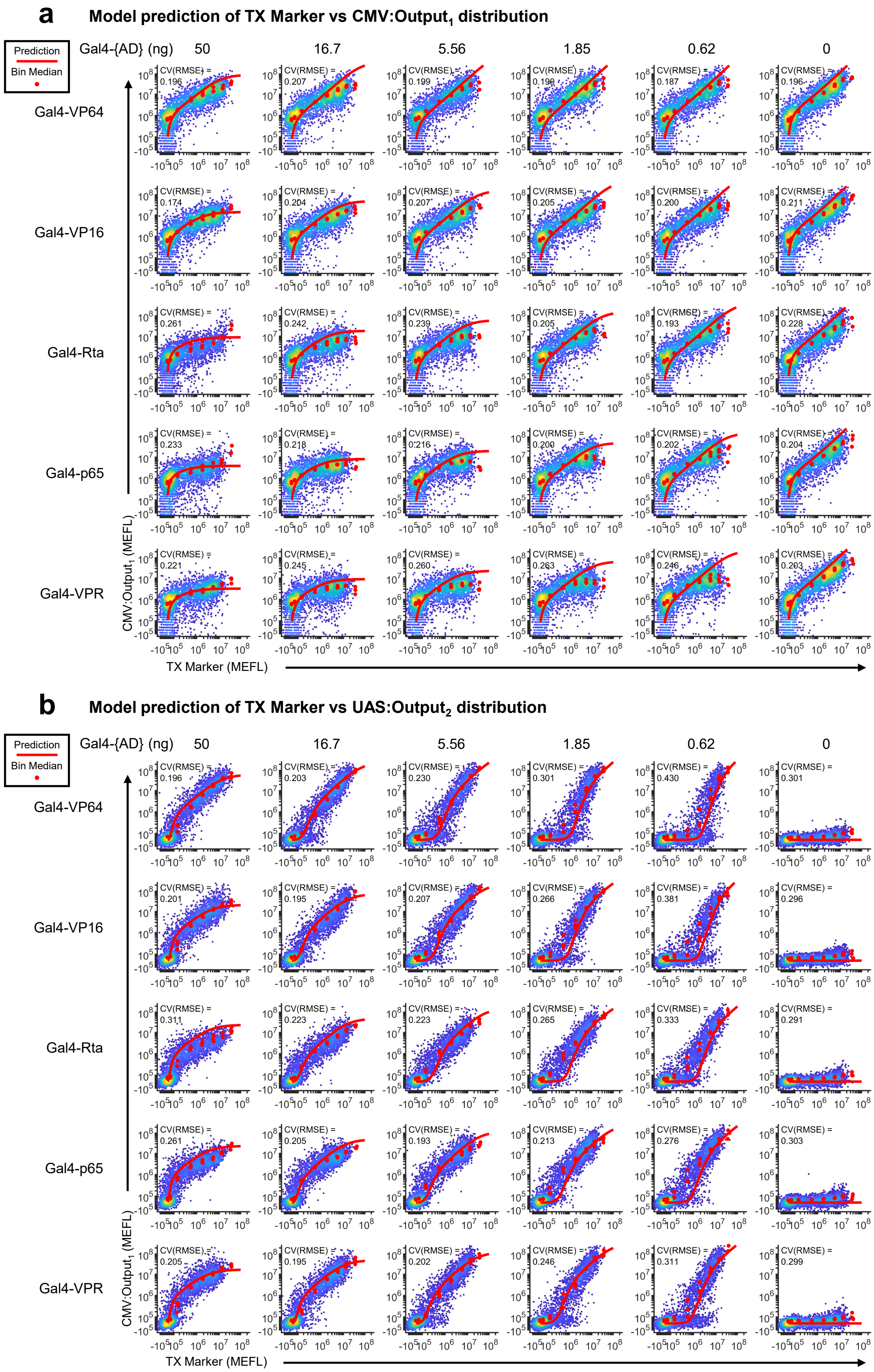

Supplementary Figure 8: Model prediction of 2D transfection distributions.

Supplementary Figure 8: (Previous page.) **(a-b)** Comparisons of model predictions of (a) CMV:Output$_1$ and (b) UAS:Output$_2$ levels at each plasmid copy number across the entire distribution of transfected cells. Predictions were made with equation (42) for CMV:Output$_1$ and UAS:Output$_2$, respectively. The CV(RMSE) is the root-mean-square error between the model and data, normalized by the mean of the data ($\log_{10}$-transformed first since the cell-to-cell variance is approximately log-normally distributed). The red dots represent the median level of Output$_i$ in half-log-decade spaced bins of the TX Marker; one dot for each replicate is shown. The cells are colored by density and are representative from the first experimental repeat. The model parameters were taken from the fit with unique k_{22}, α_2 (*i.e.* the same ones used in Figure 1f and Supplementary Figure 2b-c). To facilitate better comparability between plots, each sample was sub-sampled with the same number of cells (n = 3,000).

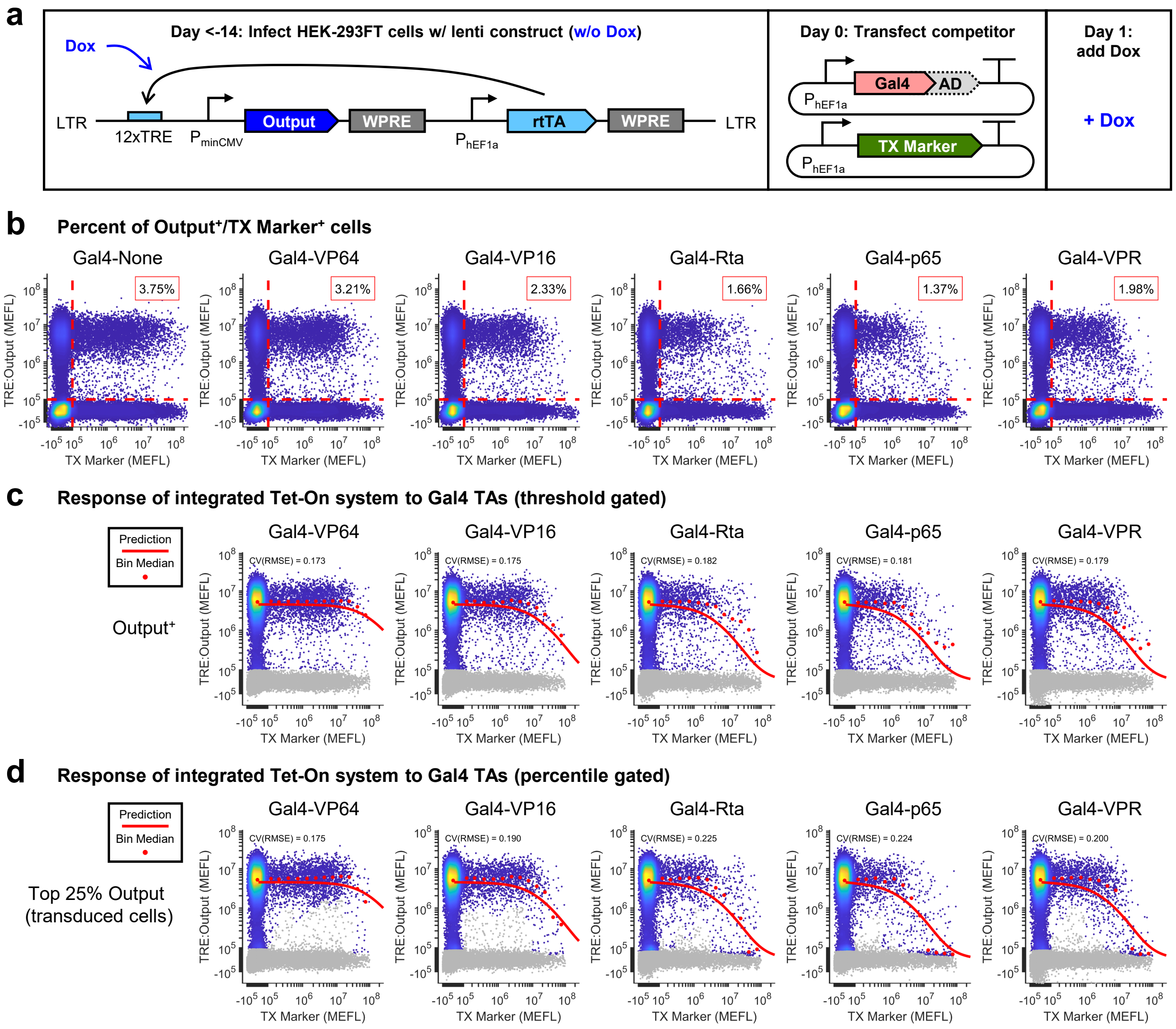

Supplementary Figure 9: Effect of resource loading by Gal4 TAs on a genomically-integrated Tet-On system. **(a)** Genetic diagram of lentiviral construct and plasmids used to test the response of a genomically-integrated Tet-On system to resource loading by Gal4 TAs. At least two weeks before transfection of the Gal4 TAs, the lentiviral construct was integrated into HEK-293FT cells. The cells were expanded and later transfected with Gal4 TAs and a TX Marker to indicate the amount of DNA delivered to each cell. One day after transfection, 1 μg/mL Dox was added to induce TRE:Output expression. **(b)** Percent of cells positive for the TX Marker and TRE:Output. Fluorescent threshold gates used to make the calculation were the same for each sample and are shown on the plots. **(c-d)** Comparison of model prediction to data. The red dots indicate the median level of TRE:Output in quarter-log-decade spaced bins of the TX Marker. The data was either gated (c) on the TRE:Output$^+$ threshold or (d) on the top 25th percentile of TRE:Output expressing cells (approximately the percent that have TRE:Output above its threshold in untransfected (*i.e.* TX Marker less than its threshold) cells. Cells passing each respective gate are colored by density and are used for calculating the medians. The cells not passing the gates are shown in grey. The CV(RMSE) is the root-mean-square error between the model and data, normalized by the mean of the data (biexponentially-transformed first since the cell-to-cell variance is approximately log-normally distributed). The model parameters were taken from the fits in Figure 1f (*i.e.* unique k_{22}, α_2). Since different promoters usually have different transcription rates, the α_1 parameter was adjusted from the CMV data to this TRE data by the ratio of the median level of TRE:Output in the untransfected cells to the median level of CMV:Output$_1$ expression in cells transfected with 0 ng Gal4-{AD} (averaged across each 0 ng sample). To facilitate better comparability among plots, each sample was sub-sampled with the same number of cells (n = 20,000). Median values per bin are provided in Source Data.

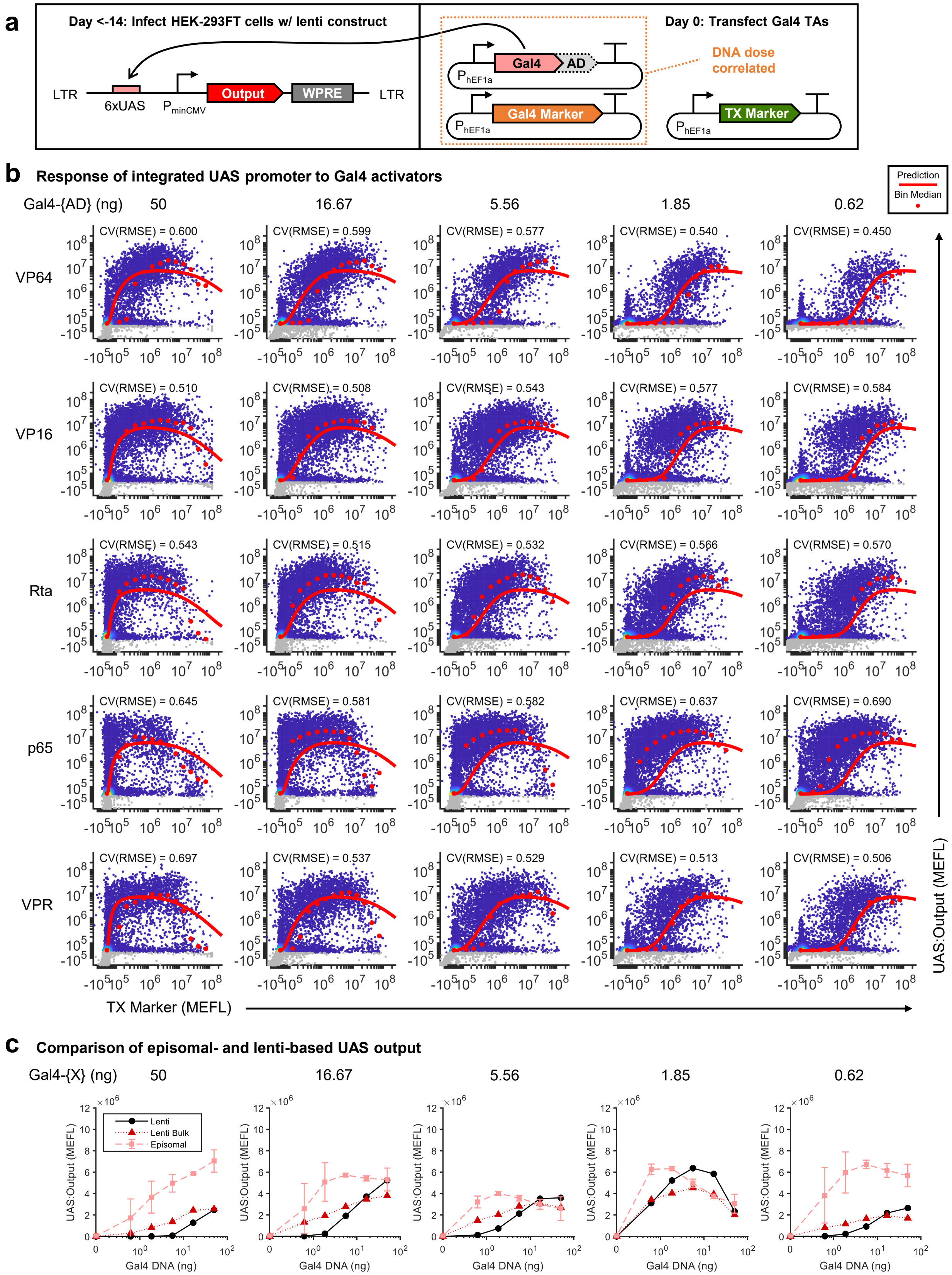

Supplementary Figure 10: Self-squelching of Gal4 TAs driving genomically-integrated UAS promoters.

Supplementary Figure 10: (Previous page.) **(a)** Genetic diagram of lentiviral construct and plasmids used to test the response of a genomically-integrated UAS promoter to transfected Gal4 TAs. At least two weeks before transfection of the Gal4 TAs, the lentiviral construct was integrated into HEK-293FT cells. The cells were expanded and later transfected with Gal4 TAs and a TX Marker to indicate the amount of DNA delivered to each cell. Different samples received dosages of Gal4 TAs and a corresponding amount of Gal4 Marker consistent with the experiment in Figure 1 and Supplementary Figure 2. **(b)** Comparison of model prediction to data. The red dots indicate the median level of UAS:Output in quarter-log-decade spaced bins of the TX Marker. The data was gated on the top 90th percentile of UAS:Output expressing cells (approximately the maximum percent of cells per TX Marker bin that have UAS:Output above background expression for each sample). Cells passing the gate are colored by density and used for calculating the medians. The cells not passing the gates are shown in grey. The CV(RMSE) is the root-mean-square error between the model and data, normalized by the mean of the data (biexponentially-transformed first since the cell-to-cell variance is approximately log-normally distributed). The model parameters were taken from the fits in Supplementary Figure 2b (*i.e.* unique k_{22}, α_2. To facilitate better comparability among plots, each sample was sub-sampled with the same number of cells (n = 20,000). **(c)** Comparison of UAS:Output in the episomal and lenti contexts. The episomal data are equivalent to that shown in Supplementary Figure 3c (errorbars represent the mean ± s.d. of measurements from three experimental repeats). The 'Lenti' line corresponds to the median UAS:Output level in cells gated positive for either UAS:Output or TX Marker. The 'Lenti Bulk' line corresponds to the mean UAS:Output level in the entire (not percentile-gated) population of cells, thereby approximating a bulk measurement. Median values per bin are provided in Source Data.

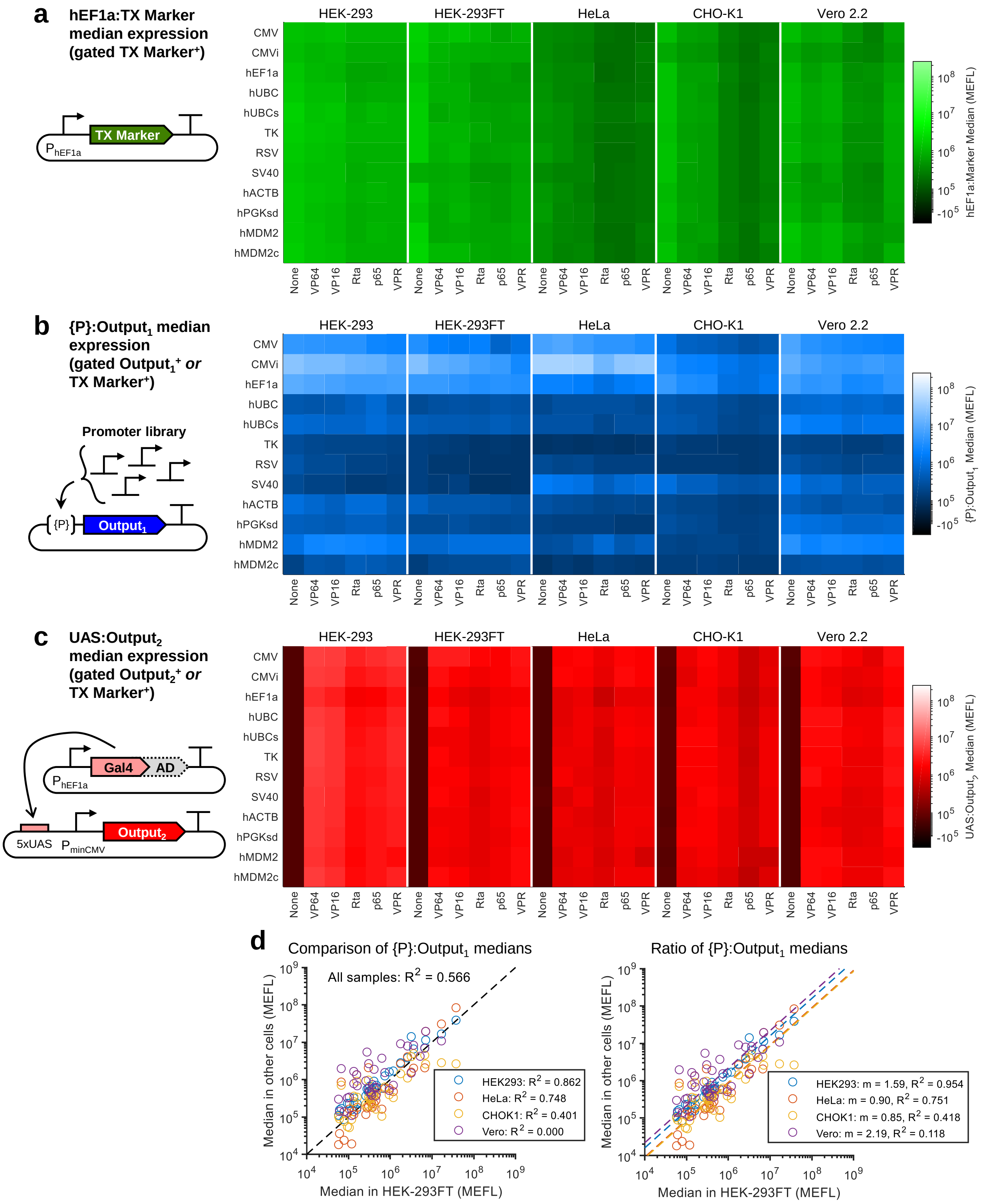

Supplementary Figure 11: Expression of each reporter in the experiment characterizing resource loading effects across cell lines.

Supplementary Figure 11: (Previous page.) **(a-c)** Median expression levels of (a) TX Marker, (b) {P}:$Output_1$, and (c) UAS:$Output_2$ for the data in Figure 2). The values shown are the mean of median measurements from three experimental repeats. The data in Panel (b) has an extra autofluorescence subtraction step as described in Methods. **(d)** Comparison of Nominal {P}:$Output_1$ levels between each cell line and HEK-293FT cells (which were used for most other experiments). In the plot on the left, R^2 is computed for the Nominal {P}:$Output_1$ in non-HEK-293FT cells relative to those in HEK-293FT cells. In the plot on the right, the overall ratios (m) between Nominal {P}:$Output_1$ in each cell line and HEK-293FT cells were computed by averaging the ratio for each sample; the overall ratios are then used as the reference for computing R^2 values. Values from each experimental replicate are shown independently and combined for computing ratios. Median values are provided in Source Data.

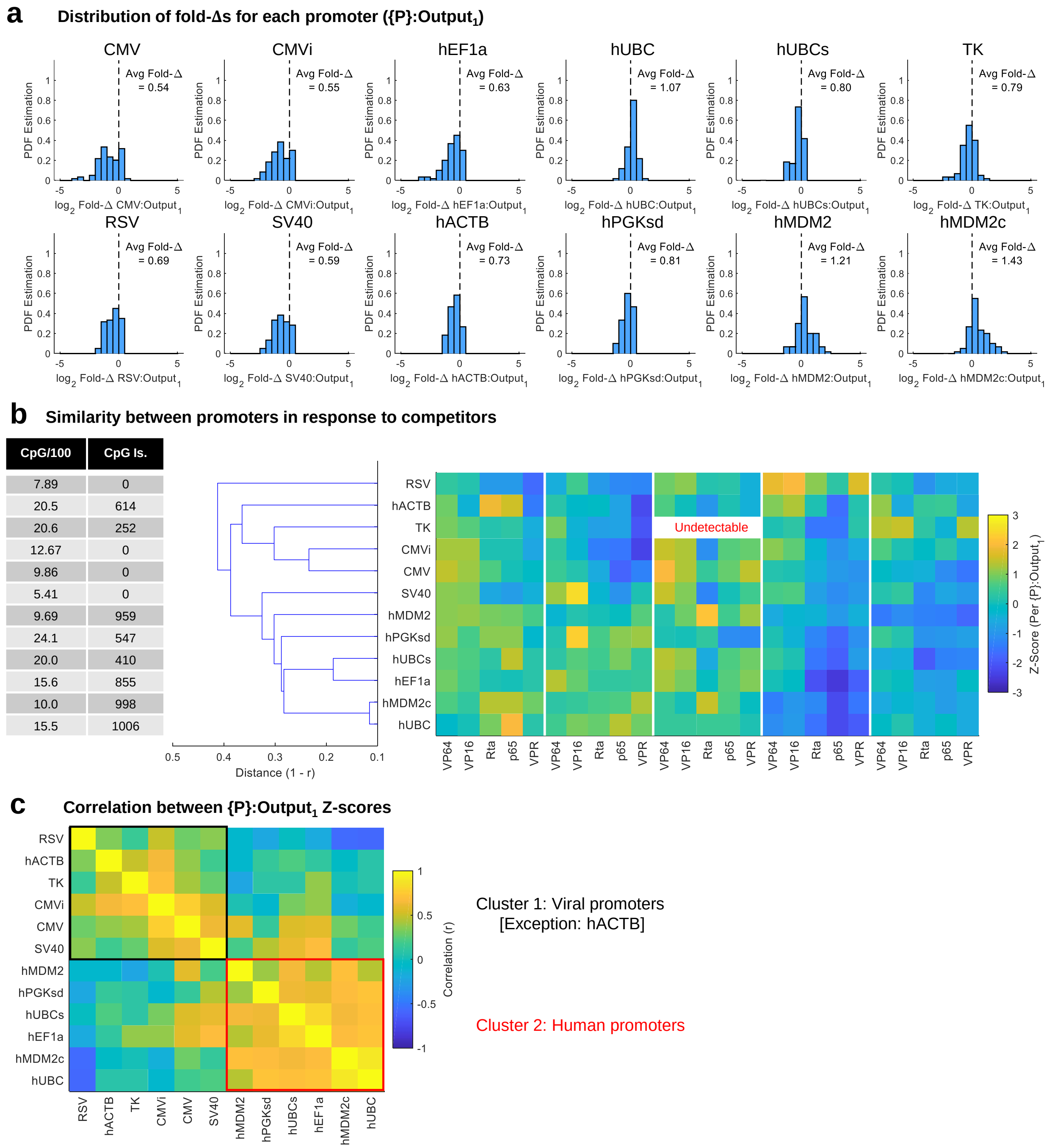

Supplementary Figure 12: Comparison of responses of constitutive promoters to resource loading by Gal4 TAs. **(a)** Distribution of Fold-Δs of each promoter across cell lines and Gal4 TAs for the data in Figure 2. Data from three experimental repeats are combined together in the histograms. Average Fold-Δs for each promoter are shown inset in each plot. **(b)** Hierarchical clustering of the z-scores of {P}:Ouptut$_1$ Fold-Δs based on correlation. Z-scores were computed per promoter across all combinations of Gal4 TAs and cell lines using the average Fold-Δs from three experimental repeats. On the left are shown the number of CpG motifs per 100 base pairs and approximate size (in base pairs) of any CpG island located in the promoter. The samples with the TK promoter in HeLa cells were determined to be undetectable (see Supplementary Figures 43 & 44). **(c)** Correlation between Fold-Δs for each combination of promoters, with two manually-assigned clusters highlighted. The correlation was computed using the average Fold-Δs from three experimental repeats.

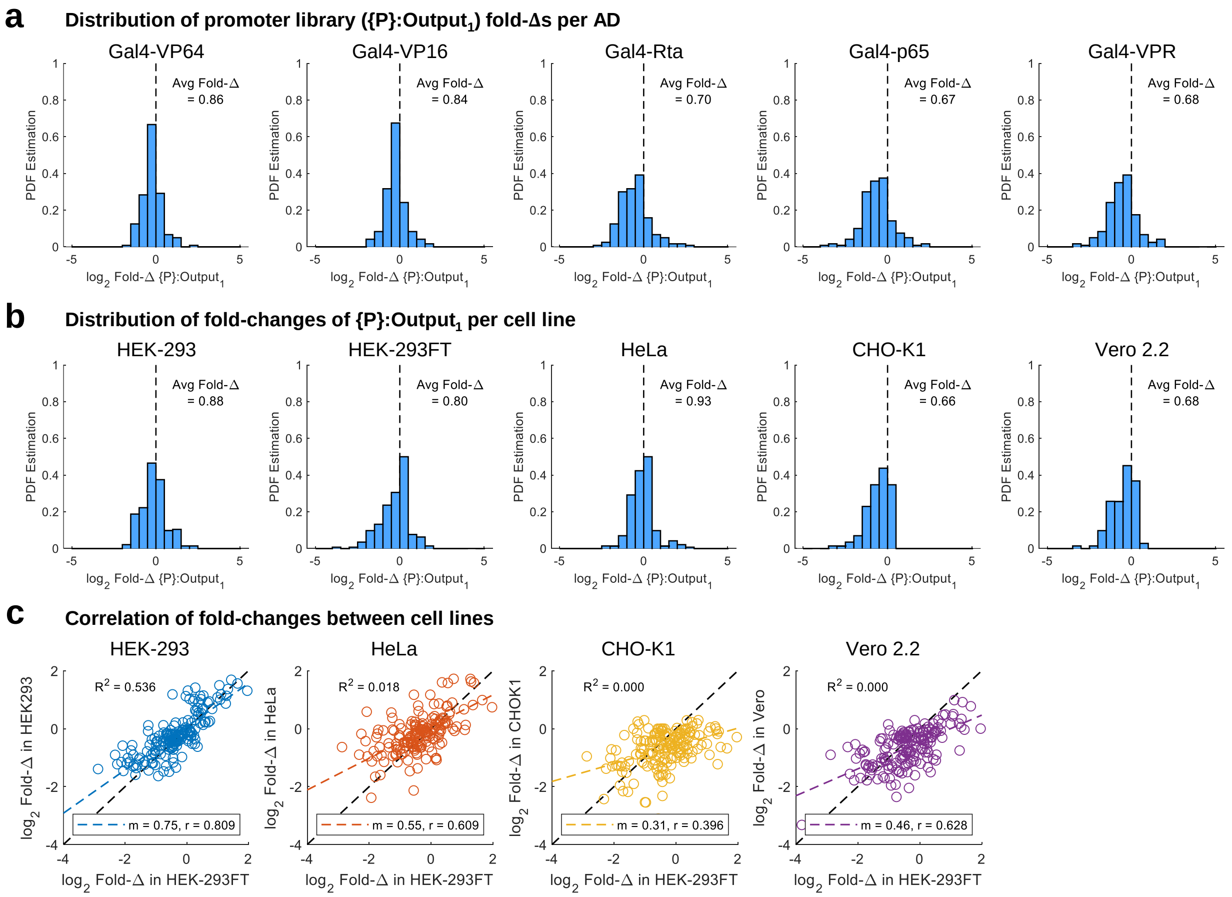

Supplementary Figure 13: Comparison of constitutive promoter knockdown by Gal4 TAs between cell lines. **(a)** Distribution of Fold-Δs of {P}:$Output_1$ caused by each Gal4 TA across promoters and cell lines for the data in Figure 2c. The average Fold-Δ of {P}:$Output_1$ by a given Gal4 TA is shown inset in each plot. **(b)** Distribution of Fold-Δs of {P}:$Output_1$ across all promoters and Gal4 TAs per cell line. The average Fold-Δ of {P}:$Output_1$ in a given cell line is shown inset in each plot. **(c)** Correlation in Fold-Δs of {P}:$Output_1$ between HEK-293FT cells (which were used for most experiments in the paper) and other cell lines. The R^2 values correspond to a direct comparison of Fold-Δs between HEK-293FT cells and the other cell lines. Panels (a-c) combine data from all three experimental repeats.

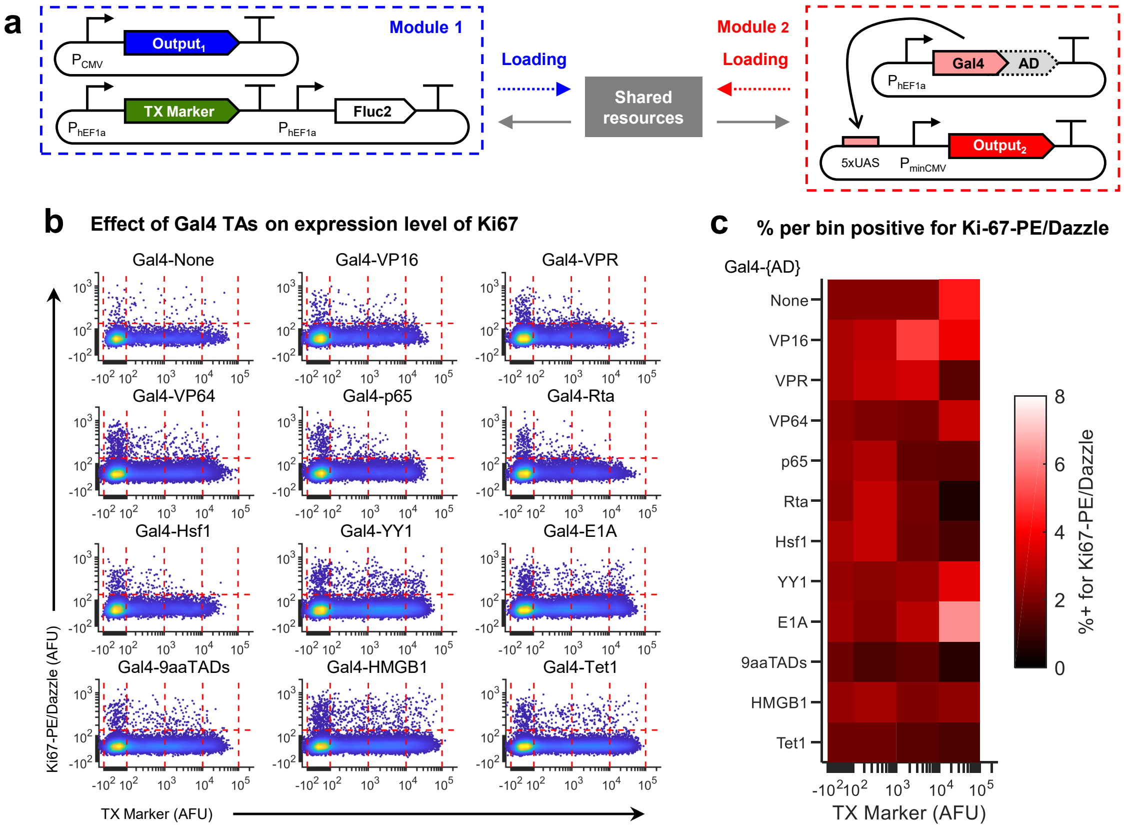

Supplementary Figure 14: Effect of Gal4 TAs on the concentration of cells measured with flow cytometry. **(a)** Genetic diagram of the experiment to test effects of Gal4 TAs on growth rate inhibition. The plasmid with the TX Marker and Fluc2 is the same as the control plasmid in Supplementary Figures 25-31 in order to be similar to the iFFL tests while also retaining elements from the Gal4 TA comparison in Supplementary Figure 1. 48 hours after transfection, cells were fixed, permeabilized, and stained with an antibody against Ki-67 (PE/Dazzle-anti-Ki-67), a marker of cell division. The reporters are: $Output_1$: TagBFP, $Output_2$: iRFP720, TX Marker: mNeonGreen. **(b)** Comparison of Ki-67 staining across transfection levels for each GAl4 TA. Cells were separated into log-decade-width bins (as indicated by the vertical lines). The horizontal line is the threshold above which cells were considered to be Ki-67 positive. Cells are colored by density. **(c)** Percent of PE/Dazzle-Ki-67+ cells in each bin for each sample.

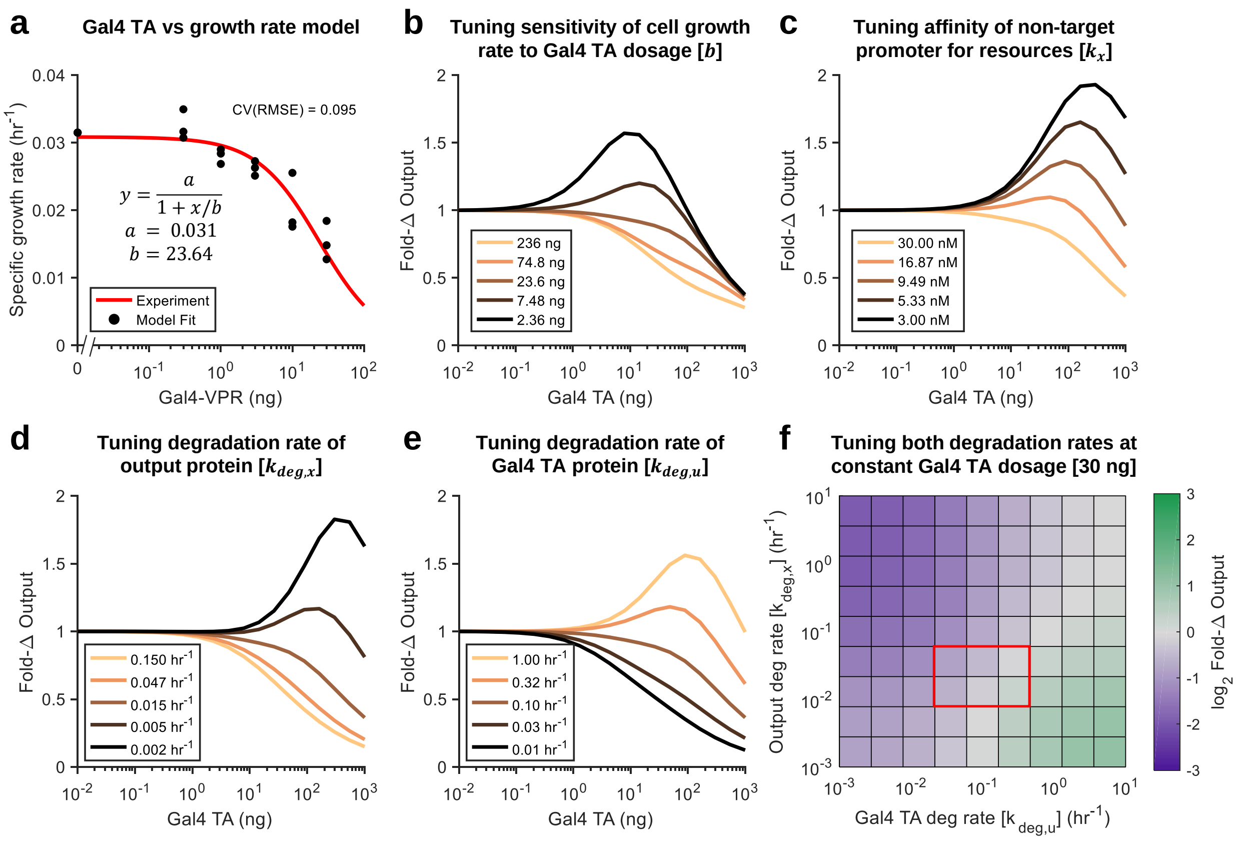

Supplementary Figure 15: Combined effects of growth inhibition and resource loading on non-target gene expression levels. **(a)** Phenomenological model fit for the effect of Gal4-VPR on HEK-293FT growth rate using data from Supplementary Figure 30b. The growth rate is estimated based on the fold-changes in cell density (see Supplementary Note 5). **(b-e)** Simulation of changes in the output level of theoretical non-target constitutive gene(s) in response to theoretical Gal4 TA(s) in which (b) the TAs have varying effects on the cell growth rate [b], (c) the non-target promoters have varying affinity for transcriptional resources [k_x], (d) the output proteins have varying degradation rates [$k_{deg,x}$], or (e) the Gal4 TAs have varying degradation rates [$k_{deg,u}$]. The Fold-Δ indicates the fold-change in the Output relative to its nominal value when Gal4 DNA = 0. The values are taken at steady-state. **(f)** The combined effects of changing the degradation rates of the output protein and Gal4 TA(s) on the qualitative effect of loading on the output expression level. Here the Gal4 DNA level is fixed at 30 ng. The colormap represents fold changes in output levels relative to the nominal level in the absence of loading. The red box highlights the likely range of experimental parameters based on realistic estimates of mRNA/protein degradation rates. In particular, mRNAs typically have half-lives of ~9 hours[9] (deg rate ~ 0.08 hr^{-1}), fluorescent proteins are typically stable with half-lives of ~26 hours[14] (deg rate ~0.027 hr^{-1}), and transcription factors are typically unstable[9,15] (estimated half-lives are between 2-10 hours, yielding deg rate ~0.07-0.35 hr^{-1}). Simulation parameters are provided in Source Data.

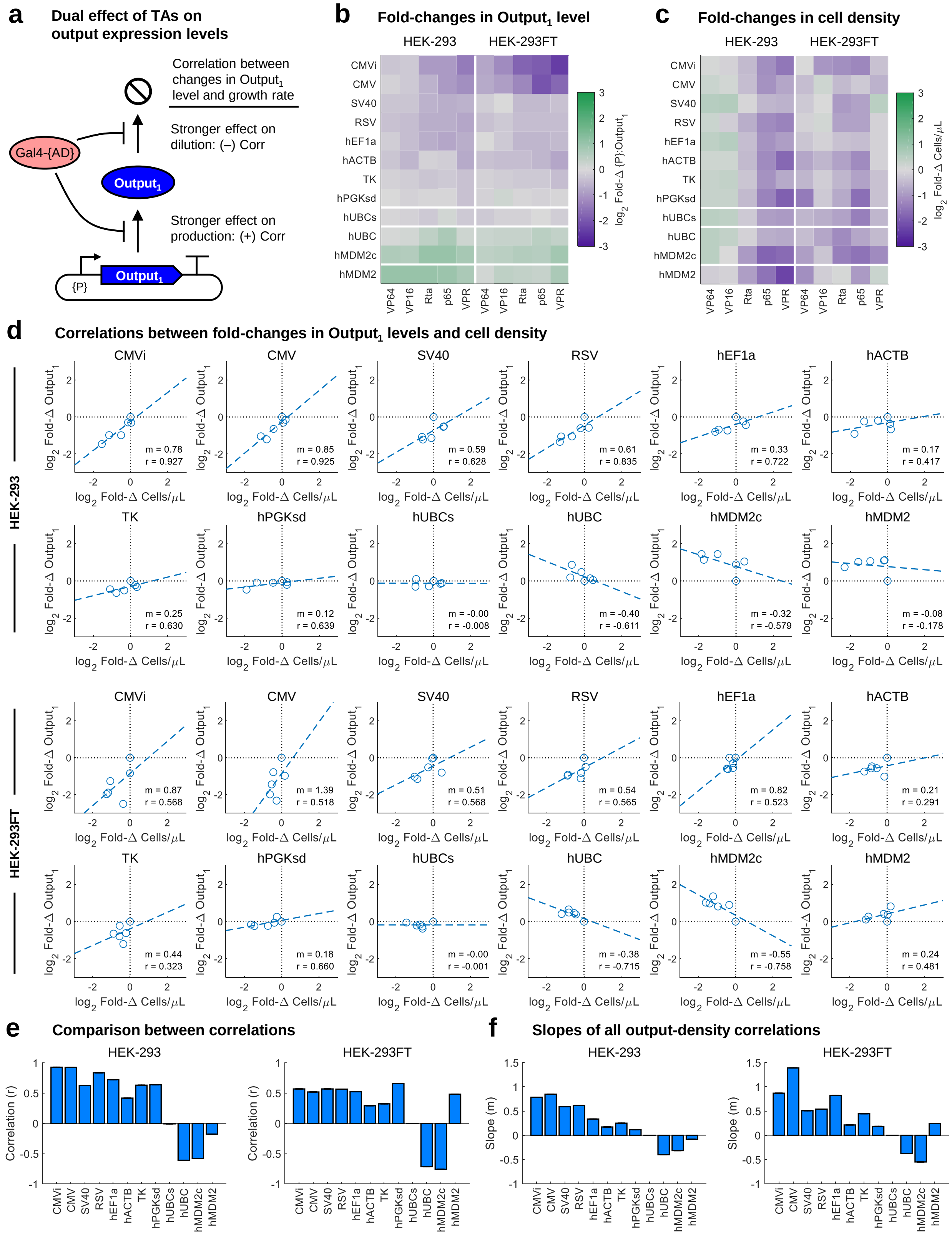

Supplementary Figure 16: Effect of Gal4 TAs on cell density.

Supplementary Figure 16: (Previous page.) **(a)** Illustration of the dual effect of TAs on non-target gene expression output levels based on qualitative findings from simulations in Supplementary Figure 15. For a given level of Gal4-{AD}, the change in the level of {P}:Output$_1$ depends on the relative change in the production and decay rates of Output$_1$ caused by the TA's resource loading and toxicity, respectively. Each promoter (P) has a different sensitivity to resource loading based on the affinity of the promoter for shared resources and identity of resources used by the promoter and competitors (*e.g.* the TAs). For a given decrease in the decay rate of outputs caused by toxicity of Gal4 TAs, we expect that promoters that are more sensitive to resource loading will have their outputs' production rates reduced more than decay rates, causing an overall decrease in output level and a positive correlation between the changes in cell density and output levels. Conversely, promoters with reduced sensitivity to resource loading will experience a stronger effect on the decay rate of their output, causing an overall increase in output level and a negative correlation between the changes in cell density and output levels. **(b)** Fold-Δs in {P}:Output$_1$ expression levels in response to loading by different Gal4 TAs in HEK-293 and HEK-293FT cells. The data are from Figure 2c and rearranged such that promoters are sorted by the average effect of Gal4 TAs on the output level. Only the HEK cell lines are shown because the others are not transfected at a high enough rate (see Supplementary Figure 42) for sensitive measurements of changes in cell density. The horizontal white lines separate promoters into groups that act similarly. The top, most numerous group are promoters with positive correlations in subsequent panels. hUBCs output is uncorrelated with changes in cell density. hUBC and hMDM2c both have negative correlations, and hMDM2 has a unique non-correlated response. **(c)** Fold-Δs in the concentration of cells due to resource loading. Fold-Δs were computed by dividing the concentration of cells in each sample by the concentration of cells in the corresponding sample with the same constitutive promoter in the same cell line, but co-transfected with Gal4-None, which does not load resources. Each heatmap element represents the mean of measurements from three experimental repeats. The promoters are sorted to match the order in Panel (a). **(d)** Correlation between the effects of resource loading on cell concentration and the level of Output$_1$. Each point is the mean measurement from Panels (a-b). m: slope, r: Pearson's correlation. **(e-f)** Comparison of correlations coefficients (e) and slopes (f) for the correlation lines shown in Panel (d).

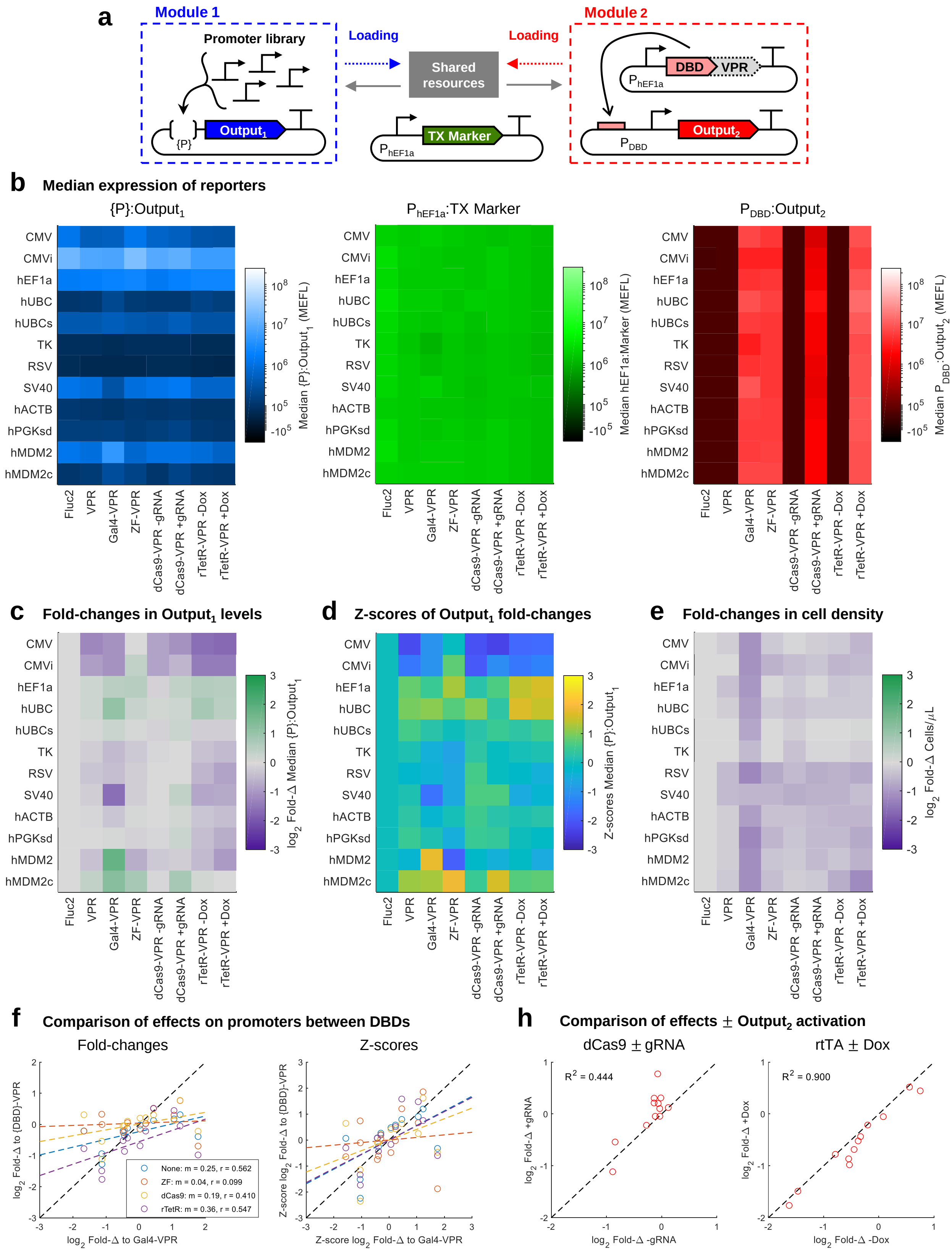

Supplementary Figure 17: Resource loading by TAs with different DBDs but the same AD.

Supplementary Figure 17: (Previous page.) **(a)** Genetic diagram for the experimental model system. Constitutive promoters from the same library as in Figure 2 driving Output_1 (mKO2) were co-transfected with one of several competing modules containing the VPR AD fused to different DBDs driving Output_2 (TagBFP). The TX Marker is mNeonGreen. **(b)** Median expression of the fluorescent reporters. Fluc2 refers to co-transfection with hEF1a:Fluc2 (no VPR domain). VPR refers to co-transfection with hEF1a:VPR (no DBD). All other x-labels indicate the DBD fused to VPR. For dCas9-VPR, (+) or (–) gRNA indicates whether it was co-transfected with a gRNA that targets (+, gRNAb) or doesn't target (–, gRNAa) the promoter driving Output_2 (gRNAs and promoters from Kiani et al[43]). For rTetR-VPR, (+) or (–) indicates whether Dox was added (+, 1 μg/mL) or not (–). Data are from a single experiment. Measurements for {P}:Output_1 were made on cells positive for either Output_1 or TX Marker, for TX Marker were made on cells positive for TX Marker, and for Output_2 were made on cells positive for either Output_2 or TX Marker. **(c)** Fold-Δs in {P}:Output_1 expression in response to different putative competitors. Fold-Δs were computed for each promoter-competitor combination in reference to the sample with the same promoter co-transfected with hEF1a:Fluc2. **(d)** Z-scores of Output_1 Fold-Δs, computed for each competitor across all promoters. **(e)** Fold-Δs in cell density caused by each putative competitor. Fold-Δs were computed for each promoter-competitor combination in reference to the sample with the same promoter co-transfected with hEF1a:Fluc2. **(f)** Correlation between Fold-Δs (left) or Fold-Δ Z-scores (right) of each promoter in response to resource loading by each competitor, compared to Gal4-VPR (since Gal4 was used for most experiments). m: slope, r: Pearson's correlation. **(g)** Comparison of Fold-Δs in the presence and absence of transcriptional activation by dCas9-VPR [left, (+) or (–) the promoter-specific gRNA] or rTetR-VPR [right, (+) or (–) Dox]. R^2 is computed based on the 1:1 ratio line. Median values are provided in Source Data.

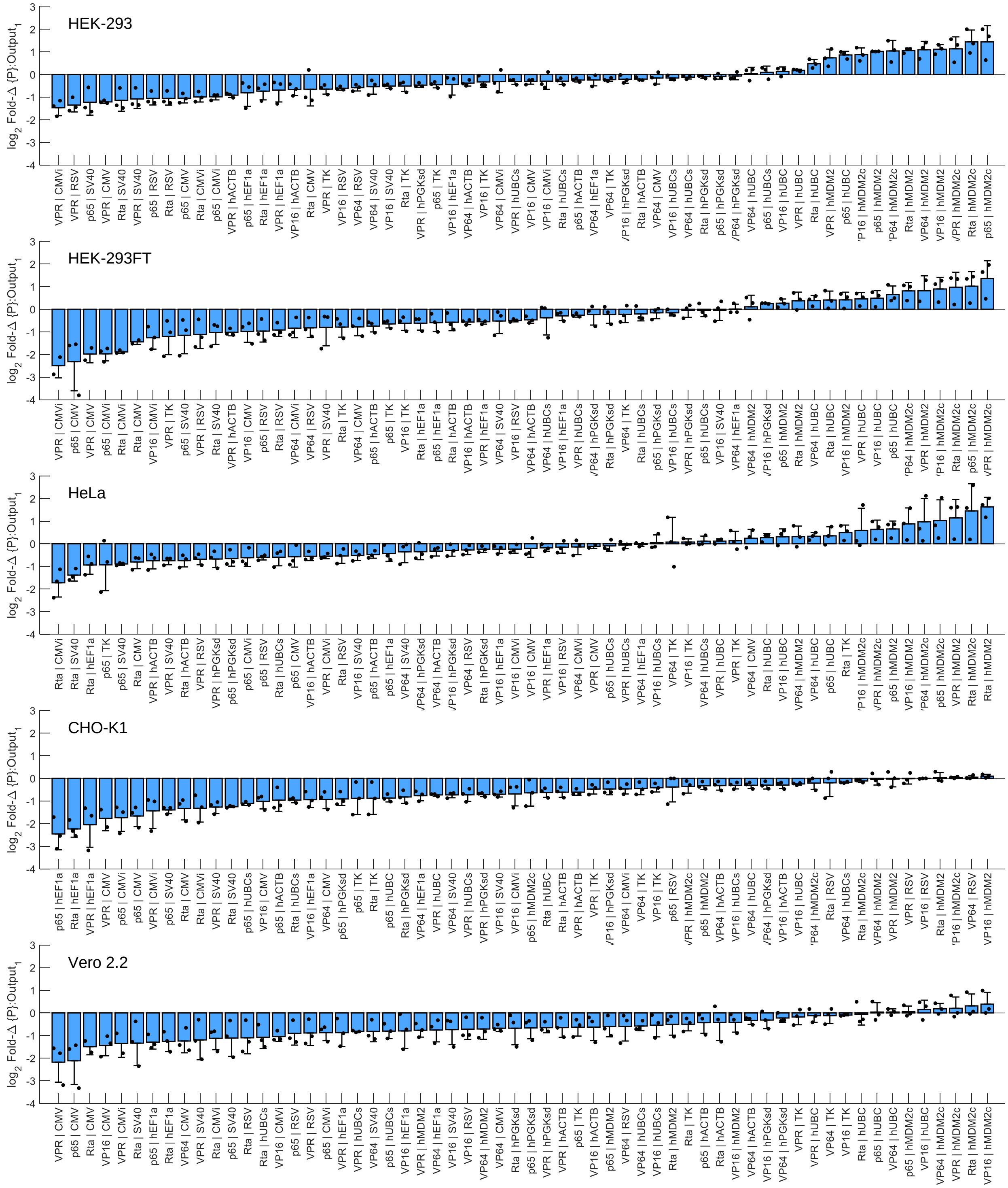

Supplementary Figure 18: All promoter fold-changes, ranked per cell line. This is another representation of the data in Figure 2c. The $\log_2$ Fold-Δs are ranked in each cell line from most negatively to most positively affected. The errorbars represent the mean ± s.d. of measurements from three experimental repeats (represented by the individual points). Note that promoters that are minimally affected by resource loading by Gal4 TAs have $\log_2$ Fold-Δs near zero.

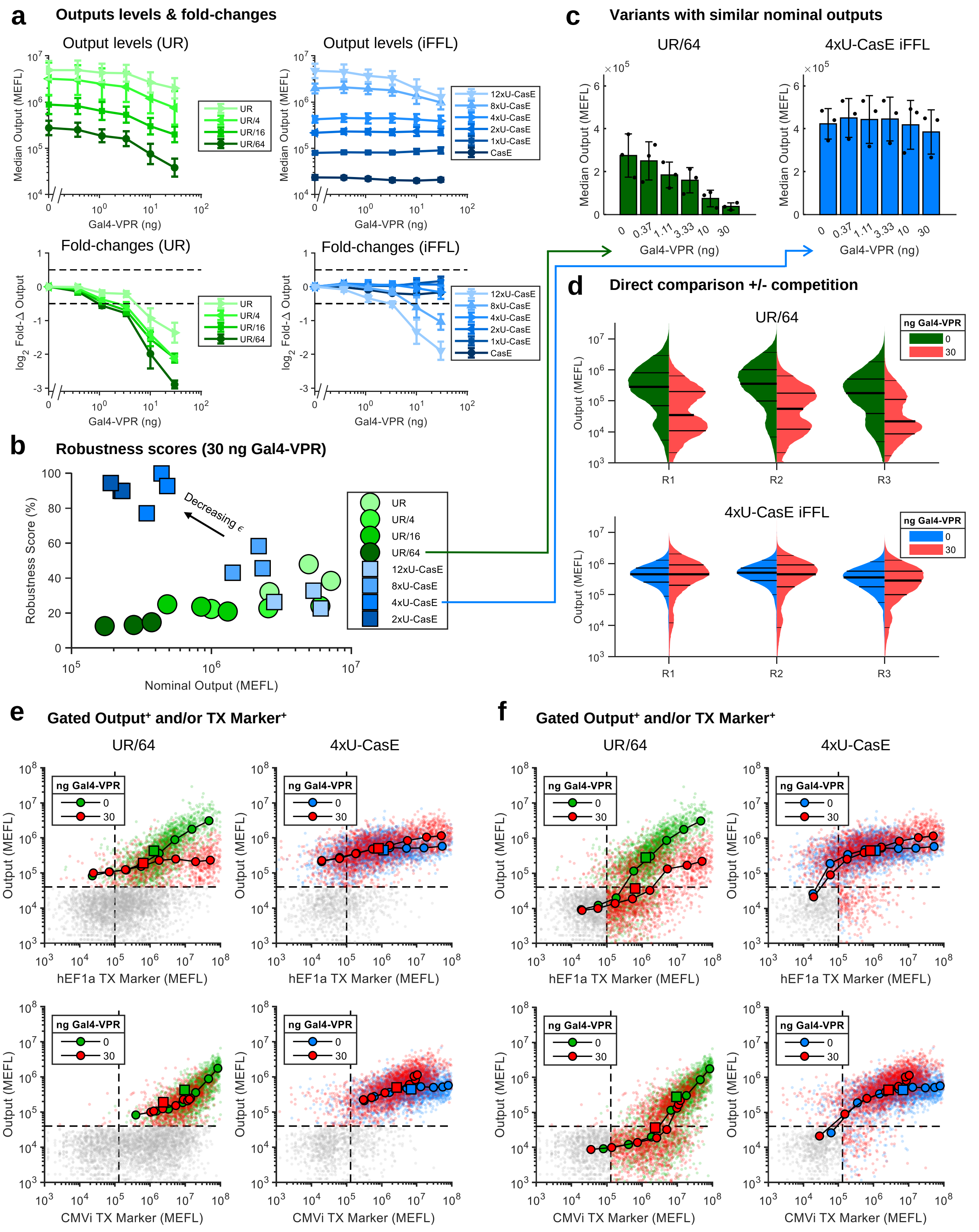

Supplementary Figure 19: Comparison of gating strategies for the CMVi iFFL and UR control outputs in the presence of resource loading.

Supplementary Figure 19: (Previous page.) **(a-d)** The same data and plots comparing incoherent feedforward loop (iFFL) and unregulated (UR) modules as in Figure 4, but with calculations done for cells positive for either Output or TX Marker. Note that in here we can also measure the median level of Output_1 in the samples transfected with CasE with zero or one upstream open reading frames (uORFS – CasE, 1xU-CasE, respectively) because of the gate on the TX Marker. These variants cannot be measured accurately when only gating on the output reporter because of their low expression near the gate threshold. The errorbars represent the mean ± s.d. of measurements from three experimental repeats. **(e-f)** Comparison of scatterplots with and without competition for the samples in Panels (c-d) (Panels (d-e) for Figure 4). The round colored markers show the median level of Output in half-log-decade-spaced bins of the TX Marker. The square colored markers indicate the median Output and TX Marker level in each gated population. In Panel (e), cells were gated only if positive for Output; in Panel (f), cells were gated positive for either Output or TX Marker. Cells not passing the gate(s) are shown in grey. For the plots in Panel (f), all cells were used to compute the bin medians, whereas in Panel (e), only the gated population was used. The first experimental repeat is shown as a representative example. To facilitate better comparability between plots, each sample was sub-sampled to plot the same number of cells (n = 3,000). Median values for both gating strategies and the number of cells plotted per histogram are provided in Source Data.

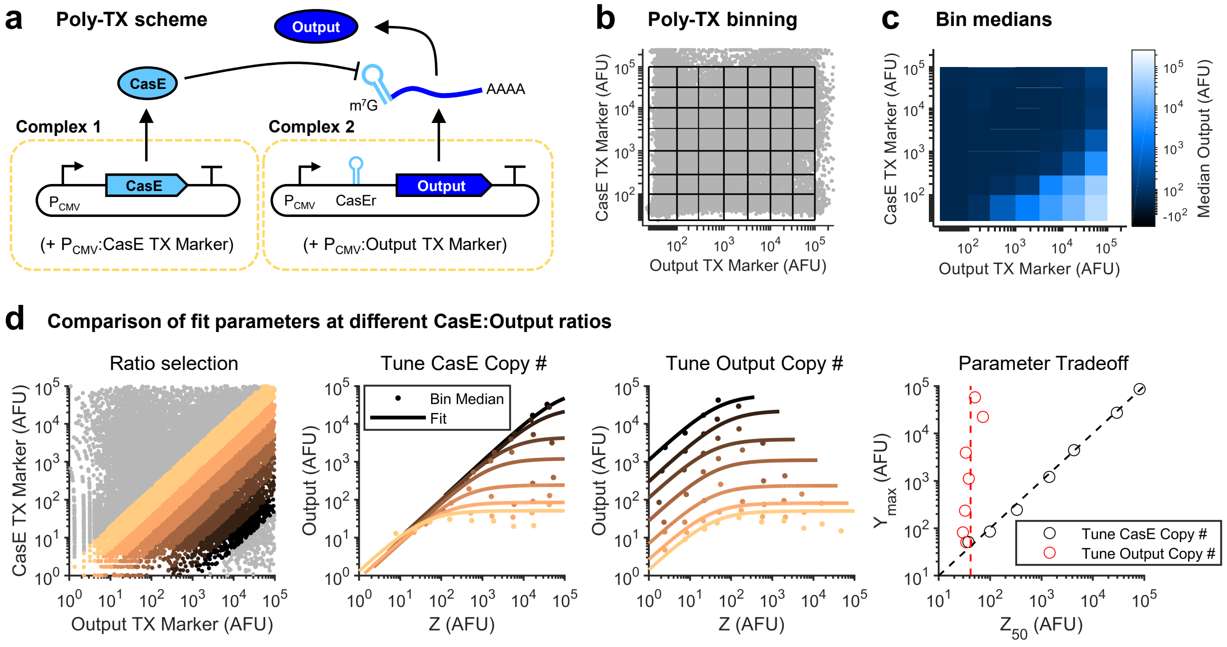

Supplementary Figure 20: Comparison of CasE iFFL at different ratios of endoRNase and output plasmids. **(a)** Schematic of the poly-transfection[25] (Poly-TX) experiment. Constitutively-expressed CasE and a CasE-targeted output reporter (EYFP) were each separately formed into lipid-DNA complexes with TX Markers (CasE Marker: mKOs, Output Marker: TagBFP). The two different Poly-TX complexes were then simultaneously added into a well with HEK-293FT cells, giving de-correlated delivery of the CasE and Output plasmids, with correlated delivery of each with their own TX Marker. **(b)** 2D binning scheme for the fluorescent reporters of each Poly-TX complex overlaid on top of a scatterplot of the cells, showing good coverage of the full 2D input space. **(c)** Median Ouptut expression of cells within each bin. **(d)** Left: Ratiometric binning. cells at particular ratios of each TX Marker are colored according to their assignment to different bins. Center-left/right: Z vs Output (Y) curves with iFFL model fits for different ratios of CasE:Output plasmids, approximating conditions where we tune either the amount of CasE plasmid (center-left) or the amount of Output plasmid (center-right) while holding the other constant. Right: comparison of fit parameters across ratiometric bins when tuning either the amount of CasE or Output plasmid while holding the other constant. The comparison of tuning one plasmid vs the other is enabled because we binned ratiometrically over a wide range of total DNA input concentrations, and can use either TX Marker separately to approximate Z across the ratiometrically-binned cells. Median values per bin and fit parameters per ratio are provided in Source Data.

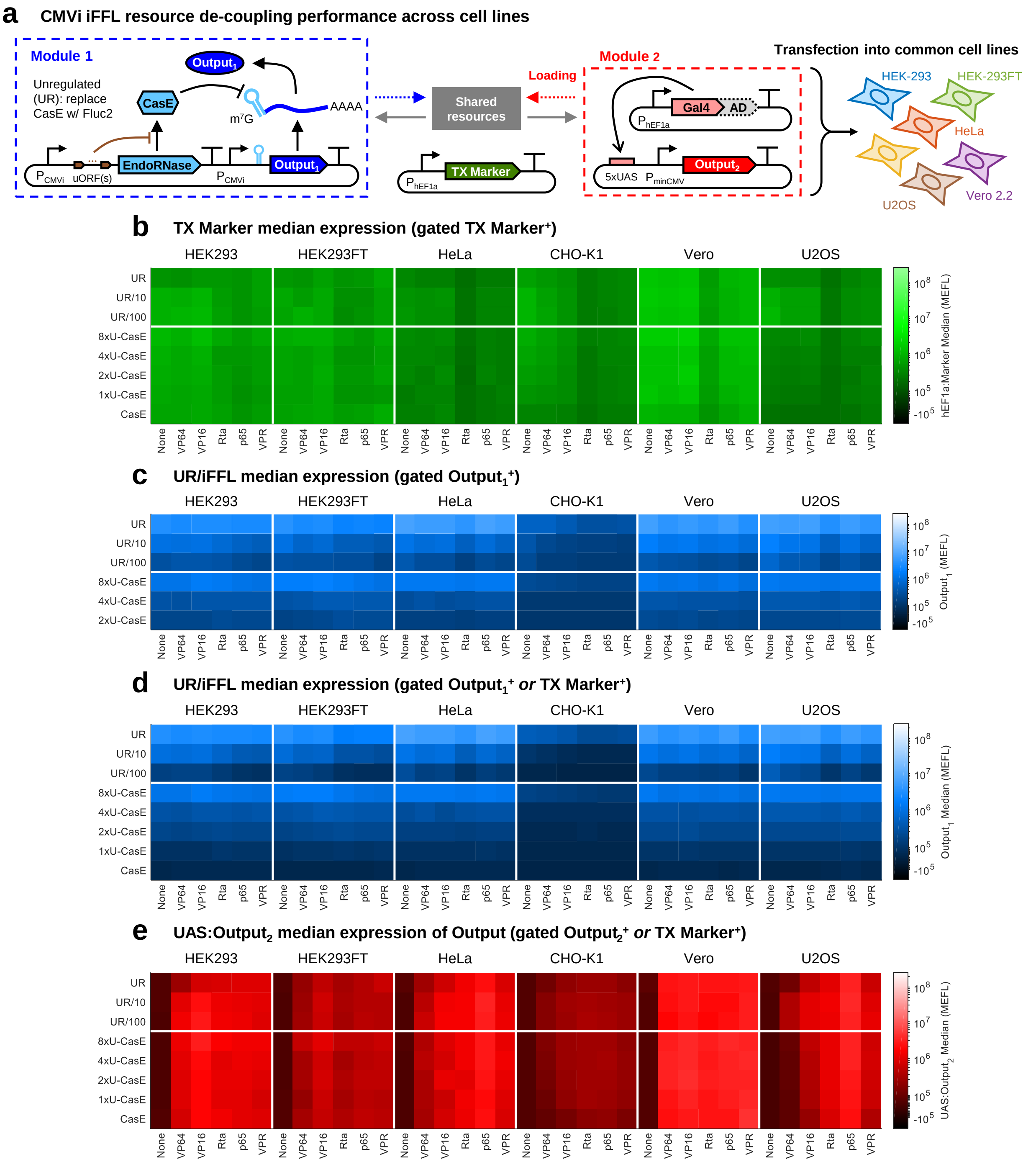

Supplementary Figure 21: Expression of each reporter in the experiment characterizing CMVi iFFL performance across cell lines. **(a)** Re-printing of the genetic diagram from Figure 5a. **(b-e)** Median expression levels of each fluorescent reporter for the data in Figure 5. (b) TX Marker, (c) $Output_1$ measured in cells gated positive only for $Output_1$, (d) $Output_1$ measured in cells gated positive for either $Output_1$ or TX Marker, and (e) UAS:$Output_2$. The values shown are the mean of median measurements from three experimental repeats. Note that in Panel (d) we can also measure the median level of $Output_1$ in the samples transfected with CasE or 1xU-CasE because of the gate on the TX Marker. These variants cannot be measured accurately when only gating on the output reporter because of their low expression near the gate threshold. Median values are provided in Source Data.

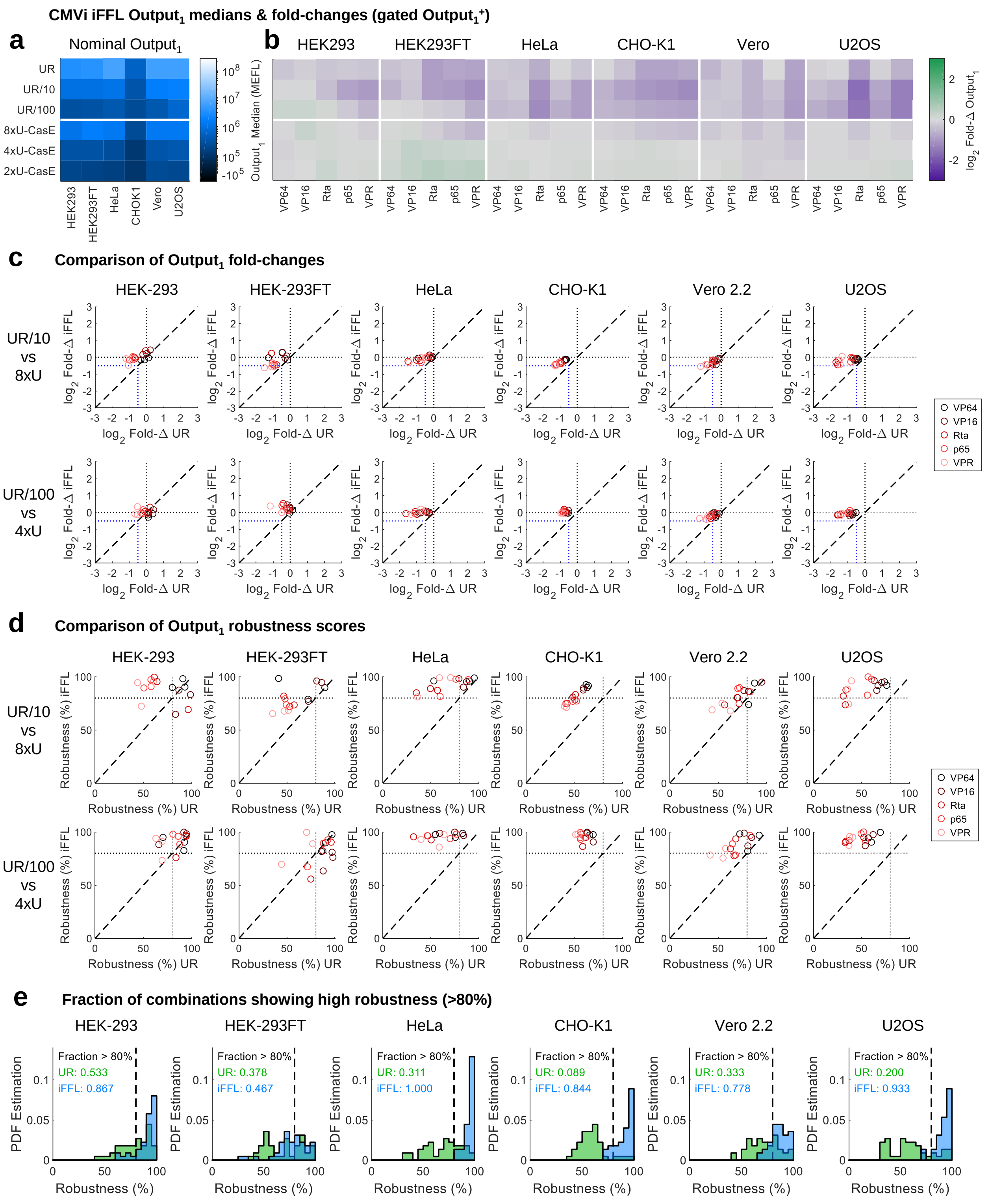

Supplementary Figure 22: Fold-changes and robustness of the CMVi iFFL in different cell lines when gating only on the output. **(a-c)** Reproduced from Figure 5b-d for convenience. Note that the axis limits of Panel (c) have been zoomed out to compare to other SI Figures. **(d)** Comparison of robustness scores for Output$_1$ expression in response to different Gal4 TAs in each cell line, between UR and iFFL devices with similar nominal output levels. Measurements from each experimental repeat are shown separately. The dotted lines correspond to 80% robustness scores. **(e)** Distributions of robustness scores for all UR vs iFFL variants in each cell line. The fraction of robustness scores above 80% for each group are shown inset. Data from each replicate are pooled together.

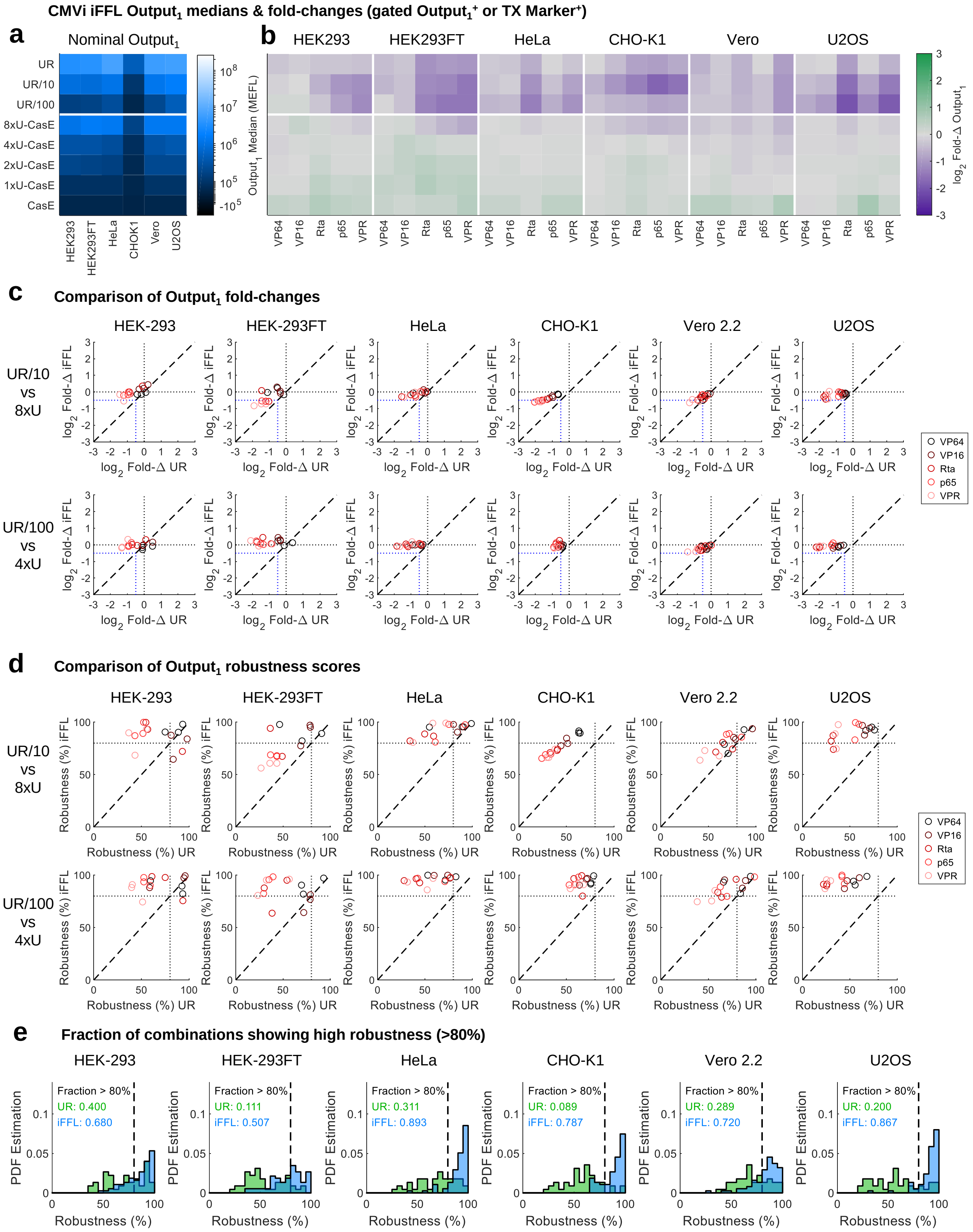

Supplementary Figure 23: Fold-changes and robustness of the CMVi iFFL in different cell lines when gating also for the TX Marker. **(a-e)** The same data as in Figure 22, but with all calculations done with cells gated positive for either $Output_1$ or the TX Marker, rather than just those positive for $Output_1$. Note that here we can also measure the median level of the samples transfected with CasE with zero or one uORFS (CasE, 1xU-CasE, respectively) because of the gate on the TX Marker.

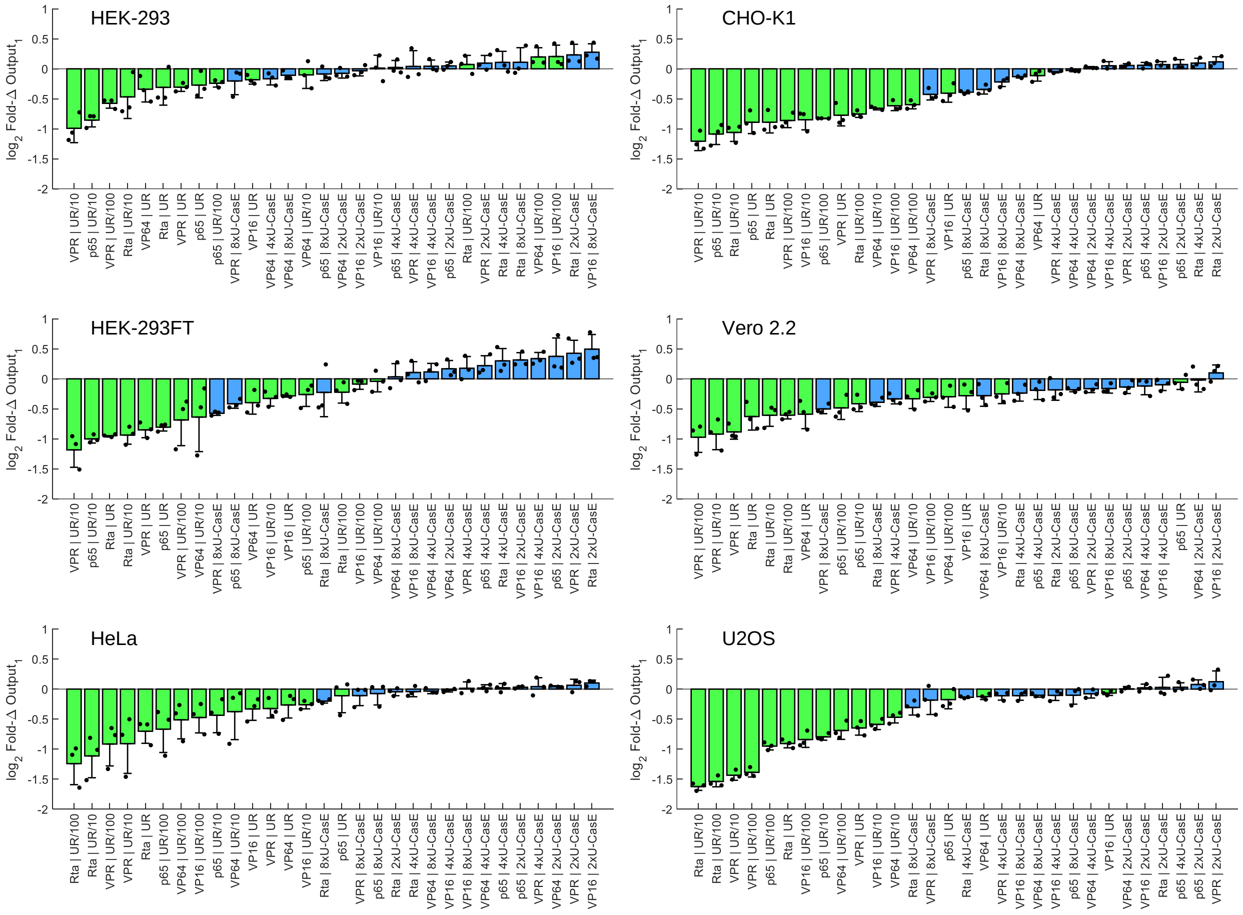

Supplementary Figure 24: All CMVi iFFL and UR output fold-changes, ranked per cell line. This is another representation of the data in Figure 5c. The $\log_2$ Fold-Δs are ranked in each cell line from most negatively to most positively affected. The plots show the mean ± standard deviation of three experimental repeats (represented by the individual points). Outputs which are minimally affected by resource loading by Gal4 TAs have $\log_2$ Fold-Δs near zero.

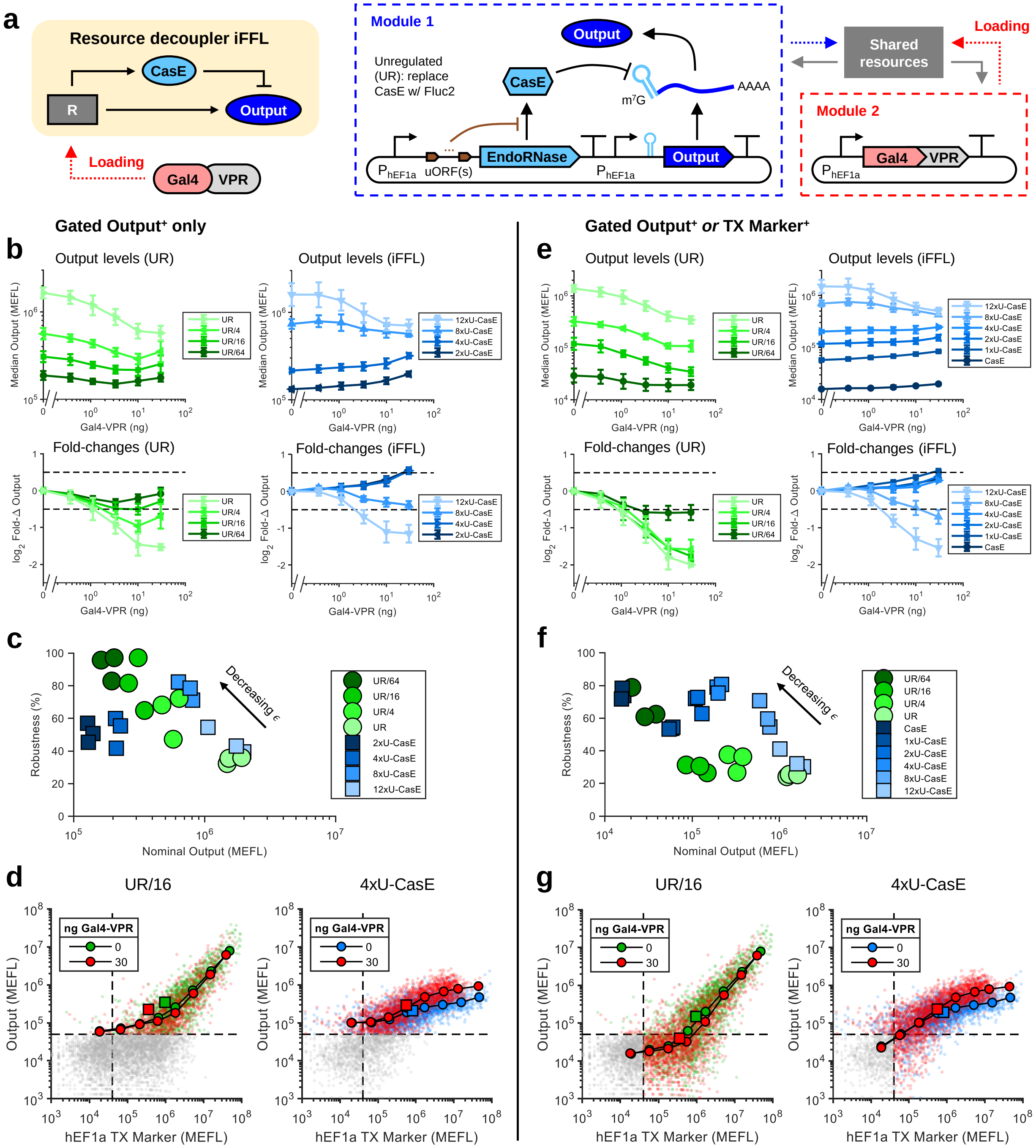

Supplementary Figure 25: Robustness of the hEF1a iFFL output level to resource loading by Gal4-VPR. **(a)** Genetic model system. The experiment was similar to that shown in Figure 4, with the CMVi promoters in Module 1 replaced by hEF1a promoters. The Output reporter is mNeonGreen, and not shown is the constitutive TX Marker (hEF1a:TagBFP). **(b-g)** Analogous analysis and calculations as in Supplementary Figure 19, with cells gated (b-d) positive for Output or (e-g) positive for either Output or TX Marker. Note that in Panels (b-c), we cannot accurately measure the median level of Output$_1$ in the iFFL variants CasE and 1xU-CasE because of low expression of Outpu$_1$ near the gate boundary. The errorbars represent the mean ± s.d. of measurements from three experimental repeats. To facilitate better comparability among plots, each sample was sub-sampled with the same number of cells (n = 3,000). Median values for both gating strategies are provided in Source Data.

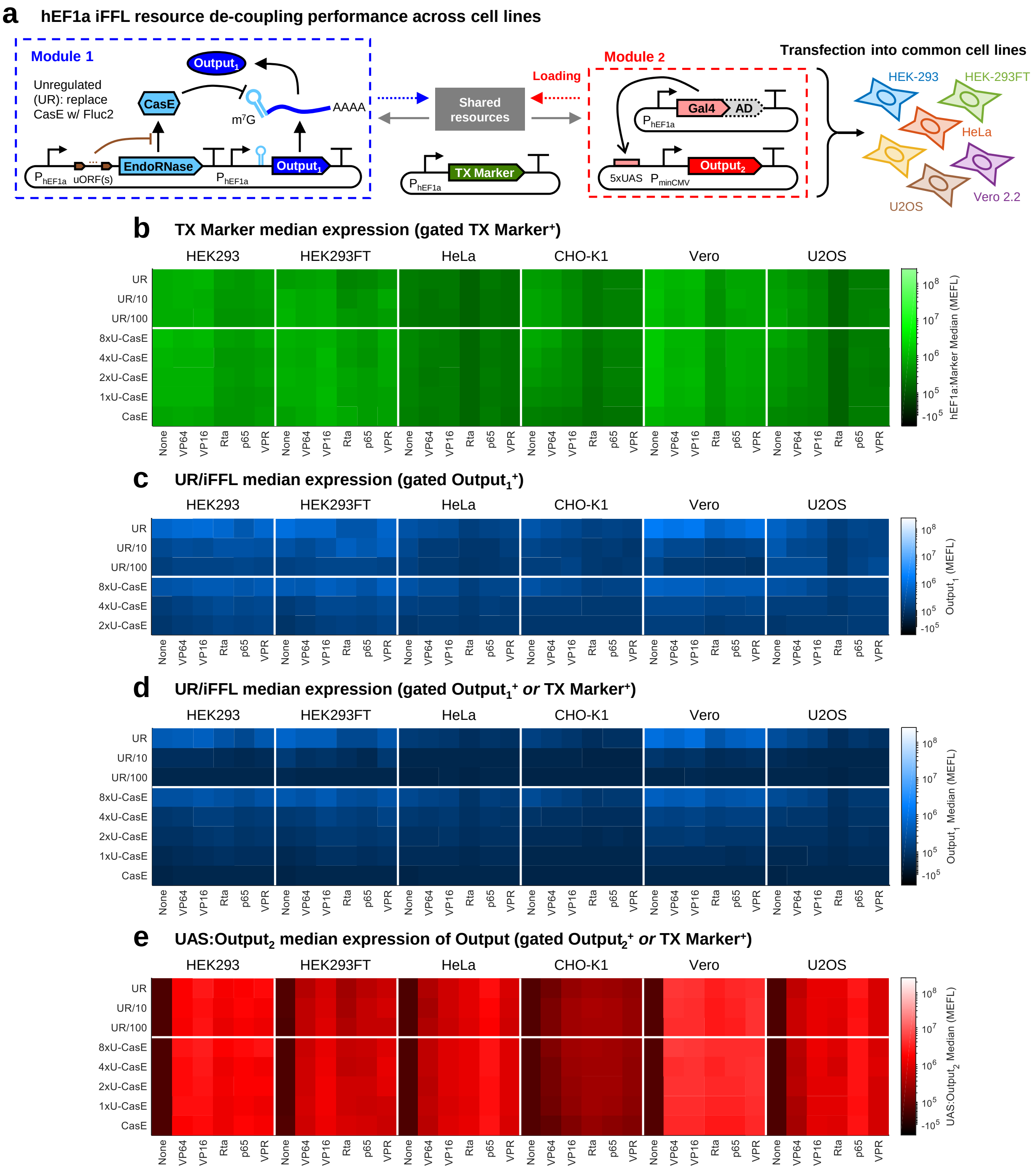

Supplementary Figure 26: Expression of each reporter in the experiment characterizing hEF1a iFFL performance across cell lines. **(a)** The genetic diagram of the iFFL and competitor modules (analogous to Figure 5a with the CMVi promoters in Module 1 replaced with hEF1a promoters). The module reporters are: Output$_1$: mNeonGreen, Output$_2$: mKO2, TX Marker: TagBFP. **(b-e)** Median expression levels of (b) TX Marker, (c) Output$_1$ measured in cells gated positive only for Output$_1$, (d) Output$_1$ measured in cells gated positive for either Output$_1$ or TX Marker, and (e) UAS:Output$_2$. The values shown are the mean of median measurements from three experimental repeats. Note that in Panel (c), we cannot accurately measure the median level of Output$_1$ in the iFFL variants CasE and 1xU-CasE because of low expression of Outpu$_1$ near the gate boundary. Median values are provided in Source Data.

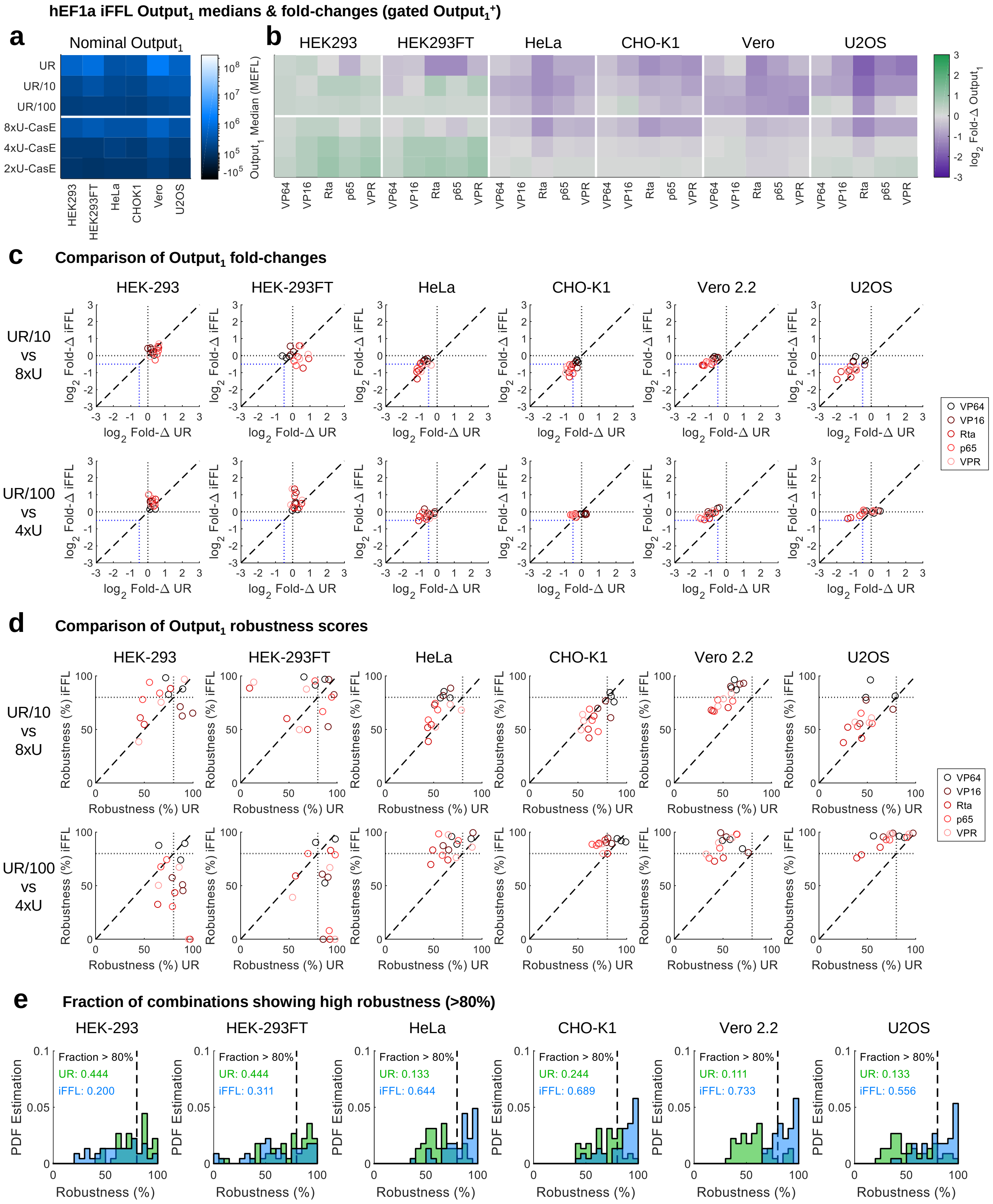

Supplementary Figure 27: Fold-changes and robustness of the hEF1a iFFL in different cell lines when gating only on the output. **(a-e)** Analogous plots to Figure 22 for the hEF1a iFFL instead of the CMVi iFFL.

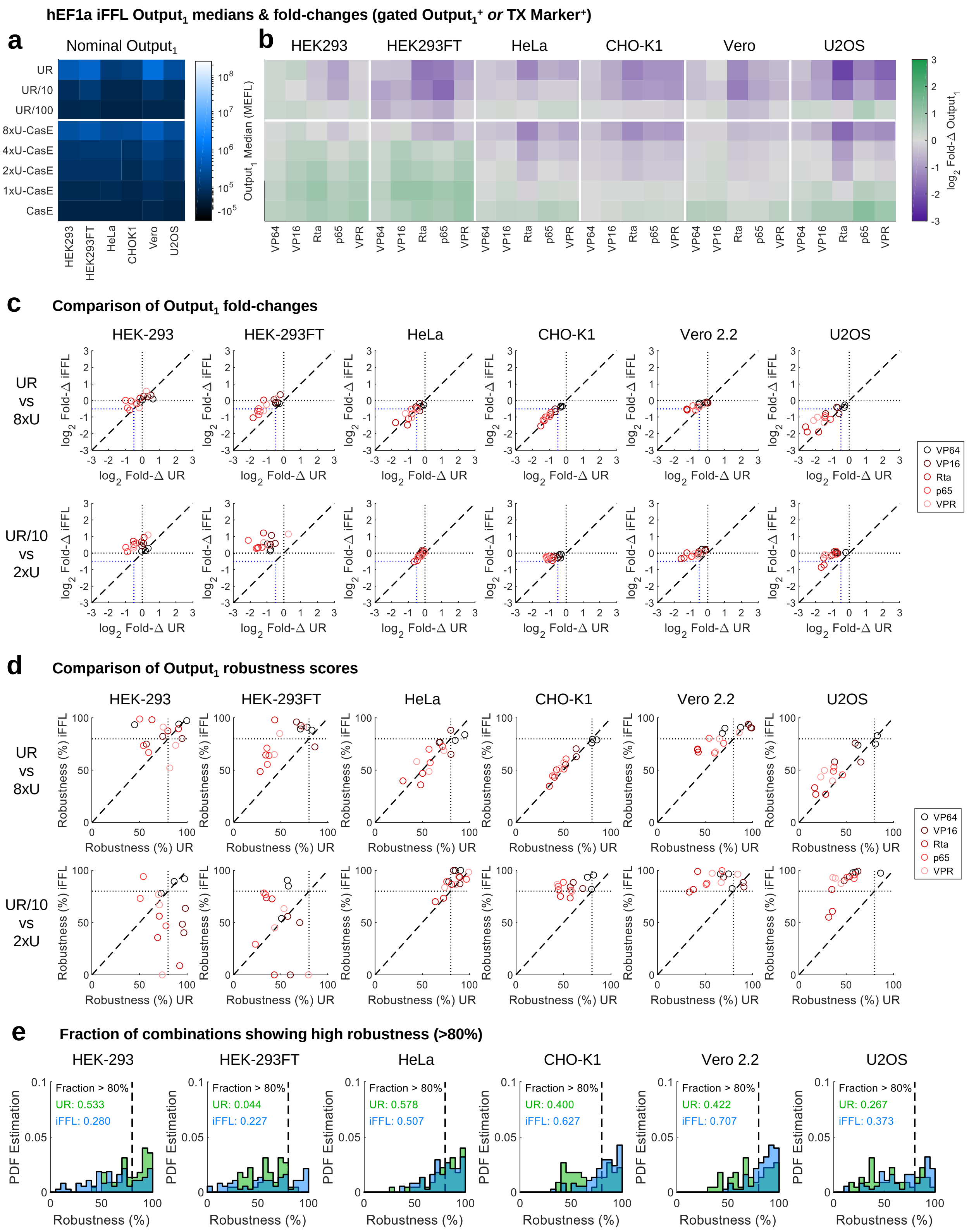

Supplementary Figure 28: Fold-changes and robustness of the hEF1a iFFL in different cell lines when gating also for the TX Marker. **(a-e)** Analogous plots to Figure 23 for the hEF1a iFFL instead of the CMVi iFFL.

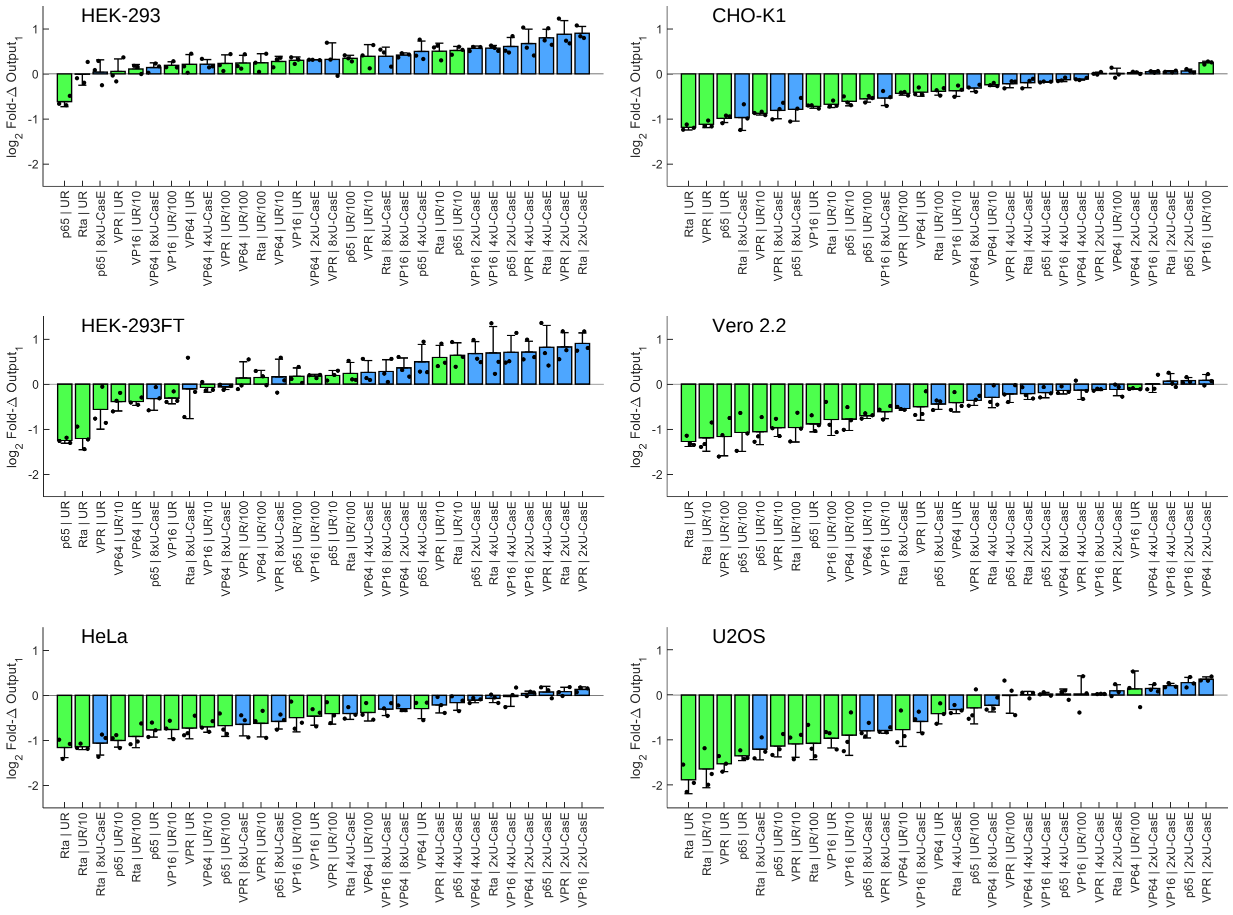

Supplementary Figure 29: All hEF1a iFFL and UR output fold-changes, ranked per cell line. This is another representation of the data in Supplementary Figure 27b. The $\log_2$ Fold-Δs are ranked in each cell line from most negatively to most positively affected. Errorbars represent the mean ± s.d. of measurements from three experimental repeats (represented by the individual points). Outputs that are minimally affected by resource loading by Gal4 TAs have $\log_2$ Fold-Δs near zero.

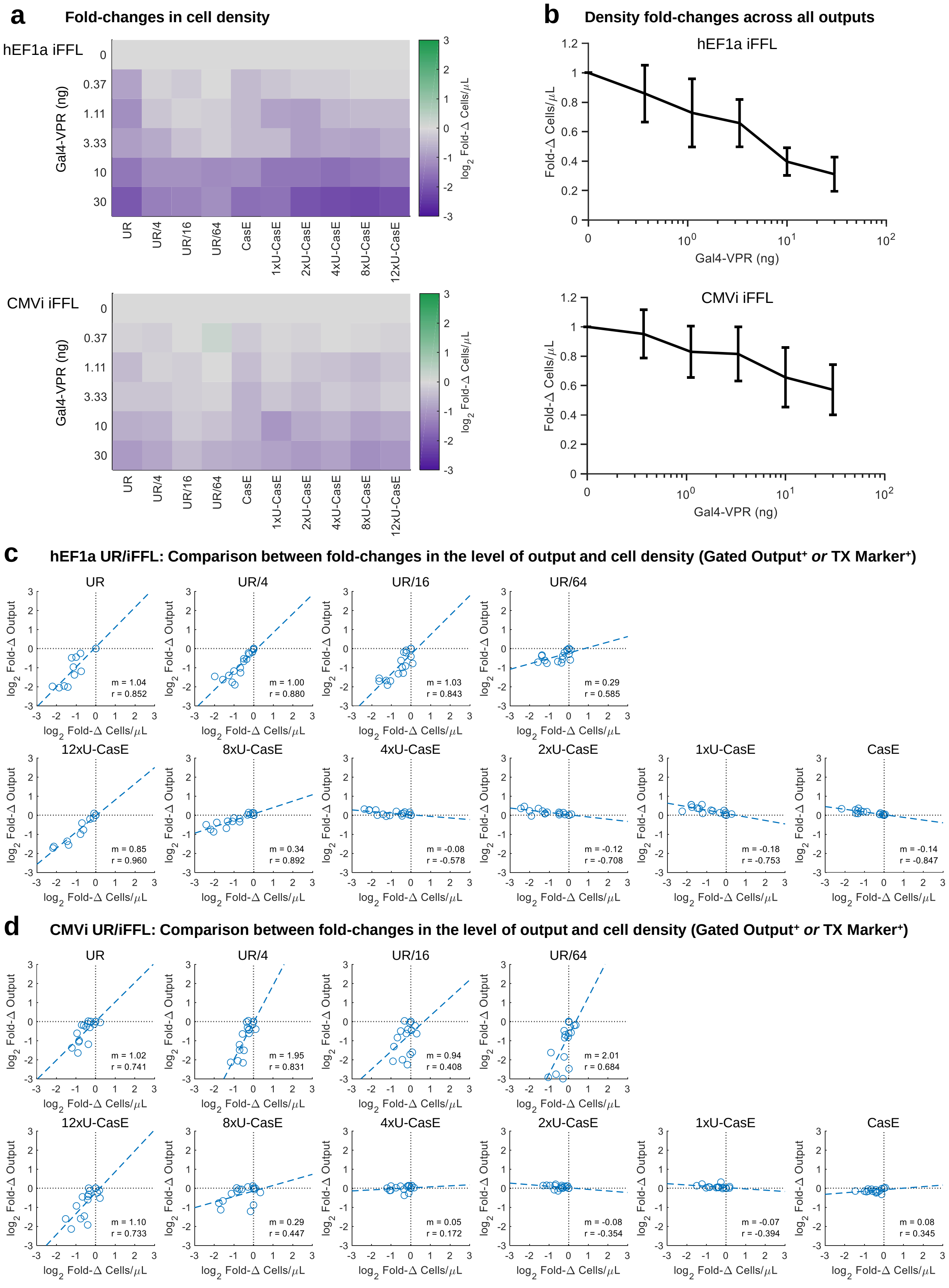

Supplementary Figure 30: Effect of Gal4 TAs on the concentration of cells measured with flow cytometry.

Supplementary Figure 30: (Previous page.) **(a)** A comparison of the Fold-Δs in concentration of cells measured in samples with the hEF1a and CMVi iFFLs and UR controls co-transfected with various concentrations of Gal4-VPR (Figure 4 & Supplementary Figure 25). Fold-Δs were computed by dividing the concentration of cells in each sample by the concentration of cells in the corresponding sample with the same iFFL or UR variant but co-transfected with 0 ng Gal4-VPR. The values represent the mean of Fold-Δs for three experimental repeats. **(b)** Mean ± standard deviation of the Fold-Δ in cell concentration at each dosage of Gal4-VPR, computed separately for both the hEF1a and CMVi iFFLs. Each point collates data from all UR/iFFL devices and three experimental repeats. **(c-d)** Correlation between Fold-Δs in cell concentration and output expression in response to Gal4-VPR for the hEF1a (c) or CMVi (d) UR and iFFL devices. Measurements from three experimental repeats are shown individually. Output levels are measured on cells gated positive for either the output or the TX Marker, corresponding to the data shown in Supplementary Figures 25e. m: slope, r: Pearson's correlation.

a **Fits & full distributions for each iFFL variant & Gal4-VPR titration level**

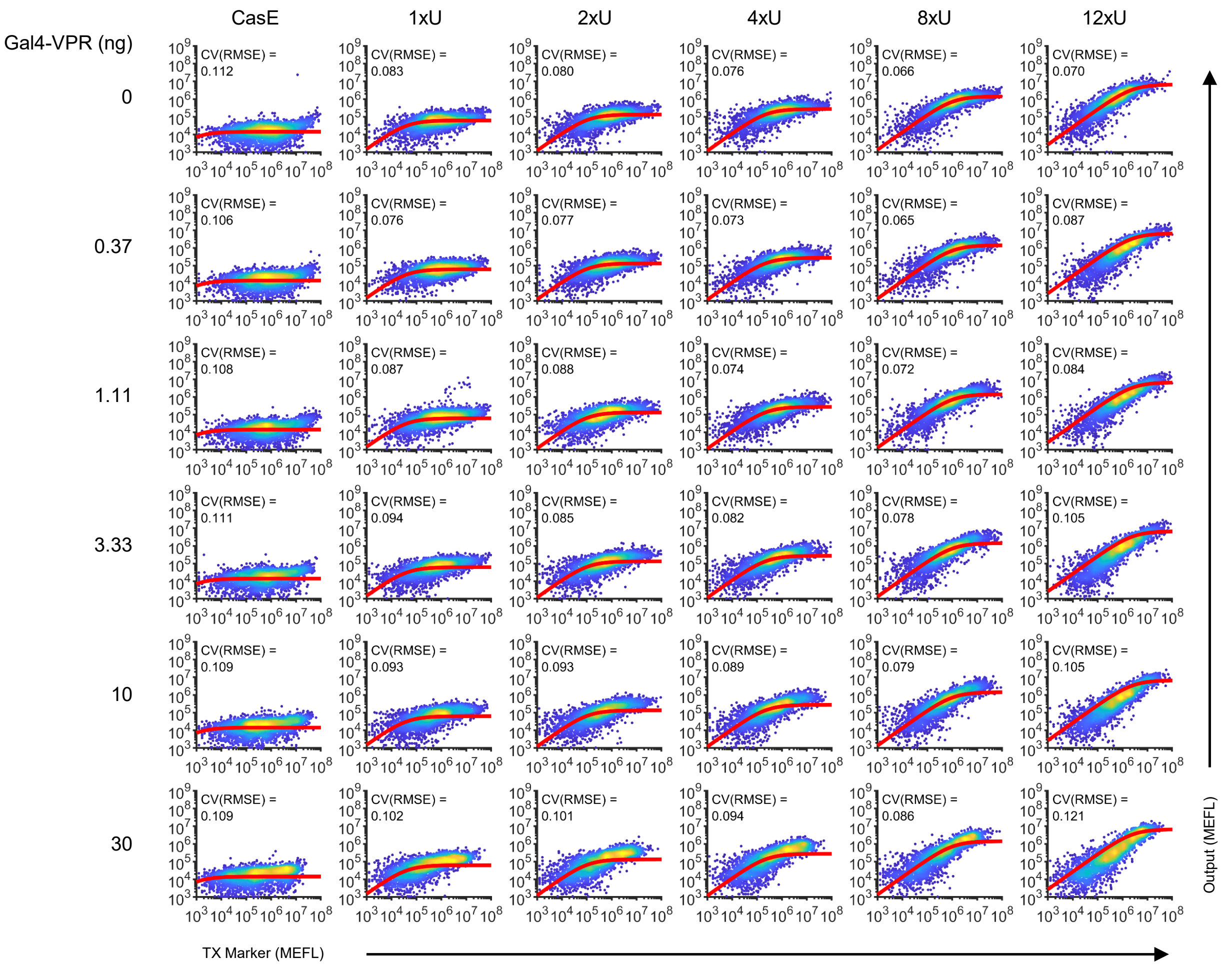

b **Effect of Gal4-VPR on fit parameters**

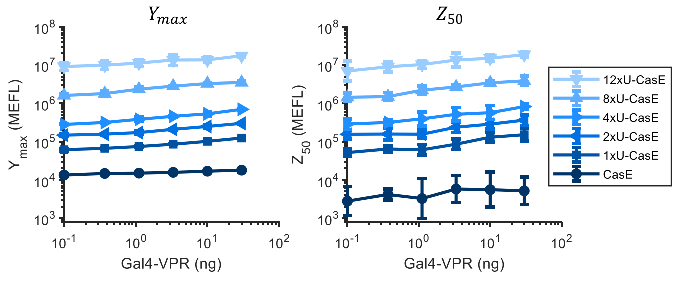

Supplementary Figure 31: Effect of Gal4-VPR resource loading on iFFL model parameters. **(a)** Fits of the iFFL model overlaid with scatterplots of cells at various Gal4-VPR DNA dosages and for different variants of the hEF1a iFFL. The data are representative and taken from the first experimental replicate of the data shown in Supplementary Figure 25. The cells are colored by density. The CV(RMSE) is the root-mean-square error between the model and data, normalized by the mean of the data ($\log_{10}$-transformed first since the cell-to-cell variance is approximately log-normally distributed). To facilitate better comparability among plots, each sample was sub-sampled with the same number of cells (n = 3,000). **(b)** Measurements of model parameters as a function of Gal4-VPR DNA dosage. Plotted values are the mean (μ) ± relative error ($\frac{1}{ln(10)} \cdot \frac{\sigma}{\mu}$) of parameter values for three experimental repeats. Fit parameters are provided in Source Data.

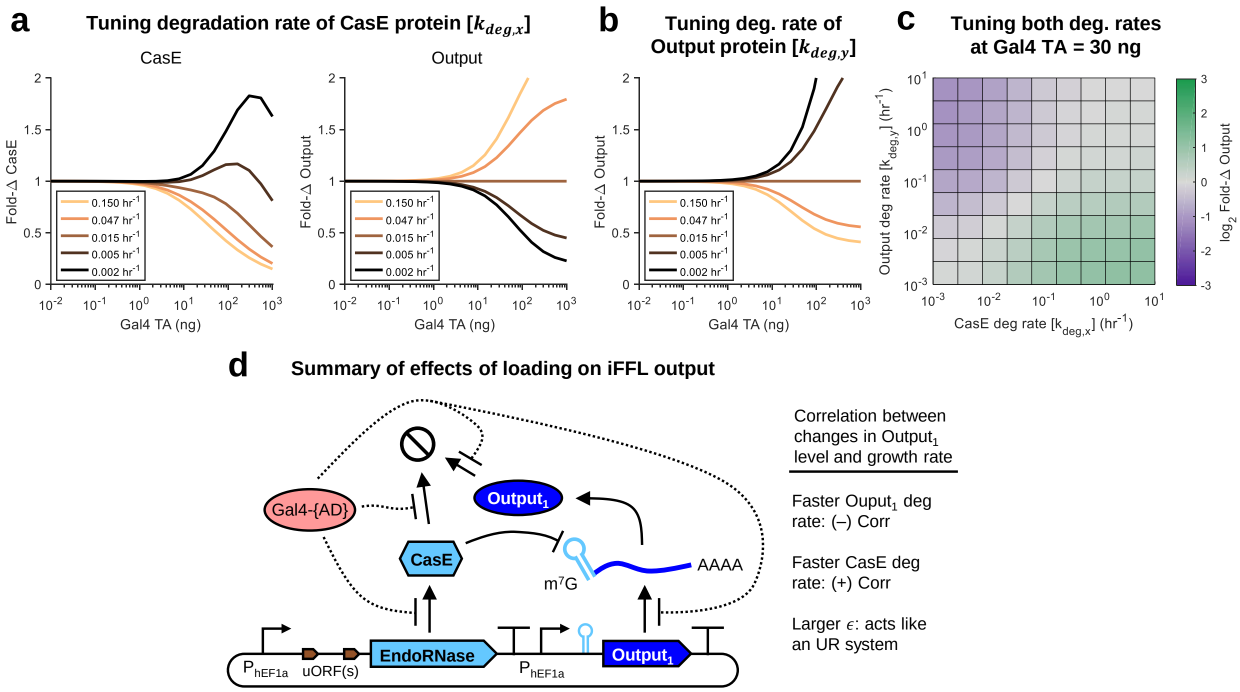

Supplementary Figure 32: Combined effects of growth inhibition and resource loading on non-target gene expression levels. **(a)** Simulation of changes in the levels of CasE and output in an iFFL module in response to theoretical Gal4 TA(s) in which CasE has varying degradation rates [$k_{deg,x}$]. The Fold-Δ indicates the fold-change in the Output relative to its nominal value when Gal4 DNA = 0. The values are taken at steady-state. **(b)** Simulations as in Panel (a) but for cases where the output protein has varying degradation rates [$k_{deg,y}$]. **(c)** The combined effects of changing the degradation rates of the output protein and CasE on the qualitative effect of loading on the output expression level. Here the Gal4 DNA level is fixed at 30 ng. The colormap represents fold changes in output levels relative to the nominal level in the absence of loading. **(d)** Schematic summarizing the effects of resource loading and growth inhibition by Gal4 TAs on the level of iFFL output. If the degradation rate of Output_1 is faster or slower than that of CasE, then the level of Output_1 goes down or up in response to a TA, respectively. These changes can then cause a negative or a positive correlation between changes in the level of Output_1 and cell density. As ϵ increases, the system acts more like a UR device (see Supplementary Figures 15-16). Simulation parameters are provided in Source Data.

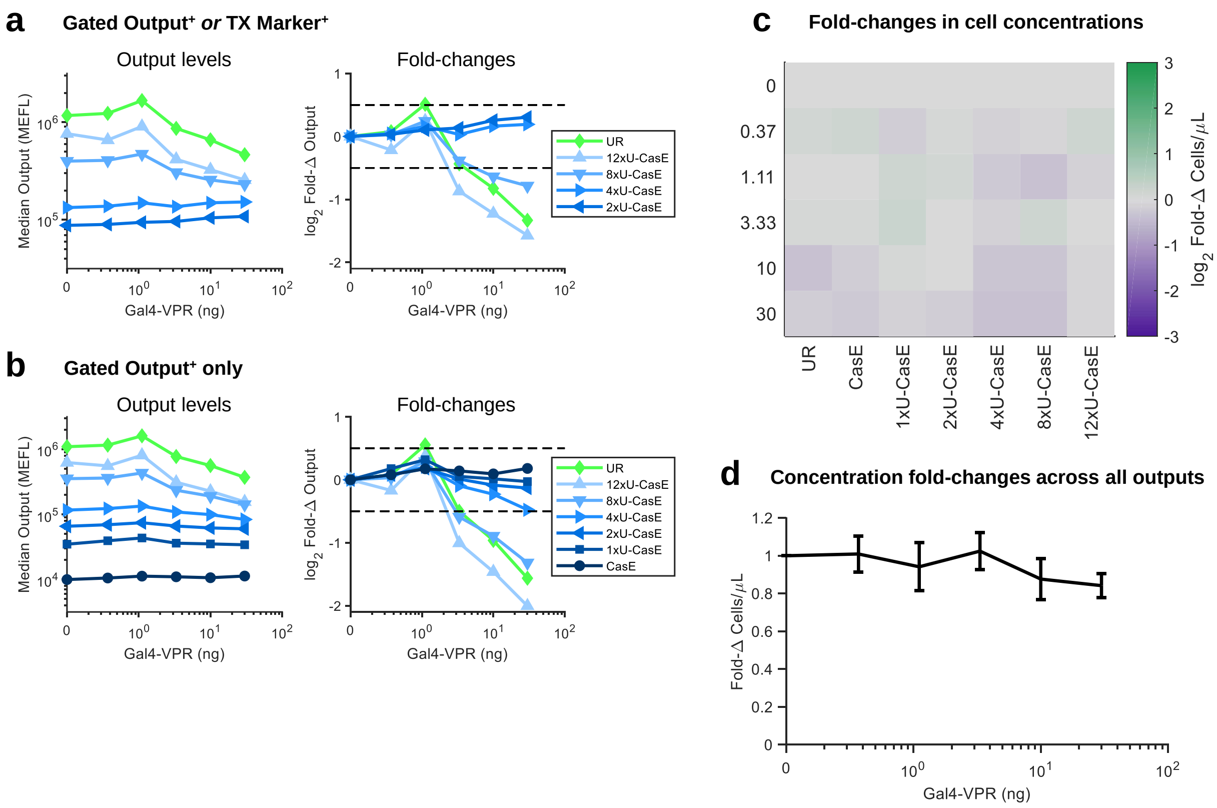

Supplementary Figure 33: Response of hEF1a iFFL to Gal4-VPR resource loading when transfected with a less toxic reagent. **(a-b)** Output levels and Fold-Δs for hEF1a iFFL variants and an UR control (undiluted) using the same plasmids, experimental design, and calculations as in Supplementary Figure 25b/e, with the transfection reagent changed from Lipofectamine 3000 to Viafect. Cells were gated (a) positive for output or (b) positive for either Output or TX Marker. Note that in Panel (a), we cannot accurately measure the median level of $Output_1$ in the iFFL variants CasE and 1xU-CasE because of low expression of $Output_1$ near the gate boundary. **(c)** A comparison of the Fold-Δs in concentration of cells measured in samples co-transfected with various concentrations of Gal4-VPR. Fold-Δs were computed by dividing the concentration of cells in each sample by the concentration of cells in the corresponding sample with the same iFFL or UR variant but co-transfected with 0 ng Gal4-VPR. **(d)** Mean ± standard deviation of the Fold-Δ in cell concentration at each dosage of Gal4-VPR. Each point collates data from all UR/iFFL devices for one experimental repeat. Median values are provided in Source Data.

Comparison of CMVi iFFL median expression & set points across cell lines

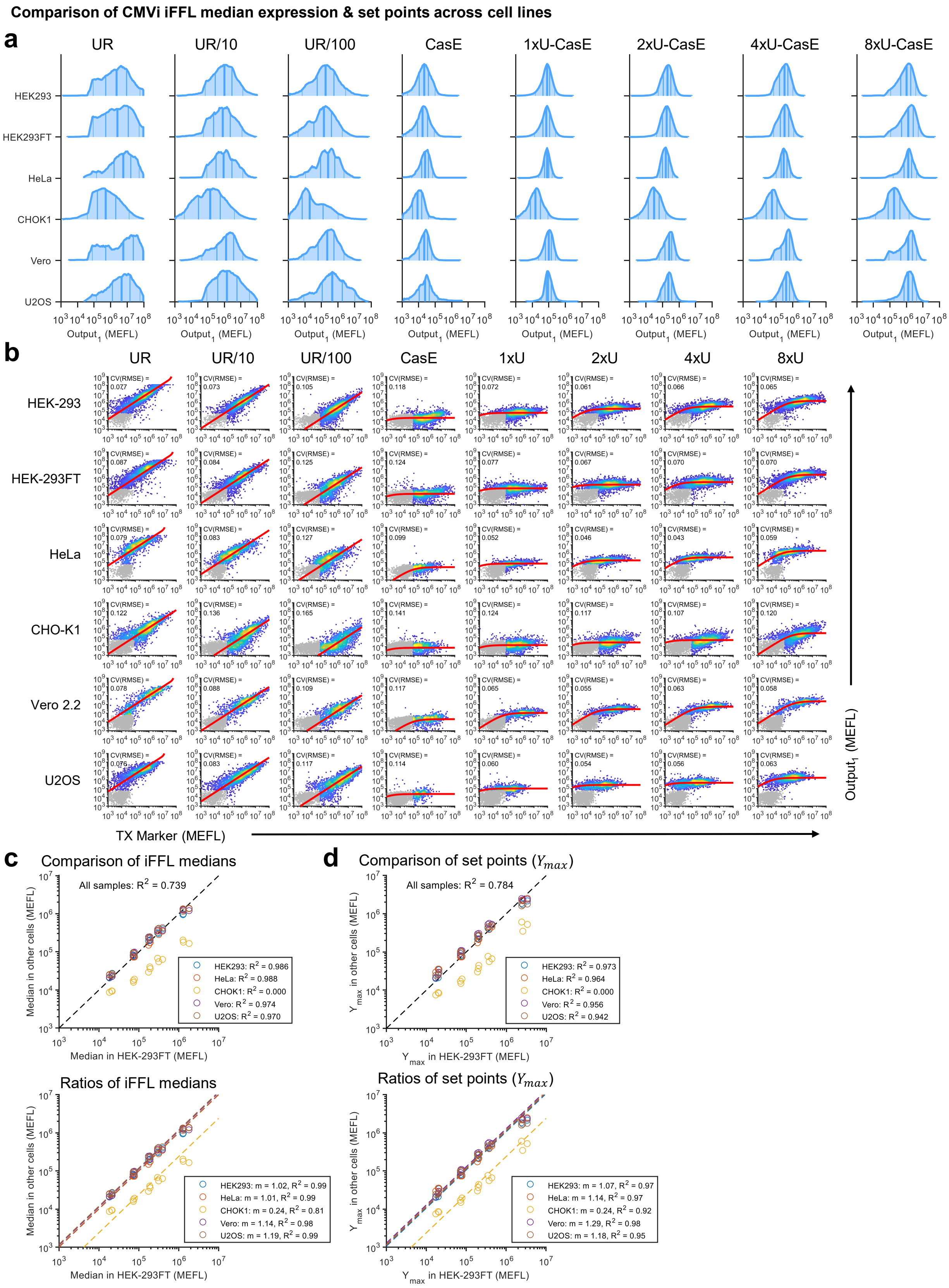

Supplementary Figure 34: Comparison of CMVi iFFL output levels across cell lines.

Supplementary Figure 34: (Previous page.) **(a)** Histograms showing the distribution of the iFFL output ($Output_1$) for the samples in Figure 23a (co-transfected with Gal4-None (Gal4 DBD only) and gated positive for either $Output_1$ or TX Marker). The lines on the histograms denote the 5th, 25th, 50th, 75th, and 95th percentiles. The 50th percentiles correspond to the medians shown in Figure 23a. Data are representative and taken from the third experimental repeat. **(b)** Fits of the iFFL model to TX Marker vs $Output_1$ distributions. Note that the relatively high expression from the CMVi promoter compared to the hEF1a promoter in most cell lines throws off the fitting method. To remedy this, only data gated positive for either $Output_1$ or the TX Marker (shown colored by density – rather than the entire distribution) were used for fitting. For UR plasmids, the output is proportional to the TX Marker, so we fit with a simple linear formula: Output = m · TX Marker. The CV(RMSE) is the root-mean-square error between the model and non-binned data, normalized by the mean of the data ($\log_{10}$-transformed first since the cell-to-cell variance is approximately log-normally distributed). Data are representative from the third experimental repeat. To facilitate better comparability between plots, each sample was sub-sampled with the same number of cells (n = 3,000). **(c-d)** Comparison of the median level of $Output_1$ (c) and fit value of the model parameter $Y_{\max}$ (d) between each cell line and HEK-293FT cells (which were used in most experiments). The top plot computes R^2 values based on a 1:1 ratio between the values in each cell line. The lower plot find the overall ratio (m) between each cell line and HEK-293FT cells by averaging the ratios across individual samples, then uses the ratio to compute the R^2 values. Measurements from each experimental repeat are shown separately. The ~4-fold lower median $Output_1$ and $Y_{\max}$ measured in CHO-K1 cells may partially be explained by the relatively lower CMVi expression in CHO-K1 cells (Figure 2). In addition, seeing as the UR samples also saw a decrease compared to other cell lines that was larger in magnitude than expected based on the data in Figure 2, the disparity may be explained by a CHO-K1 cell-specific increase in mRNA degradation rate and/or decrease in translation rate for CMVi-transcribed mRNAs bearing the CasE target site hairpin. We know that the specific fluorescent reporter (EYFP) used for $Output_1$ is stable in CHO-K1 cells because it was used as $Output_2$ in the experiment shown in Supplementary Figure 11, and was not substantially lower than in other cell lines tested. Median values, fit parameters, and the number of cells plotted per histogram are provided in Source Data.

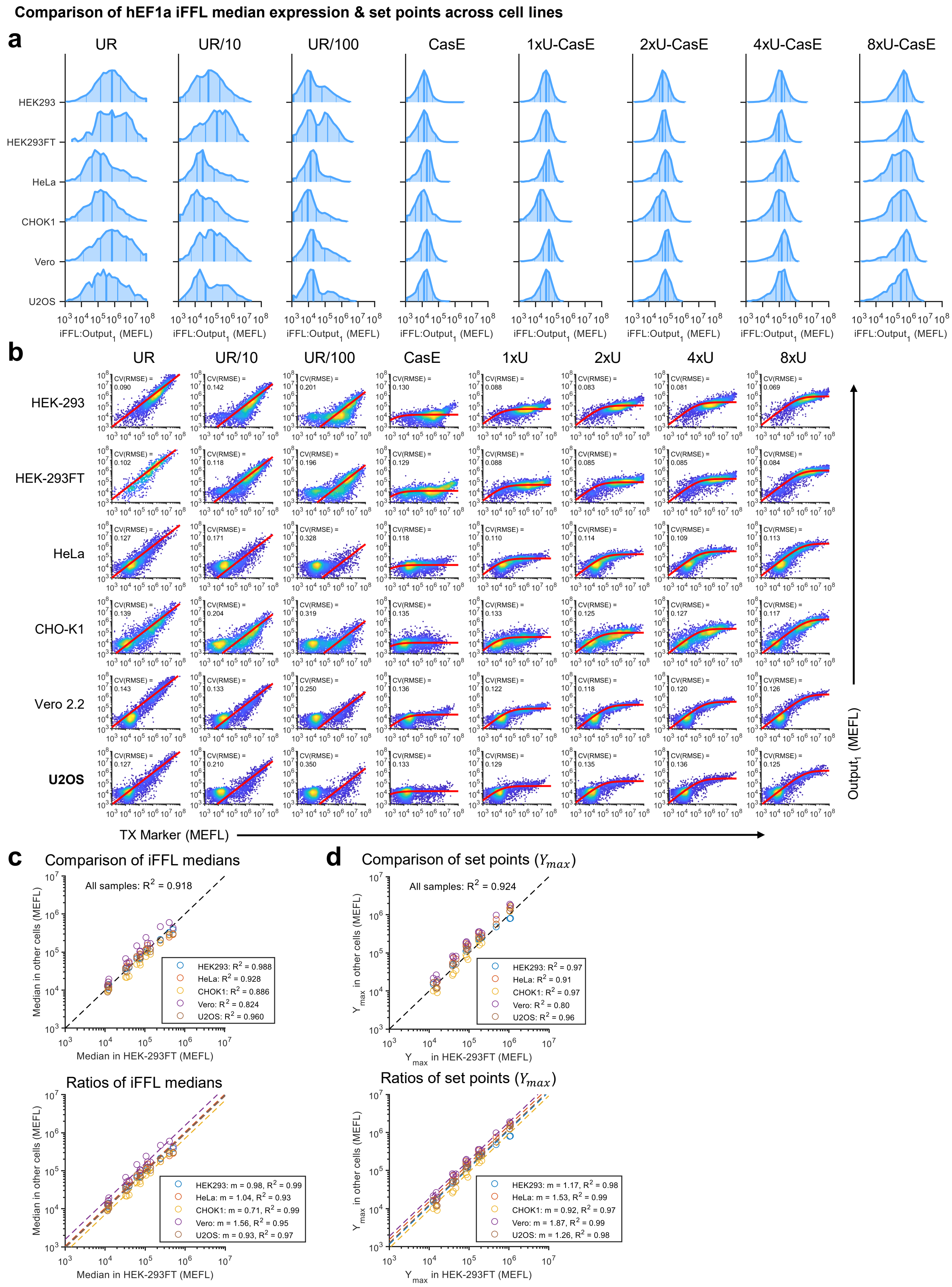

Supplementary Figure 35: Comparison of hEF1a iFFL output levels across cell lines.

Supplementary Figure 35: (Previous page.) **(a)** Histograms showing the distribution of the iFFL output ($Output_1$) for the samples in Figure 28a (co-transfected with Gal4-None (Gal4 DBD only) and gated positive for either $Output_1$ or TX Marker). The lines on the histograms denote the 5th, 25th, 50th, 75th, and 95th percentiles. The 50th percentiles correspond to the medians shown in Figure 28a. Data are representative and taken from the first experimental repeat. **(b)** Fits of the iFFL model to TX Marker vs $Output_1$ distributions. Note that the relatively low transfection efficiency of some cell lines throws off the fitting in some cases. To remedy this, each sample was binned into half-log-decade width bins based on the levels of TX Marker and 100 cells from each bin were extracted and combined for fitting. Cells are colored by density. For UR plasmids, the output is proportional to the TX Marker, so we fit with a simple linear formula: Output = m · TX Marker. The CV(RMSE) is the root-mean-square error between the model and non-binned data, normalized by the mean of the data ($\log_{10}$-transformed first since the cell-to-cell variance is approximately log-normally distributed). Data are representative from the first experimental repeat. To facilitate better comparability between plots, each sample was sub-sampled with the same number of cells (n = 3,000). **(c-d)** Comparison of the median level of $Output_1$ (c) and fit value of the model parameter Y_{max} (d) between each cell line and HEK-293FT cells (which were used in most experiments). The top plot computes R^2 values based on a 1:1 ratio between the values in each cell line. The lower plot find the overall ratio (m) between each cell line and HEK-293FT cells by averaging the ratios across individual samples, then uses the ratio to compute the R^2 values. Measurements from each experimental repeat are shown separately. Median values, fit parameters, and the number of cells plotted per histogram are provided in Source Data.

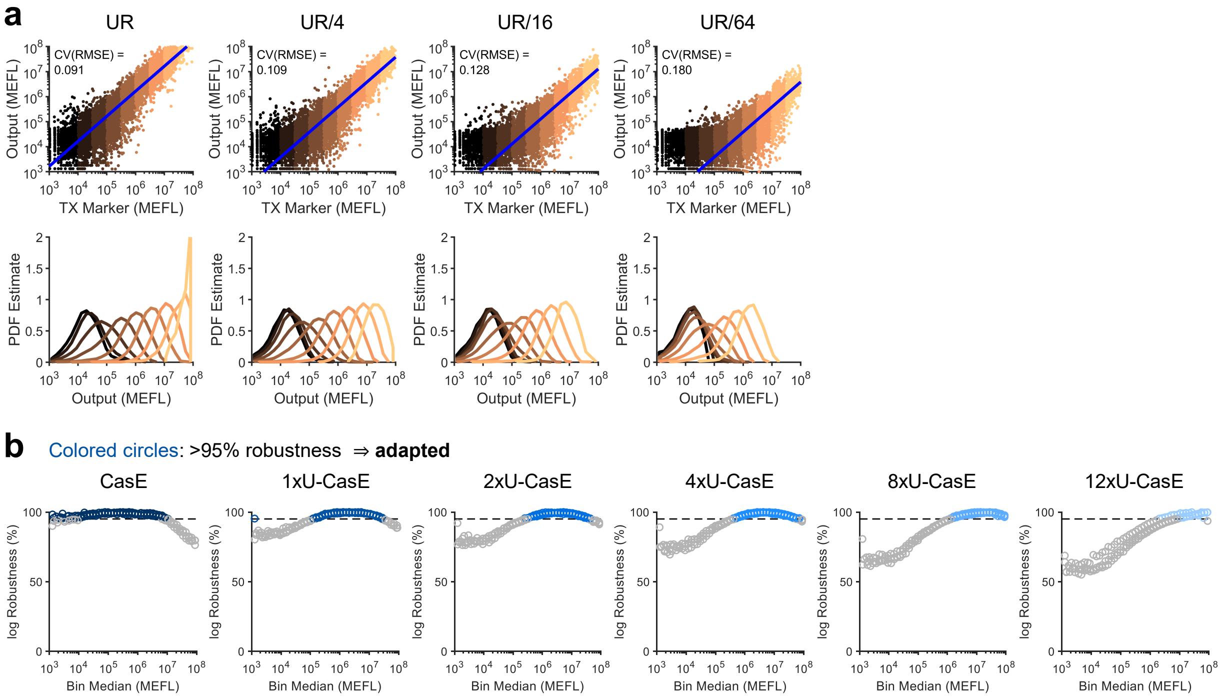

Supplementary Figure 36: Adaptation to plasmid DNA copy number by the CMVi iFFL. **(a)** Scatterplots and histograms of the same form described in Figure 4b, showing here the remaining hEF1a UR controls with diluted plasmid copy numbers. For UR plasmids, the output is proportional to the TX Marker, so we fit with a simple linear formula: Output = m · TX Marker. Data are representative and taken from the first experimental repeat. To facilitate better comparability among plots, each bin was sub-sampled with the same number of cells (n = 3,000). **(b)** Robustness of iFFL Output levels in more finely-sampled bins, computed in reference to the fit parameter Y_{max}. The values were log-transformed before the calculation. Bins with robustness scores over 95% (which we define as adapted to DNA copy number) are highlighted in shades of blue. Individual experimental repeats are shown separately. Fit parameters for iFFL samples are provided in Source Data.

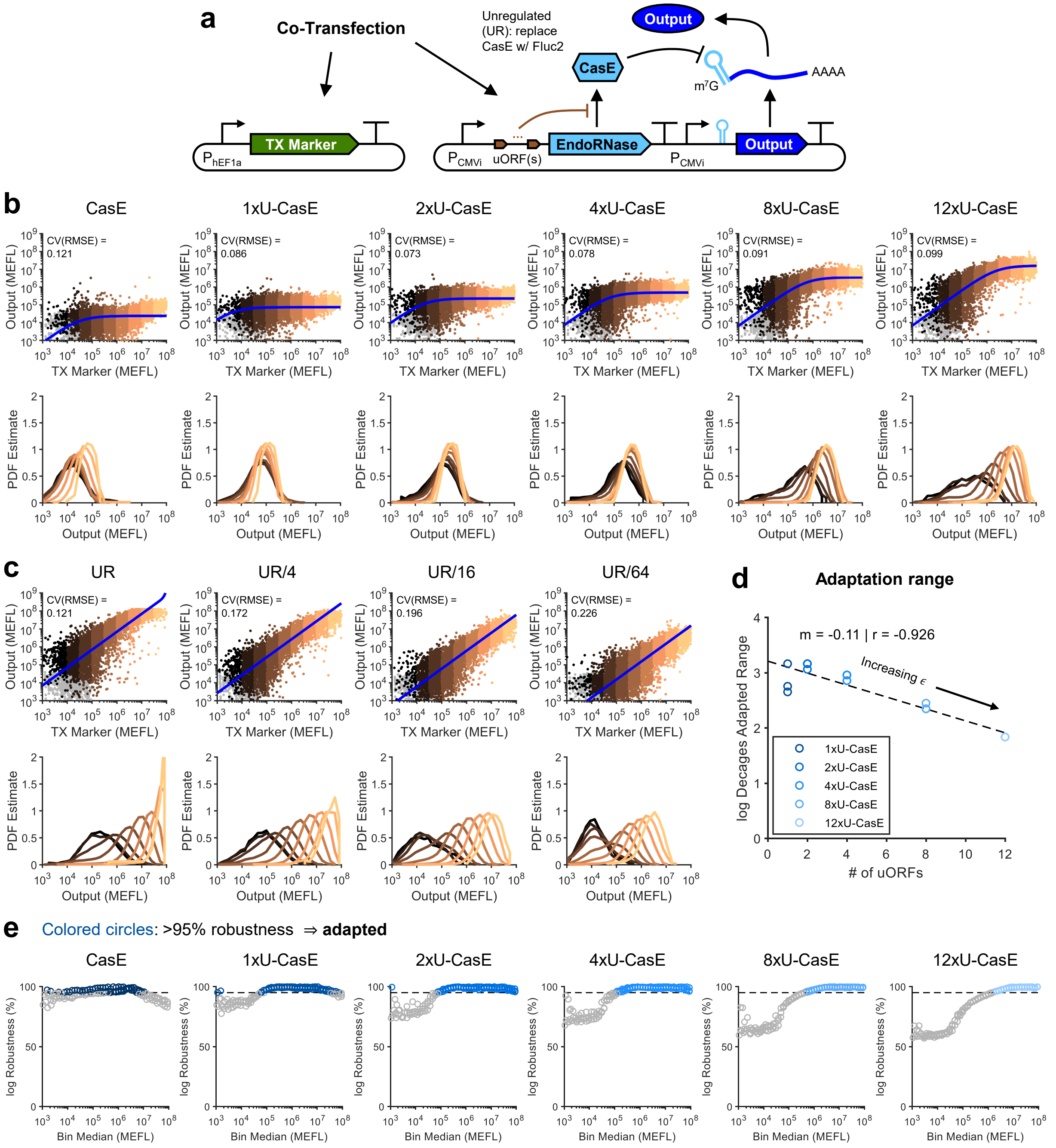

Supplementary Figure 37: Adaptation to plasmid DNA copy number by the CMVi iFFL. **(a)** Genetic diagram of the CMVi iFFL and a constitutive TX Marker to report plasmid dosage delivered to each cell. The reporters are: Output: EYFP, TX Marker: mKO2. **(b-e)** Analogous plots to those shown in Figure 4b-c and Supplementary Figure 36, but for the CMVi iFFL. Similarly to the hEF1a iFFL, a hEF1a TX Marker was also co-transfected with the CMVi iFFL. For the fits in Panels (b) & (e), the relatively high expression from the CMVi promoter compared to the hEF1a promoter in HEK-293FT cells throws off the fitting method. To remedy this, only data gated positive for either the output or a second CMVi-driven TX Marker (shown in black/copper-colored dots – rather than the entire distribution) were used for fitting. For Panels (b-c), the data are representative and taken from the first experimental repeat. For Panels (d-e), individual experimental repeats are shown separately. To facilitate better comparability among plots, each bin was sub-sampled with the same number of cells (n = 3,000). Fit parameters for iFFL samples are provided in Source Data.

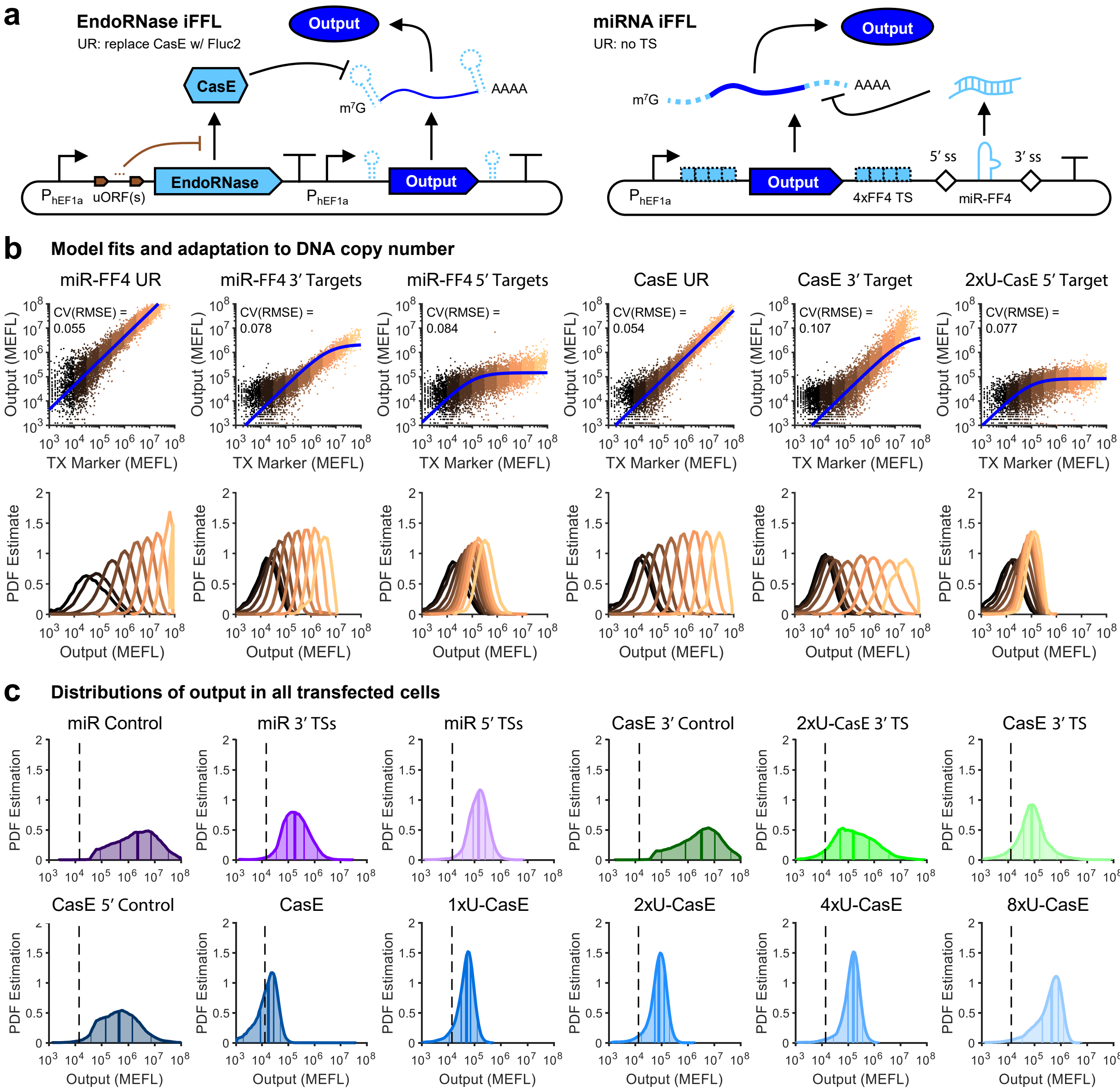

Supplementary Figure 38: Comparison of CasE and miR-FF4 target site locations. **(a)** Schematics of the CasE and miR-FF4 iFFLs with target sites in the 5' or 3' untranslated regions (UTRs). The CasE UR control replaces CasE with Fluc2 and has a 5' or 3' target site for CasE. The miR-FF4 UR control has no target sites but still expresses miR-FF4 from its intron. The output reporter is mNeonGreen. Not shown is the TX Marker (hEF1a:TagBFP). **(b)** Top row: TX Marker vs Output levels for select samples, overlaid with fits of the iFFL model. The CV(RMSE) is the root-mean-square error between the model and non-binned data, normalized by the mean of the data ($\log_{10}$-transformed first since the cell-to-cell variance is approximately log-normally distributed). Bottom row: histograms of the Output levels for cells within each color-coded bin (as indicated in the scatters). CasE UR refers to the control with a 5' target site. **(c)** Distributions of output levels in transfected cells (gated positive for either Output or TX Marker) for each miR and endoRNase variant tested. Samples were measured by flow cytometry 72 hours post-transfection in HEK-293FT cells. Median values, fit parameters, and the number of cells plotted per histogram are provided in Source Data.

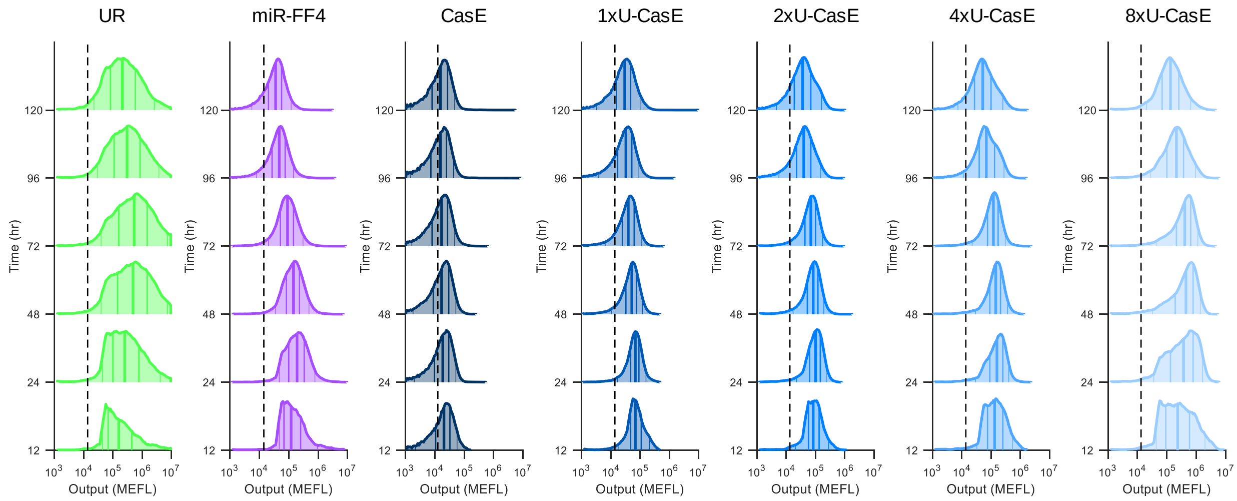

Supplementary Figure 39: Time-evolution of fluorescence distributions over time following transient transfection. Histograms showing the distribution of Output in transfected cells (gated positive for either Output or TX Marker) at the indicated time points. The lines on the histograms denote the 5th, 25th, 50th, 75th, and 95th percentiles. The 50th percentiles correspond to the medians shown in Figure 6e (left plot). The dashed black lines indicate the median output level for the untransfected cells (excluded from the 'transfected' gate) at the 12 hr timepoint. Median values for each sample at each time point and the number of cells plotted per histogram are provided in Source Data.

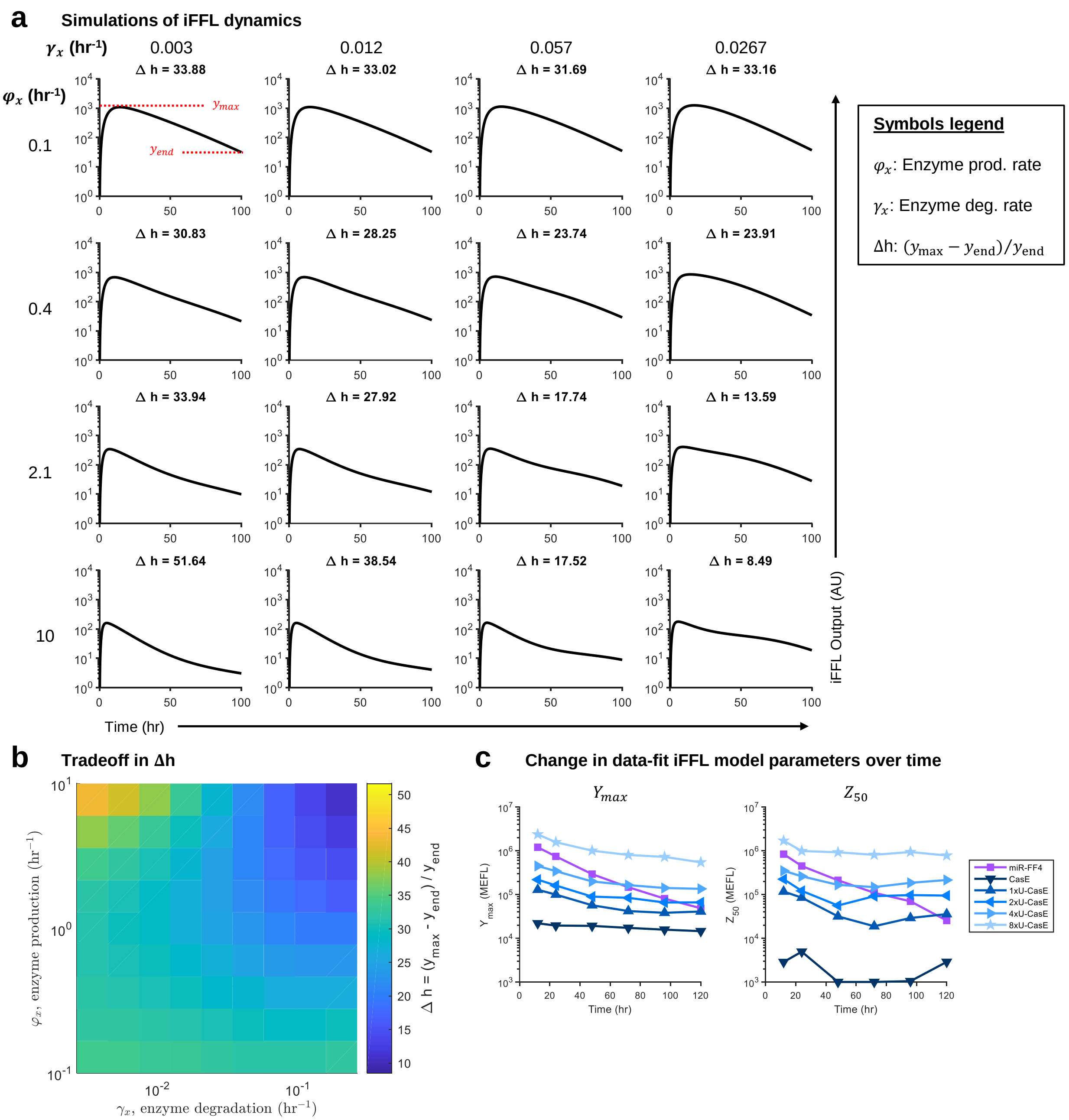

Supplementary Figure 40: ODE simulations of transient transfections of the CasE iFFL. (a) Simulated time-courses for the CasE iFFL output for varying CasE production rate (φ_x) and degradation rate (γ_x). Δh is defined as $\frac{y_{\max}-y_{\text{end}}}{y_{\text{end}}}$, where y_{max} is the maximum level of Output during the time-course and y_{end} is the level of Output at the final time point. **(b)** Comparison of how different combinations of values for φ_x and γ_x affect Δh. **(c)** Comparison of fit parameters $Y_{\max}$ and Z_{50} over time for the iFFL variants for the experiment shown in Figure 6e. Note that for the iFFL variant with no uORFS, Z_{50} is almost undetectable, so the value shown is not necessarily accurate. Simulation parameters and fit parameters for each sample and time point are provided in Source Data.

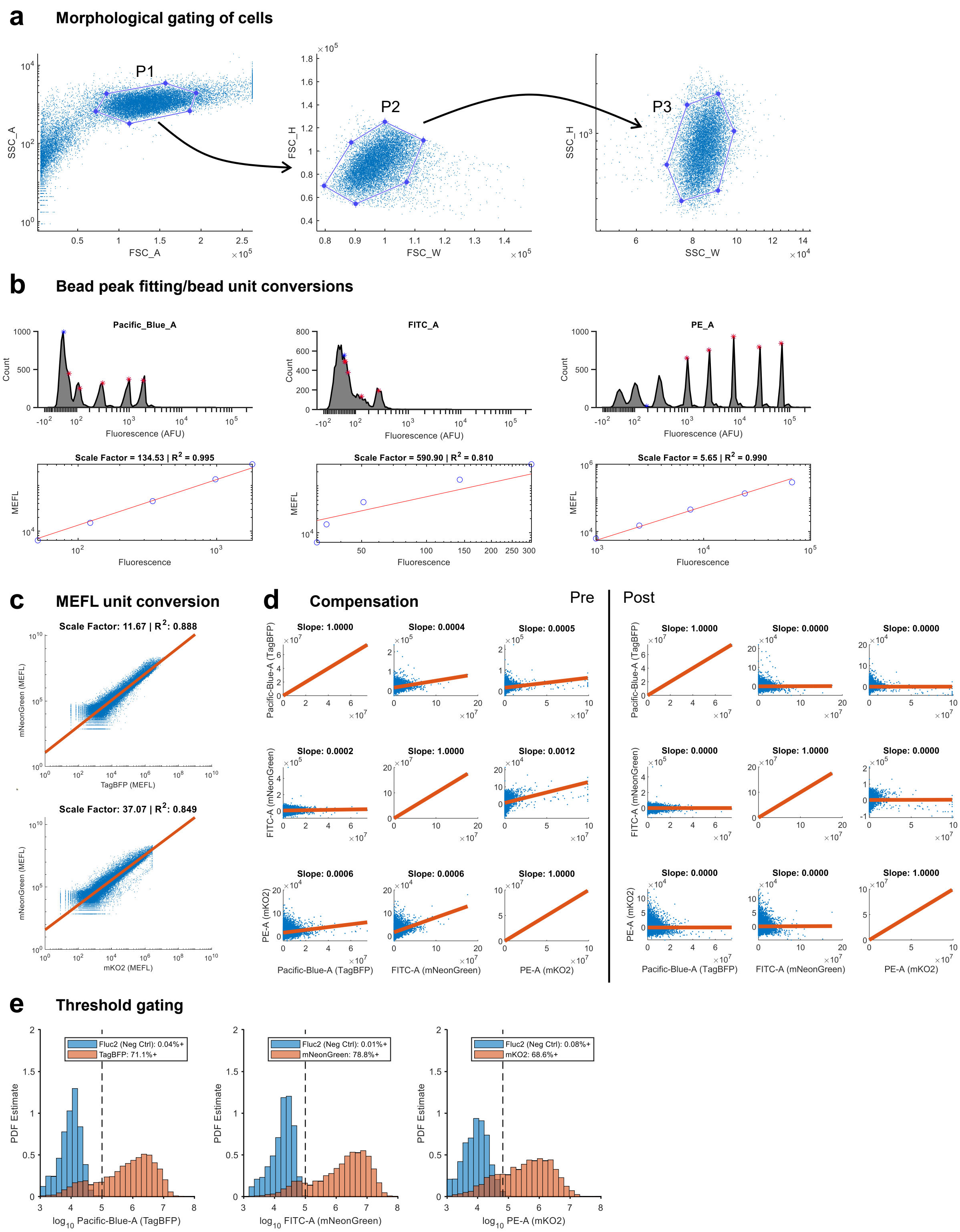

Supplementary Figure 41: Example flow data processing pipeline.

Supplementary Figure 41: (Previous page.) This example corresponds to the data in Supplementary Figure 4. **(a)** Morphological gates based on forward and side scatter (FSC, SSC). Gates P2 and P3 are gated on subsequent subpopulations coming from P1. **(b)** Spherotech RCP-30-5A rainbow calibration bead peak fitting scheme. Beads data are fit with an N-dimensional Gaussian mixture model (GMM) to find peaks. The top plots show the peaks for the three experimental channels; red asterisks indicate 'positive' peaks and blue asterisks indicate the 'negative' population. The bottom plots show the linear regression fit between the fluorescence values found for each peak (means of the GMM) and the expected molecules of equivalent fluorescein (MEFLs) in the given peak subpopulation. **(c)** Channel correction using multicolor controls. Cells are transfected with plasmids containing two transcription units, one expressing mNeonGreen (the FITC channel reporter) and the other expressing a reporter that is primarily in another channel. Both reporters are transcribed by the same promoter (CMV). A linear regression between the channels is used to compute a correction factor such that the same promoter driving different reporters measured in different channels yields the same output level as measured in MEFL units. **(d)** Fluorescence bleedthrough compensation. Cells are transfected with plasmids containing a single fluorescent reporter. A linear regression is fit between each pair of channels for each single-color control. The fit slopes of the linear regressions are used as coefficients in matrix-based spectral de-mixing. The plot group on the left shows the fit to the original control data. The plot group on the right shows a diagnostic plot where the compensated data are fit with a linear regression again. **(e)** Threshold gates for each channel. Threshold gates are drawn manually and aim to capture less than 1% of cells that do not express the reporter of interest. The plots show the percent of cells passing the gate from two control populations: (1) cells transfected with hEF1a:Fluc2 (and thus not expressing any fluorescent reporters, but still having been transfected the same as other cells), and (2) the single-color control samples (as described in Panel (d)).

Gate Name	M	M-B	M-Y	M-R	M-YR	M-BYR
Description	Morphological (FSC/SSC) gate	M <AND> TagBFP+ (UAS:Output$_2$)	M <AND> mNeonGreen+ (Marker)	M <AND> mKO2+ ({P}:Output$_1$)	M <AND> mNeonGreen+/ mKO2+	M <AND> All 3 colors positive

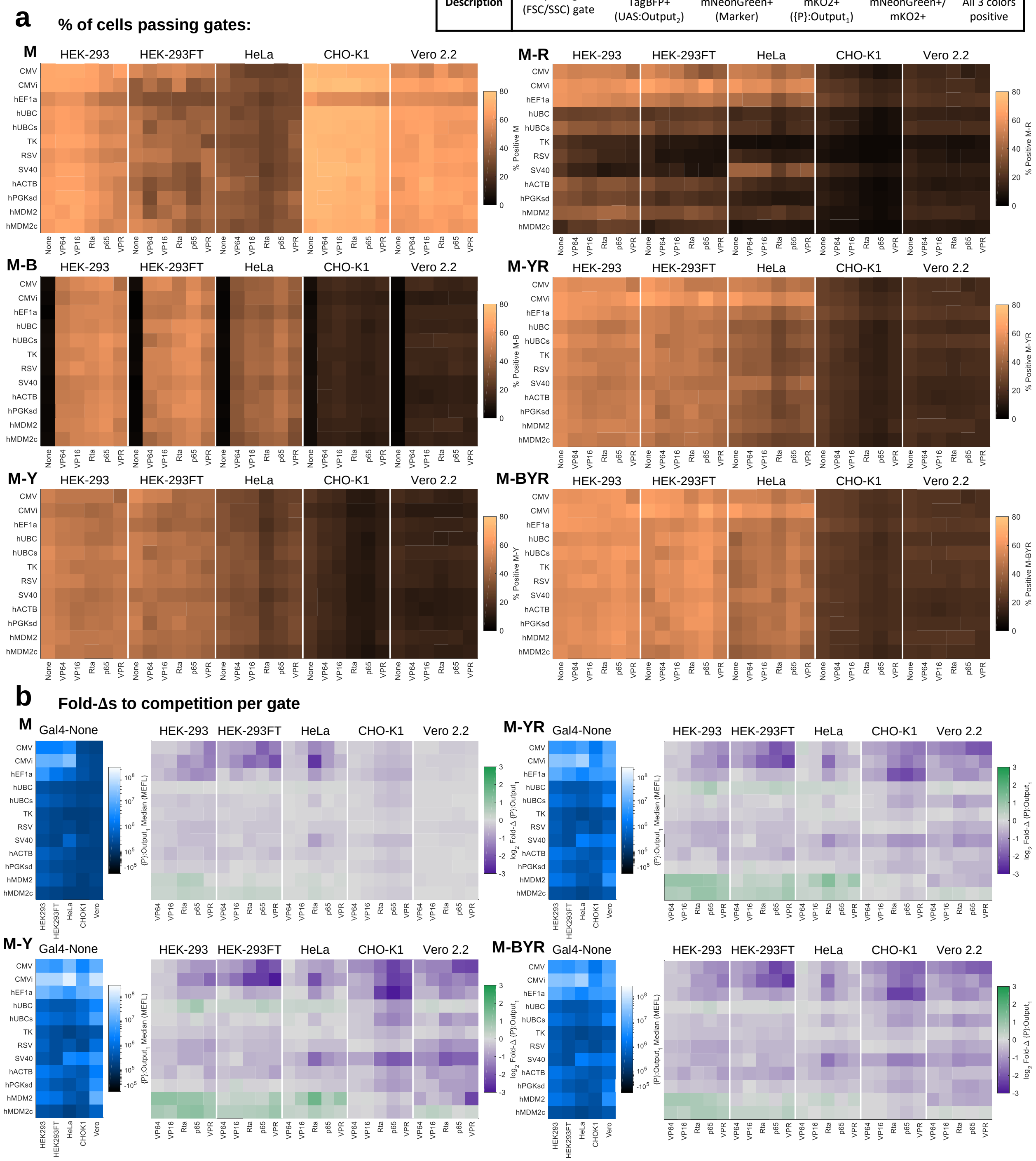

Supplementary Figure 42: Comparison of gating strategies on the measurement of fold-changes. **(a)** Percent of cells in each sample passing the indicated gates for the data in Figure 2 (see table in top-right for a description of the gates). Each percentage is the mean of measurements from three experimental repeats. **(b)** Comparison of Nominal Outputs and fold-changes (Fold-Δs) analogous to and computed in the same way as those shown in Figure 2b-c for a relevant subset of the different gating strategies in Panel (a). The M-YR sample is the same gating strategy as the data shown in Figure 2 before extra autofluorescence subtraction (Supplementary Figure 44). Median values for each gating strategy are provided in Source Data.

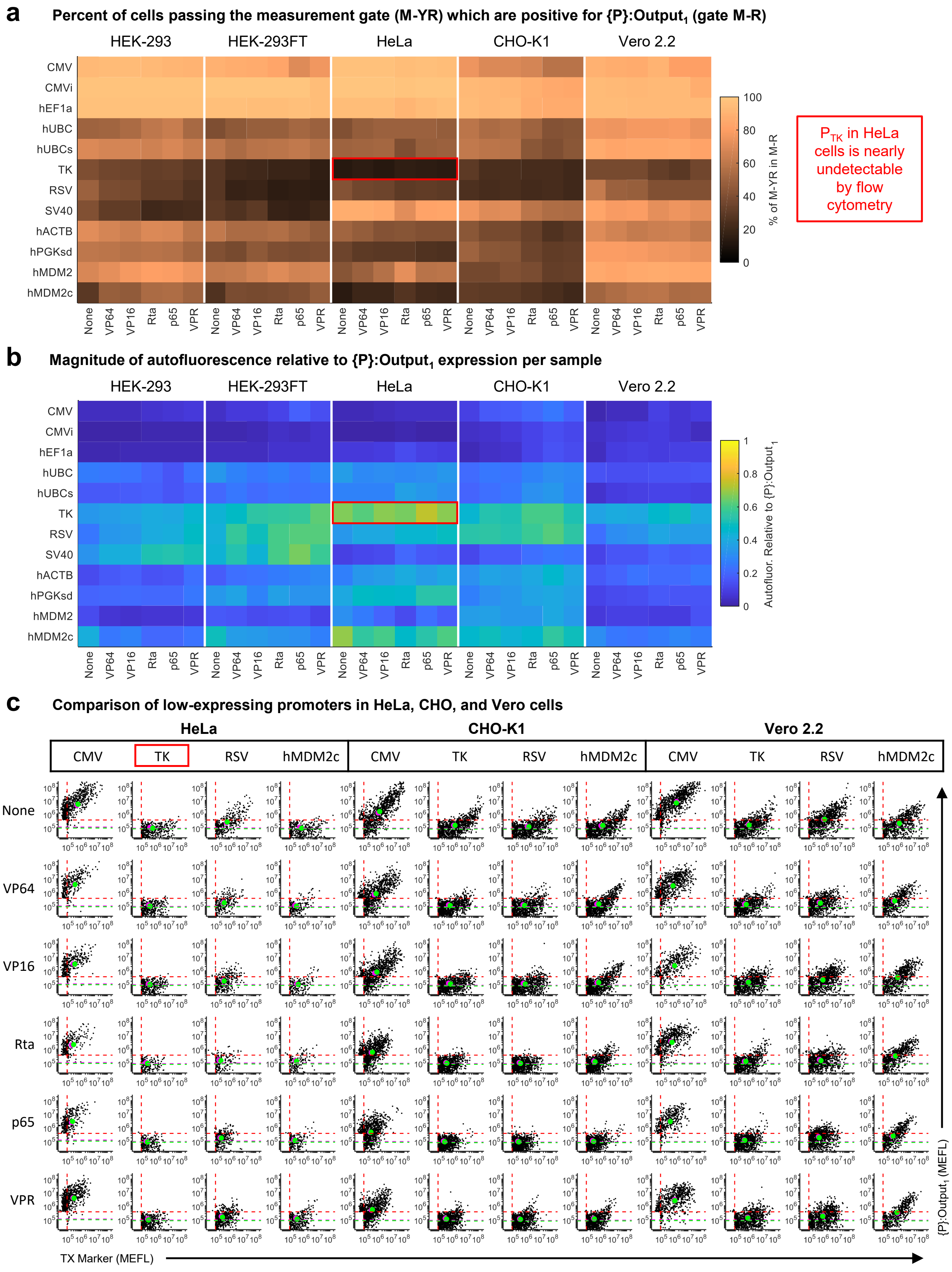

Supplementary Figure 43: Comparison of noise to output level for weak promoters.

Supplementary Figure 43: (Previous page.) **(a)** Percent of cells passing the measurement gate (*i.e.* positive for either {P}:Output_1 or TX Marker) that are positive for {P}:Output_1 for the data in Figure 2. Each percentage is the mean of three experimental replicates. **(b)** The relative magnitude of the background autofluorescence (defined as the median fluorescence in the same channel as {P}:Output_1 for cells not passing the measurement gate) compared to the level of {P}:Output_1. The relative magnitude is calculated by dividing the autofluorescence measurement by {P}:Output_1 in each sample. The relative magnitudes shown are the mean of measurements from three experimental repeats. **(c)** Scatterplots for weak promoters in HeLa, CHO-K1, and Vero 2.2 cells, with CMV as a strong promoter reference. The dashed red lines indicate the measurement gate for {P}:Output_1 and the TX Marker – only cells passing the gate are plotted. The magenta and green dashed lines show the median and geometric mean of the cells not passing the measurement gate (autofluorescence). The magenta and green dots show the median and geometric mean of the cells passing the measurement gate. The first experimental repeat is shown as a representative example. The medians shown in magenta dots correspond to the median measurements shown in Supplementary Figure 11b.

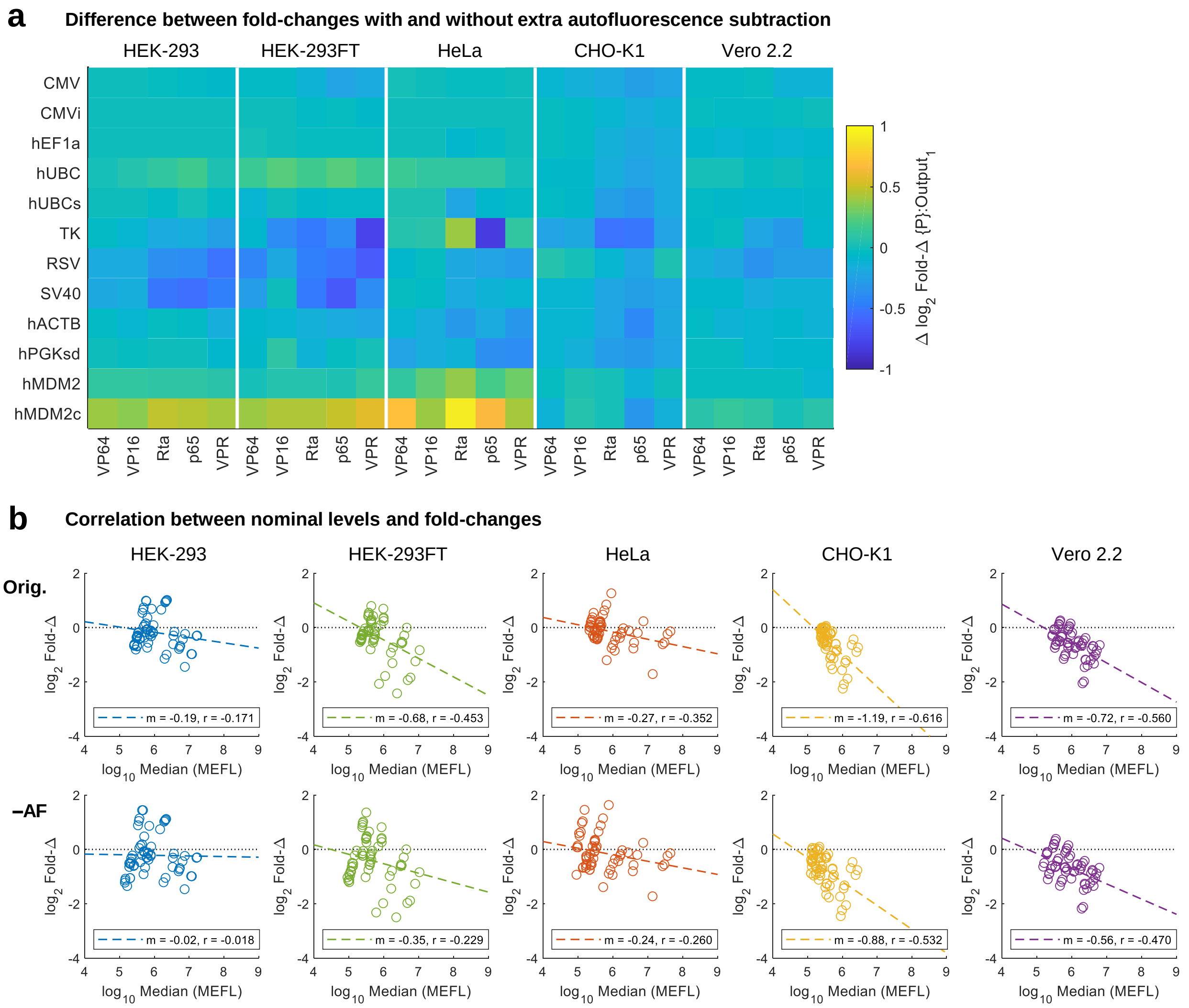

Supplementary Figure 44: Extra autofluorescence subtraction to reduce bias in the measurement of the output of weak promoters. **(a)** Differences (Δs) in $\log_2$ Fold-Δs for samples with and without subtraction of the autofluorescence (as defined in Supplementary Figure 43) from the median level of {P}:Output_1 prior to computing Fold-Δs for the data in Figure 2. The Δs represent the change from the non-subtracted data to the subtracted data, averaged across three experimental repeats. **(b)** Correlation between Nominal Output and Fold-Δ for samples in each cell line in the original data ('orig.') and the autofluorescence-subtracted data ('–AF'). Measurements from three experimental repeats are shown individually. m: slope, r: Pearson's correlation.

7 Supplementary Tables

**Promoter**	**Description**
CMV	Cytomegalovirus immediate-early promoter
CMVi	CMV IE promoter with an added intron from human betaherpesvirus 5
hEF1a	Human elongation factor 1 α promoter
hUBC	Human ubiquitin C promoter
hUBCs	hUBC promoter truncated to remove intron
TK	Herpes simplex virus (HSV)-1 thymidine kinase promoter
RSV	Rous Sarcoma Virus long terminal repeat
SV40	Simian virus 40 early promoter
hACTB	Human beta actin promoter
hPGKsd	Human phosphoglycerate kinase promoter with an added U2 splice donor
hMDM2	Human mouse double minute 2 homolog promoter
hMDM2c	Human MDM2 promoter truncated after the CpG island and appended with a splice acceptor to complete its intron

Supplementary Table 1: Description of constitutive promoters

Supplementary References

1. Farr, A. & Roman, A. A pitfall of using a second plasmid to determine transfection efficiency. *Nucleic Acids Research* **20,** 920 (1992).
2. Kelleher, R. J., Flanagan, P. M. & Kornberg, R. D. A novel mediator between activator proteins and the RNA polymerase II transcription apparatus. English. *Cell* **61,** 1209–1215 (1990).
3. Vojnic, E. *et al.* Structure and VP16 binding of the Mediator Med25 activator interaction domain. *Nature structural & molecular biology* **18,** 404–409 (2011).
4. Meyer, K. D., Lin, S.-C., Bernecky, C., Gao, Y. & Taatjes, D. J. p53 activates transcription by directing structural shifts in Mediator. *Nature structural & molecular biology* **17,** 753–760 (2010).
5. Hershey, J. W. B. Translational control in mammalian cells. *Annual Review of Biochemistry* **60,** 717–755 (1991).
6. Jagus, R., Anderson, W. F. & Safer, B. in *Progress in Nucleic Acid Research and Molecular Biology* 127–185 (Elsevier, 1981).
7. Sarkar, G., Edery, I., Gallo, R. & Sonenberg, N. Preferential stimulation of rabbit α globin mRNA translation by a cap-binding protein complex. *Biochimica et Biophysica Acta (BBA) - Gene Structure and Expression* **783,** 122–129 (1984).
8. Ray, B. K. *et al.* Role of mRNA competition in regulating translation: further characterization of mRNA discriminatory initiation factors. *Proceedings of the National Academy of Sciences* **80,** 663–667 (1983).
9. Schwanhäusser, B. *et al.* Global quantification of mammalian gene expression control. *Nature* **473,** 337–342 (2011).
10. Berger, S. L. *et al.* Genetic isolation of ADA2: a potential transcriptional adaptor required for function of certain acidic activation domains. *Cell* **70,** 251–265 (1992).
11. Gilbert, D. M., Heery, D. M., Losson, R., Chambon, P. & Lemoine, Y. Estradiol-inducible squelching and cell growth arrest by a chimeric VP16-estrogen receptor expressed in Saccharomyces cerevisiae: suppression by an allele of PDR1. *Molecular and Cellular Biology* **13,** 462–472 (1993).
12. Baron, U., Gossen, M. & Bujard, H. Tetracycline-controlled transcription in eukaryotes: Novel transactivators with graded transactivation potential. *Nucleic Acids Research* **25,** 2723–2729 (1997).
13. Lin, H., McGrath, J., Wang, P. & Lee, T. Cellular toxicity induced by SRF-mediated transcriptional squelching. *Toxicological Sciences* **96,** 83–91 (2007).
14. Corish, P. & Tyler-Smith, C. Attenuation of green fluorescent protein half-life in mammalian cells. *Protein Engineering, Design and Selection* **12,** 1035–1040 (1999).

15. Molinari, E., Gilman, M. & Natesan, S. Proteasome-mediated degradation of transcriptional activators correlates with activation domain potency in vivo. *EMBO Journal* **18,** 6439–6447 (1999).

16. Berger, S. L., Cress, W. D., Cress, A., Triezenberg, S. J. & Guarente, L. Selective inhibition of activated but not basal transcription by the acidic activation domain of VP16: Evidence for transcriptional adaptors. English. *Cell* **61,** 1199–1208 (1990).

17. Donahue, P. S. *et al.* COMET: A toolkit for composing customizable genetic programs in mammalian cells. *Nature Communications* **11,** 779 (2020).

18. Liang, S. D., Marmorstein, R., Harrison, S. C. & Ptashne, M. DNA sequence preferences of GAL4 and PPR1: how a subset of Zn2 Cys6 binuclear cluster proteins recognizes DNA. *Molecular and cellular biology* **16,** 3773–3780 (1996).

19. Natesan, S., Molinari, E., Rivera, V. M., Rickles, R. J. & Gilman, M. A general strategy to enhance the potency of chimeric transcriptional activators. *PNAS* **96,** 13898–13903 (1999).

20. Chavez, A. *et al.* Highly efficient Cas9-mediated transcriptional programming. *Nature Methods* **12,** 326–328 (2015).

21. Chavez, A. *et al.* Comparison of Cas9 activators in multiple species. *Nature Methods* **13,** 563–567 (2016).

22. Natesan, S., Rivera, V. M., Molinari, E. & Gilman, M. Transcriptional squelching re-examined. en. *Nature* **390,** 349–350 (1997).

23. Webster, G. A. & Perkins, N. D. Transcriptional Cross Talk between NF-κB and p53. *Molecular and Cellular Biology* **19,** 3485–3495 (1999).

24. Schmidt, S. F., Larsen, B. D., Loft, A. & Mandrup, S. Cofactor squelching: Artifact or fact? *BioEssays* **38,** 618–626 (2016).

25. Gam, J. J., DiAndreth, B., Jones, R. D., Huh, J. & Weiss, R. A 'poly-transfection' method for rapid, one-pot characterization and optimization of genetic systems. *Nucleic Acids Research* **47,** e106 (2019).

26. Bleris, L. *et al.* Synthetic incoherent feedforward circuits show adaptation to the amount of their genetic template. *Molecular Systems Biology* **7,** 519 (2011).

27. Strovas, T. J., Rosenberg, A. B., Kuypers, B. E., Muscat, R. A. & Seelig, G. MicroRNA-Based Single-Gene Circuits Buffer Protein Synthesis Rates against Perturbations. *ACS Synthetic Biology* **3,** 324–331 (2014).

28. Bartel, D. P. Metazoan MicroRNAs. *Cell* **173,** 20–51 (2018).

29. Liu, Q., Wang, F. & Axtell, M. J. Analysis of Complementarity Requirements for Plant MicroRNA Targeting Using a Nicotiana benthamiana Quantitative Transient Assay. *The Plant Cell* **26,** 741–753 (2014).

30. Michaels, Y. S. *et al.* Precise tuning of gene expression levels in mammalian cells. *Nature Communications* **10,** 818 (2019).

31. Ferreira, J. P., Overton, K. W. & Wang, C. L. Tuning gene expression with synthetic upstream open reading frames. *PNAS* **110,** 11284–11289 (2013).

32. Chung, H. K. *et al.* Tunable and reversible drug control of protein production via a self-excising degron. *Nature Chemical Biology* **11,** 713–720 (2015).

33. Lai, A. C. & Crews, C. M. Induced protein degradation: an emerging drug discovery paradigm. *Nature Reviews Drug Discovery* **16,** 101–114 (2017).

34. Li, S., Prasanna, X., Salo, V. T., Vattulainen, I. & Ikonen, E. An efficient auxin-inducible degron system with low basal degradation in human cells. *Nature Methods* **16,** 866–869 (2019).

35. Marzi, M. J. *et al.* Degradation dynamics of microRNAs revealed by a novel pulse-chase approach. *Genome Research* **26,** 554–565 (2016).

36. Haley, B. & Zamore, P. D. Kinetic analysis of the RNAi enzyme complex. *Nature Structural and Molecular Biology* **11,** 599–606 (2004).

37. Sashital, D. G., Jinek, M. & Doudna, J. A. An RNA-induced conformational change required for CRISPR RNA cleavage by the endoribonuclease Cse3. *Nature Structural and Molecular Biology* **18,** 680–687 (2011).

38. Wroblewska, L. *et al.* Mammalian synthetic circuits with RNA binding proteins for RNA-only delivery. *Nature Biotechnology* **33,** 839–841 (2015).

39. Castanotto, D. *et al.* Combinatorial delivery of small interfering RNAs reduces RNAi efficacy by selective incorporation into RISC. *Nucleic Acids Research* **35,** 5154–5164 (2007).

40. Boudreau, R. L., Martins, I. & Davidson, B. L. Artificial microRNAs as siRNA shuttles: improved safety as compared to shRNAs in vitro and in vivo. *Molecular Therapy* **17,** 169–175 (2009).

41. Piskacek, S. *et al.* Nine-amino-acid transactivation domain: Establishment and prediction utilities. *Genomics* **89,** 756–768 (2007).

42. Piskacek, M., Vasku, A., Hajek, R. & Knight, A. Shared structural features of the 9aaTAD family in complex with CBP. *Molecular BioSystems* **11,** 844–851 (2015).

43. Kiani, S. *et al.* CRISPR transcriptional repression devices and layered circuits in mammalian cells. *Nature Methods* **11,** 723–726 (2014).
